# Interaction with IP6K1 supports pyrophosphorylation of substrate proteins by the inositol pyrophosphate 5-InsP_7_

**DOI:** 10.1042/BSR20240792

**Published:** 2024-10-04

**Authors:** Aisha Hamid, Jayashree S. Ladke, Akruti Shah, Shubhra Ganguli, Monisita Pal, Arpita Singh, Rashna Bhandari

**Affiliations:** 1 Laboratory of Cell Signalling, Centre for DNA Fingerprinting and Diagnostics, Hyderabad 500039, India; 2 Graduate Studies, Regional Centre for Biotechnology, Faridabad 121001, Haryana, India

**Keywords:** cell signalling, Inositol pyrophosphates, IP6 kinases, metabolic messenger, post translational modification, Protein pyrophosphorylation

## Abstract

Inositol pyrophosphates (PP-InsPs) are a sub-family of water soluble inositol phosphates that possess one or more diphosphate groups. PP-InsPs can transfer their β-phosphate group to a phosphorylated Ser residue to generate pyrophosphorylated Ser. This unique post-translational modification occurs on Ser residues that lie in acidic stretches within an intrinsically disordered protein sequence. Serine pyrophosphorylation is dependent on the presence of Mg^2+^ ions, but does not require an enzyme for catalysis. The mechanisms by which cells regulate PP-InsP-mediated pyrophosphorylation are still unknown. We performed mass spectrometry to identify interactors of IP6K1, an enzyme responsible for the synthesis of the PP-InsP 5-InsP_7_. Interestingly, IP6K1 interacted with several proteins that are known to undergo 5-InsP_7_-mediated pyrophosphorylation, including the nucleolar proteins NOLC1, TCOF and UBF1, and AP3B1, the β subunit of the AP3 adaptor protein complex. The IP6K1 interactome also included CK2, a protein kinase that phosphorylates Ser residues prior to pyrophosphorylation. We observe the formation of a protein complex between IP6K1, AP3B1, and the catalytic α-subunit of CK2, and show that disrupting IP6K1 binding to AP3B1 lowers its in vivo pyrophosphorylation. We propose that assembly of a substrate-CK2-IP6K complex would allow for coordinated pre-phosphorylation and pyrophosphorylation of the target serine residue, and provide a mechanism to regulate this enzyme-independent modification.

## Introduction

Inositol pyrophosphates (PP-InsPs) are a sub-family of water soluble inositol phosphates that contain one or more energy-rich diphosphate moieties. Found in all eukaryotic organisms, the best studied PP-InsPs in mammals are 5-InsP_7_ (5-diphosphoinositol pentakisphosphate or 5PP-InsP_5_) and 1,5-InsP_8_ (bisdiphosphoinositol tetrakisphosphate or 1,5(PP)_2_-InsP_4_). The synthesis of 5-InsP_7_ from InsP_6_ (inositol hexakisphosphate) is catalyzed by IP6 kinases, of which there are three isoforms in mammals – IP6K1, IP6K2, and IP6K3 [1–3]. 5-InsP_7_ is a substrate for two mammalian PP-InsP_5_ kinases, which catalyze the synthesis of 1,5-InsP_8_ [4]. Designated as metabolic messengers, PP-InsPs are known to influence several cellular functions including phosphate homeostasis, apoptosis, vesicle trafficking, insulin secretion, ribosome biogenesis, and DNA repair [5–8].

PP-InsPs selectively regulate protein function using two molecular mechanisms – non-covalent binding, and covalent protein pyrophosphorylation [6,9]. PP-InsP-mediated serine pyrophosphorylation is a unique enzyme-independent post translational modification. During pyrophosphorylation, a PP-InsP transfers its β-phosphate to a pre-phosphorylated serine residue (pSer) on the substrate protein, that is located within an intrinsically disordered region in the vicinity of acidic Asp and Glu residues [9–12]. This phosphotransfer only takes place in the presence of divalent cations such as Mg^2+^. Although pre-phosphorylation of the target serine residues is brought about by acidophilic Ser/Thr kinases such as CK2, transfer of the β-phosphate from the donor PP-InsP to the acceptor pSer residue can take place without any enzyme. Using radiolabeling methods, 5-InsP_7_-mediated pyrophosphorylation has been demonstrated for *Saccharomyces cerevisiae* proteins Nsr1, Srp40, YGR130c, Rpa190, Rpa43, Rpa34, Apl6, and Gcr1, and mammalian proteins AP3B1, DIC2C, MYC, NOLC1, TCOF, and UBF1 [10–18]. Recently, 148 sites of pyrophosphorylation were identified in 71 human proteins by a mass spectrometry based proteomics analysis, revealing the nucleolar proteins NOLC1 and TCOF to possess the highest number of pyrophosphosites [12].

The biological consequences of PP-InsP-mediated pyrophosphorylation have been reported in the case of a few protein substrates. Pyrophosphorylation of the budding yeast transcription factor Gcr1 destabilizes its interaction with its partner Gcr2, stalling the transcription of essential glycolytic enzymes and lowering the rate of glycolysis [15]. Also in budding yeast, 5-InsP_7_ can pyrophosphorylate three subunits in RNA polymerase I – Rpa190, Rpa43, and Rpa34. Yeast lacking Kcs1, the enzyme responsible for 5-InsP_7_ synthesis, exhibit reduced rRNA synthesis, leading to defects in ribosome biogenesis and impaired protein translation [18]. The inhibition of IP6 kinases also resulted in reduced rRNA synthesis in mammalian cells, potentially a consequence of lowering pyrophosphorylation of nucleolar proteins involved in rDNA transcription [12]. 5-InsP_7_-mediated pyrophosphorylation of three mammalian proteins, the dynein-intermediate chain (DIC2C), the oncoprotein MYC, and the β-subunit of the AP3 adaptor protein (AP3B1), have been shown to regulate their interaction with other proteins. Pyrophosphorylation at Serine51 on DIC2C promotes its interaction with the p150Glued subunit of dynactin, enhancing recruitment of the dynein motor to vesicles [17]. MYC undergoes pyrophosphorylation in its central PEST domain [16], enhancing its binding to the E3 ubiquitin ligase FBW7, which in turn promotes MYC polyubiquitination and turnover. Pyrophosphorylation of AP3B1 disrupts its binding to the microtubule plus-end directed kinesin motor protein KIF3A [13]. As binding of the AP3 complex with KIF3A promotes intracellular trafficking and subsequent release of HIV1 Gag protein, AP3B1 pyrophosphorylation lowers the release of HIV-1 virus-like particles from mammalian cells.

Our understanding of the mechanistic details of phosphotransfer from PP-InsP to a target protein is still nascent. This enzyme-independent phosphate transfer is supported by a high concentration of Mg^2+^ in the buffer, and increasing temperature [9–11]. Other essential factors are acidic amino acid residues flanking the target pSer residue, and the presence of this target sequence within an intrinsically disordered region of the substrate protein [9]. InsP_6_ and InsP_7_ have been shown to bind proteins with comparable specificity and affinity [19,20], as these molecules are similar in their structure and charge, barring the presence of a β-phosphate in InsP_7_. Excess InsP_6_ has been shown to inhibit in vitro phosphotransfer from 5-InsP_7_ to a protein substrate [10]. As cells are reported to harbor a 5- to 50-fold higher intracellular concentration of InsP_6_ compared with PP-InsPs [21], binding of InsP_6_ to a target protein may inhibit its intracellular pyrophosphorylation by PP-InsPs. The data presented in our study addresses this concern. We show that the protein interactome of IP6K1 includes several pyrophosphorylation substrate proteins. IP6K1 also interacts with the protein kinase CK2, which is known to pre-phosphorylate serine residues targeted for pyrophosphorylation by 5-InsP_7_ [11]. We show that IP6K1 forms a multi-protein complex with AP3B1 and CK2. Binding of IP6K1 with AP3B1 is primarily via an intrinsically disordered region in the N-terminal lobe of IP6K1. Over-expression of this IP6K1 fragment disrupts the interaction of IP6K1 with AP3B1, and lowers endogenous AP3B1 pyrophosphorylation. Binding of IP6 kinase with the pyrophosphorylation substrate protein may provide a mechanism to increase the local concentration of 5-InsP_7_ and counter any inhibition of pyrophosphorylation by InsP_6_. Formation of a substrate-CK2-IP6K complex would also facilitate coordinated pre-phosphorylation and pyrophosphorylation of the target serine residue, providing cells with a mechanism to control this enzyme-independent modification.

## Materials and methods

### Reagents

The primary antibodies used in the present study for immunoblotting (IB) or immunoprecipitation (IP), along with antibody dilution for each application, and supplier (including catalogue number), were as follows: anti-IP6K1 (Sigma-Aldrich, HPA040825; IB, 1:3000; Genetex, GTX103949; IB, 1:3000); anti-GAPDH (Sigma-Aldrich, G8795; IB, 1:10,000); anti-α-Tubulin (Sigma-Aldrich, T9026; IB, 1:10,000); anti-V5 tag (Thermo Fisher Scientific, R960-25; IB, 1:7000; IP, 1 μg); anti-HA (Sigma-Aldrich, H6908; IP, 1 μg, IB: 1:7000); anti-FLAG (Sigma-Aldrich, F1804; IB, 1:10,000; IP, 1 μg); anti-myc tag (Sigma-Aldrich, M4439; IB, 1:10,000; IP, 1 μg); anti-AP3B1 (Bethyl labs, 50-157-0195, IB, 1:2000); anti-CK2α (GeneTex, GTX107576, IB, 1:2000); anti-NOLC1 (Abclonal, A5899, IB, 1:500); anti-TCOF (Abclonal, A2512, IB, 1:1000); anti-UBF1 (Santa Cruz, sc-13125, IB, 1:500). For immunoprecipitation of endogenous IP6K1, an in-house antibody generated against the N-terminal region of IP6K1 was used [22]. PVDF membrane for protein transfer, Glutathione-sepharose beads, Protein A-sepharose beads, Protein G-sepharose beads, Streptavidin-sepharose beads, and ECL prime chemiluminescence substrate were procured from GE Healthcare. The Gateway Clonase enzymes were purchased from Thermo Fisher Scientific. NuPAGE 4–12% Bis-Tris gels, 20X MES running buffer, and 4X LDS sample buffer were purchased from Thermo Fisher Scientific. [γ-^32^P]ATP (LCP-101) was procured from JONAKI/BRIT. *myo*-2-[^3^H] inositol (15-20 Ci/mmol) (ART 0116B) was procured from American Radiolabeled Chemicals. Ultima-Flo AP (6013599) was purchased from Perkin-Elmer. Casein Kinase II (CK2 holoenzyme) (P6010) was purchased from New England Biolabs. Casein Kinase II inhibitor III, TBCA (218710) was purchased from Calbiochem.

### Plasmids

Human IP6K1 cDNA (GenBank ID NM_001242829.2) and catalytically inactive IP6K1 (K226A) mutant were subcloned in pCDNA3.1 as previously described [22]. cDNA encoding human IP6K1 domains, i.e., FL, N-lobe, C-lobe, IDR-1, IDR-2, PDKG motif with IP helices, and IDR-1 with C-lobe, were cloned into the N-terminal SFB (S protein, Flag, streptavidin binding peptide)-tag destination vector, using the Gateway cloning strategy (Thermo Fisher Scientific). Mouse full-length MYC cDNA with a C-terminal V5 tag was cloned in pCDNA3.1 as previously described [16]. pZW12 CK2β was a gift from David Litchfield (Addgene plasmid 27088; GenBank ID NM_001320.7). Human AP3B1 (GenBank ID NM_003664.5) cDNA (a gift from Solomon Snyder, Johns Hopkins School of Medicine, Baltimore, U.S.A.) was subcloned into SalI and NotI restriction enzyme sites into the pCMV-Myc plasmid vector to express myc epitope-tagged AP3B1. Human CK2α cDNA in the pCMV-HA vector was a gift from Solomon Snyder. Plasmid expressing HA-tagged CK2α (K68M) was subcloned from pGV15-CK2α-K68M (a gift from David Litchfield, Addgene plasmid 27089) into SalI and NotI restriction enzyme sites in the pCMV-HA vector.

### Cell culture and transfection

The HEK293T cell line was obtained from the laboratory of Solomon Snyder at Johns Hopkins School of Medicine, Baltimore, U.S.A., and was authenticated by Lifecode Technologies Private Limited, New Delhi, India. Short tandem repeat profiling for authentication showed a 94.1% match with the ATCC reference genotype (https://www.atcc.org/products/crl-3216). HEK293T cells were grown in a humidified incubator with 5% CO_2_ at 37°C in Dulbecco’s modified Eagle’s medium (DMEM) supplemented with 10% fetal bovine serum, 1 mM L-glutamine, 100 U/ml penicillin, and 100 µg/ml streptomycin. For transfection of HEK293T cells, polyethylenimine (PEI) (Polysciences, 23966) was used at a ratio of 1:3 (DNA:PEI). All plasmids used for transfection were purified using the Plasmid Midi kit (Qiagen). Cells were harvested 36–48 h post-transfection for further analyses. For experiments with TBCA treatment, HEK293T cells were transfected with myc-AP3B1 with or without CK2α. Twelve hours post transfection, cells were treated with 50 µM TBCA or DMSO as a vehicle control for 16 h. Post treatment, cells were harvested in 1X Laemmli buffer and the samples were analyzed by immunoblotting.

### Tandem affinity purification and mass spectrometry analysis

HEK293T cells transiently transfected to express either SFB-tagged GFP or SFB-tagged IP6K1 were lysed in NETN buffer (20 mM Tris-HCl, pH 8.0, 100 mM NaCl, 1 mM EDTA, 0.5% Nonidet P-40) containing protease inhibitor cocktail (Sigma-Aldrich, P8340) and phosphatase inhibitor cocktail (Sigma-Aldrich, P5762), at 4°C for 60 min. Cell debris were removed by centrifugation at 14000 × ***g*** for 10 min, and cell lysates were incubated with streptavidin-sepharose beads (Cytiva Life Sciences) for 1 h at 4°C with end-over-end rotation. The bound protein complexes were washed thrice with NETN buffer and then eluted with 2 mg/ml biotin (Merck) for 90 min at 4°C. The eluates were incubated with S-protein agarose beads (Novagen) for 1 h at 4°C and then washed thrice with NETN buffer. Proteins bound to S-protein agarose beads were boiled in 2X SDS sample buffer for 5 min, loaded on a 12% SDS polyacrylamide gel, allowed to run into the resolving gel up to 1 cm, and visualized by Coomassie Brilliant Blue staining. All proteins in the sample were excised in one gel slice and sent for mass spectrometry analysis to Taplin Biological Mass Spectrometry Facility at Harvard University, U.S.A. Briefly, gel pieces were subjected to in-gel digestion with sequencing-grade trypsin (Promega), and the extracted peptides were resolved by reverse phase HPLC. Eluted peptides were subjected to electrospray ionization (ESI) and then allowed to enter an LTQ Orbitrap Velos Pro ion-trap mass spectrometer (Thermo Fisher Scientific). Peptides were detected, isolated, and fragmented to produce a tandem mass spectrum of specific fragment ions for each peptide. Peptide sequences (and hence protein identity) were determined by matching protein databases from UniProt (https://www.uniprot.org/taxonomy/10090) with the acquired fragmentation pattern by the software program, Sequest (Thermo Fisher Scientific). Mass spectromery data were submitted to the MassIVE repository, a full member of the Proteome Xchange Consortium. Data can be accessed via the URL <https://massive.ucsd.edu>, with the data set identifier MassIVE MSV000092218. The total peptide number for each protein, which reflects relative protein abundance in the sample, was compared between the test (SFB-IP6K1) and control (SFB-GFP) samples using the CRAPome tool (https://reprint-apms.org) [23]. Empirical Fold Change Score (FC-A and FC-B) were used to compare the enrichment of proteins in the bait (SFB-IP6K1) over user control (SFB-GFP) in replicates. A fold change score (FCB) of 1.4 was used as a cut-off score to select genuine interactors of IP6K1. The selected proteins were examined for Gene Ontology (GO) term enrichment using the Functional Annotation Clustering tool at Database for Annotation, Visualization and Integrated Discovery (DAVID) v6.7 (https://david.ncifcrf.gov) [24].

### Co-immunoprecipitation assay

HEK293T cells were collected 48 h post transfection, lysed for 1 h at 4°C in lysis buffer (50 mM HEPES, pH 7.4, 100 mM NaCl, 1 mM EDTA, 0.5% Nonidet P-40), protease inhibitor cocktail and phosphatase inhibitor cocktail. The lysates were incubated with specific antibody overnight at 4°C with end-over-end mixing. The complex was pulled down using Protein A or Protein G-sepharose beads (GE Healthcare) pre-equilibrated in lysis buffer for 2 h. The beads were washed thrice with lysis buffer followed by boiling in 1X Laemmli buffer. The samples were analyzed by immunoblotting with primary antibodies (details are provided above). For SFB-tagged protein pull-down, streptavidin-sepharose beads were added to the cell lysate for 2 h at 4°C followed by washing and boiling in 1X Laemmli buffer, and analysis by immunoblotting. UVITEC Alliance Q9 documentation system or the GE ImageQuant LAS 500 imager were used for chemiluminescence detection. Fiji ImageJ software [25] was used for densitometry based quantitative analysis. For quantification of protein levels, band intensities were normalized to that of the loading control. For quantification of the extent of co-immunoprecipitation, band intensities of the co-precipitating proteins were normalized to intensity of the immunoprecipitated protein.

### Purification and pull-down with GST fusion proteins

GST-tagged IP6K1 and CK2α were expressed in *Escherichia coli* BL21 (DE3) strain and affinity chromatography was performed using standard protocols. Cells were grown in LB medium, and protein expression was induced with the addition of 0.5 mM IPTG overnight at 18°C. Cultures were pelleted by centrifuging at 6000 × ***g*** and pellets were lysed by sonication in ice-cold buffer A (20 mM HEPES, pH 6.8, 100 mM NaCl, 2 mM EDTA, and 5 mM DTT). The lysates were centrifuged at 18000 × ***g*** and the supernatant was incubated with equilibrated glutathione-sepharose beads at 4°C for 2 h. Beads were washed twice with Buffer C (20 mM HEPES, pH 6.8, 500 mM NaCl, 2 mM EDTA, 1% Triton X-100) and twice with Buffer B (20 mM HEPES, pH 6.8, 100 mM NaCl, 2 mM EDTA, 1% Triton X-100). The beads were resuspended in an equal volume of 1X PBS and 20 μl slurry was analysed by SDS-PAGE to determine the amount of purified protein, with a known amount of BSA for comparison. Purified GST, or GST-tagged IP6K1 or CK2α immobilized on glutathione-sepharose beads were incubated with HEK293T cell lysates in buffer containing 50 mM HEPES-KOH, pH 7.4, 100 mM NaCl, 5 mM MgCl_2_, 0.3% Triton X-100, and protease and phosphatase inhibitor cocktails, for 2 h at 4°C. The interacting proteins were identified by immunoblotting.

### Analysis of cellular inositol pyrophosphates

IP6K1 knockout HEK293T cells, transfected to express either active or catalytically inactive IP6K1, were labeled with [^3^H]-inositol as described earlier [26,27]. Cells seeded in 60 mm dishes in normal growth medium were allowed to attain 30% confluence and then transferred to inositol-free DMEM (MP Biomedicals, D9802-06.25) containing 10% dialysed fetal bovine serum, and labeled with 30 µCi *myo*-2-[^3^H] inositol for 2.5 days. The media was removed and fresh media containing *myo*-2-[^3^H] inositol (30 µCi) was added for another 2.5 days. At the end of 5 days, when isotopic labeling is achieved, cells were washed and collected by scraping in chilled PBS. From the cell pellet, soluble inositol phosphates were extracted by the addition of 350 µl extraction buffer (0.6 M HClO_4_, 2 mM EDTA, 0.2 mg/ml phytic acid) (Sigma-Aldrich, P8810) on ice for 15–20 min, followed by centrifugation at 21,000 × ***g*** for 10 min. The supernatant containing soluble inositol phosphates was collected, and lipid inositols in the pellet were extracted with 1 ml lipid extraction buffer (0.1 N NaOH and 0.1% Triton X-100) at room temperature with end-over-end mixing for 4–5 h. The soluble inositol phosphate extract was mixed with ∼120 µl neutralization solution (1 M K_2_CO_3_ and 5 mM EDTA). Tubes were left open on ice for 1 h, followed by centrifugation at 21,000 × *g* for 10 min at 4°C. The extracted inositol phosphates were resolved by HPLC (5125 HPLC pumps, Waters) on a Partisphere SAX column (4.6 mm × 125 mm, HiChrome) using a gradient of Buffer A (1 mM EDTA) and Buffer B (1 mM EDTA and 1.3 M (NH_4_)_2_HPO_4_ (pH 3.8)) as follows: 0–5 min, 0% B; 5–10 min, 0–20% B; 10–70 min, 20–100% B; 70–80 min, 100% B. Approximately 1 ml fractions containing soluble inositol phosphates were mixed with 3 ml scintillation cocktail (Ultima-Flo AP) and counted for 5 min in a liquid scintillation counter (Tri-Carb 2910 TR, Perkin Elmer). The lipid inositol extract was counted in a liquid scintillation counter, and the soluble inositol phosphate count in each fraction was normalized to this.

### Protein phosphorylation and pyrophosphorylation

For the phosphorylation reactions, myc-tagged AP3B1 immunoprecipitated and immobilised on Protein G-sepharose beads was incubated with CK2 (250 units; New England Biolabs) or GST-CK2α overexpressed and purified from *E. coli* BL21 (DE3) strain, for 30 min, in the presence of protein kinase buffer (New England Biolabs) with 3 µCi [γ-^32^P]ATP, and 500 µM Mg^2+^-ATP. Beads were washed with ice-cold PBS and eluted in 1X LDS sample buffer followed by boiling at 95°C for 5 min. Proteins were resolved by a 4–12% NuPAGE Bis-Tris gel and transferred to a PVDF membrane. Blots were dried and subjected to autoradiography, followed by immunoblotting to detect myc-tagged AP3B1. For inhibition of CK2 activity, enzyme was preincubated with 15 µM TBCA for 30 min on ice. Post incubation, the in vitro phosphorylation reaction was conducted as described above.

The synthesis of 5[β-^32^P]InsP_7_, and pyrophosphorylation of proteins expressed in HEK293T cells using radiolabeled 5-InsP_7_ have been described previously [12,16,17,28]. Briefly, myc-tagged AP3B1 expressed transiently in HEK293T cells was immunoprecipitated using a myc-tag antibody and immobilized on protein G-sepharose beads. The immunoprecipitated complexes were resuspended in pyrophosphorylation buffer (25 mM HEPES-KOH pH 7.4, 50 mM NaCl, 6 mM MgCl_2_, 1 mM DTT) in the presence of 3 µCi 5[β-^32^P]InsP_7_, and incubated at 37°C for 15 min. The beads were boiled in 1X LDS sample buffer at 95°C for 5 min, resolved by 4–12% NuPAGE Bis-Tris gel and transferred to a PVDF membrane. The blots were exposed for ∼5 days to a phosphorimager screen. Phosphorylation and pyrophosphorylation of AP3B1 were detected using a phosphorimager (Typhoon FLA-9500). The ratio of radiolabeled protein to the total immunoprecipitated protein was quantified by densitometry analysis using the Fiji ImageJ software.

### Statistical analysis

GraphPad Prism 8 was used to perform statistical analyses and prepare graphs. Western blotting and co-immunoprecipitation data were quantified as described earlier [29]. The normalized intensity values were expressed relative to the control sample in each blot, which was considered as ‘1’. The number of biologically independent replicates (*N*) for each experiment are indicated in the figure legends. *P*-values are from a one-sample *t*-test. *P*≤0.05 was considered statistically significant.

## Results

### Identification of the IP6K1 protein interactome

**Figure 1:**
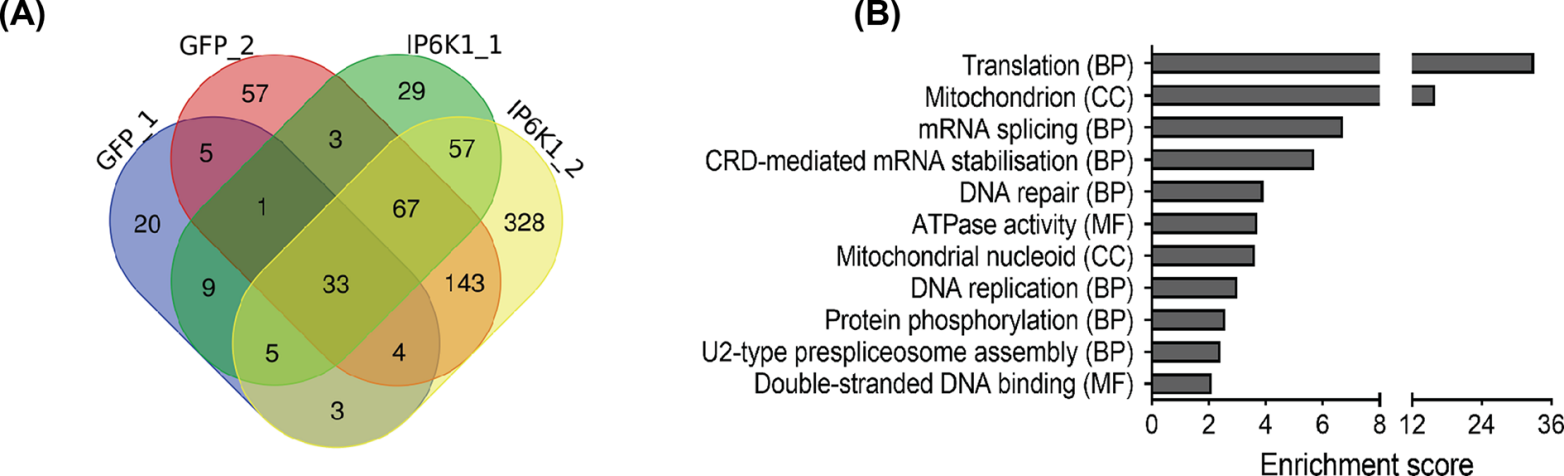
Analysis of the IP6K1 protein interactome (**A**) Venn diagram depicting the number of proteins identified in each replicate of SFB-tagged GFP (control) or SFB-tagged IP6K1. A total of 414 proteins that interacted specifically with IP6K1 were absent from the GFP pull-down. (**B**) The total peptide counts for each protein bound to replicate test (IP6K1) and control (GFP) samples was analyzed using the CRAPome tool; proteins that show a fold change (FC-B) score of ≥ 1.4 were subjected to Functional Annotation Clustering of Gene Ontology (GO) terms using the DAVID tool (Supplementary Table S3). The graph represents the clusters of Cellular Component (CC), Biological Process (BP) and Molecular Function (MF) GO terms with a group enrichment score ≥ 2 (*P*≤0.01).

To identify the specific interacting partners of IP6K1, SFB-tagged IP6K1 was over-expressed in HEK293T cells and subjected to affinity purification under non-denaturing conditions, followed by mass spectrometry. The protein interactome of SFB-tagged GFP served as a negative control (Supplementary Table S1). Pull-down and mass spectrometry analysis for both IP6K1 and GFP was conducted in replicate sets (Figure 1A). A total of 414 proteins were found to specifically bind IP6K1, of which 57 were present in both replicate sets (Figure 1A). This data was subjected to the CRAPome analysis pipeline (reprint-apms.org/) to distinguish between authentic and contaminant interactors by comparing the total peptide number for each interacting protein bound to the test sample (IP6K1) with the control sample (GFP) (Supplementary Table S2). To avoid missing any genuine IP6K1 interactors, we analyzed 414 proteins identified in either SFB-IP6K1 replicate set instead of focusing only on the 57 interactors found in both replicates. In the list of specific IP6K1 interacting proteins, we noted the presence of previously verified binding partners of IP6K1. These include DDB1, which contributes to a role for IP6K1 in CRL4 signalosome assembly [30], and EIF4G1, which is important for P-body formation [22]. Additional proteins in the IP6K1 interactome have previously been identified in high-throughput studies on protein–protein interactions [31,32]. We set the stringent fold change FC-B score [23] to 1.4 to accommodate the verified IP6K1 interactor EIF4G1 [22], and subjected 179 IP6K1 interacting proteins that fall in this range to Gene Ontology (GO) term enrichment analysis using the DAVID tool (https://david.ncifcrf.gov) [24]. IP6K1 interacting partners were found to be involved in several biological processes including translation, mRNA splicing, and DNA repair (Figure 1B).

Interestingly, the IP6K1 interactome contained 5-InsP_7_ pyrophosphorylation substrates including AP3B1, NOLC1, TCOF, and UBF1 (Table 1 and Supplementary Table S2) [10–13]. Among the interactors we also observed the catalytic α and α’ (CSK21 and CSK22), and regulatory β (CSK2B) subunits of CK2 (Table 1 and Supplementary Table S2), which is the primary protein kinase responsible for pre-phosphorylation of proteins prior to their pyrophosphorylation by 5-InsP_7_ [11,13,14,16,17].

**Table 1:** Serine pyrophosphorylation targets and priming kinase in the IP6K1 interactome

Protein ID/(*Gene ID*)	Uniprot ID	SFB-IP6K1 Replicate 1	SFB-IP6K1 Replicate 2
AP3B1/(*AP3B1*)	O00203	9	7
NOLC1/(*NOLC1*)	Q14978	0	1
TCOF/(*TCOF1*)	Q13428	1	17
UBF1/(*UBTF*)	P17480	3	0
CSK21/(*CSNK2A1*)	P68400	9	8
CSK22/(*CSNK2A2*)	P19784	3	0
CSK2B/(*CSNK2B*)	P67870	0	3

The table indicates the total peptide numbers for each protein identified in replicate pull-downs of SFB-tagged IP6K1. There were no peptides corresponding to these proteins in the SFB-GFP pull-down (negative control). Only known pyrophosphorylation substrate proteins, and the subunits of CK2 are shown.

### IP6K1 interacts with 5-InsP_7_ substrate proteins

**Figure 2:**
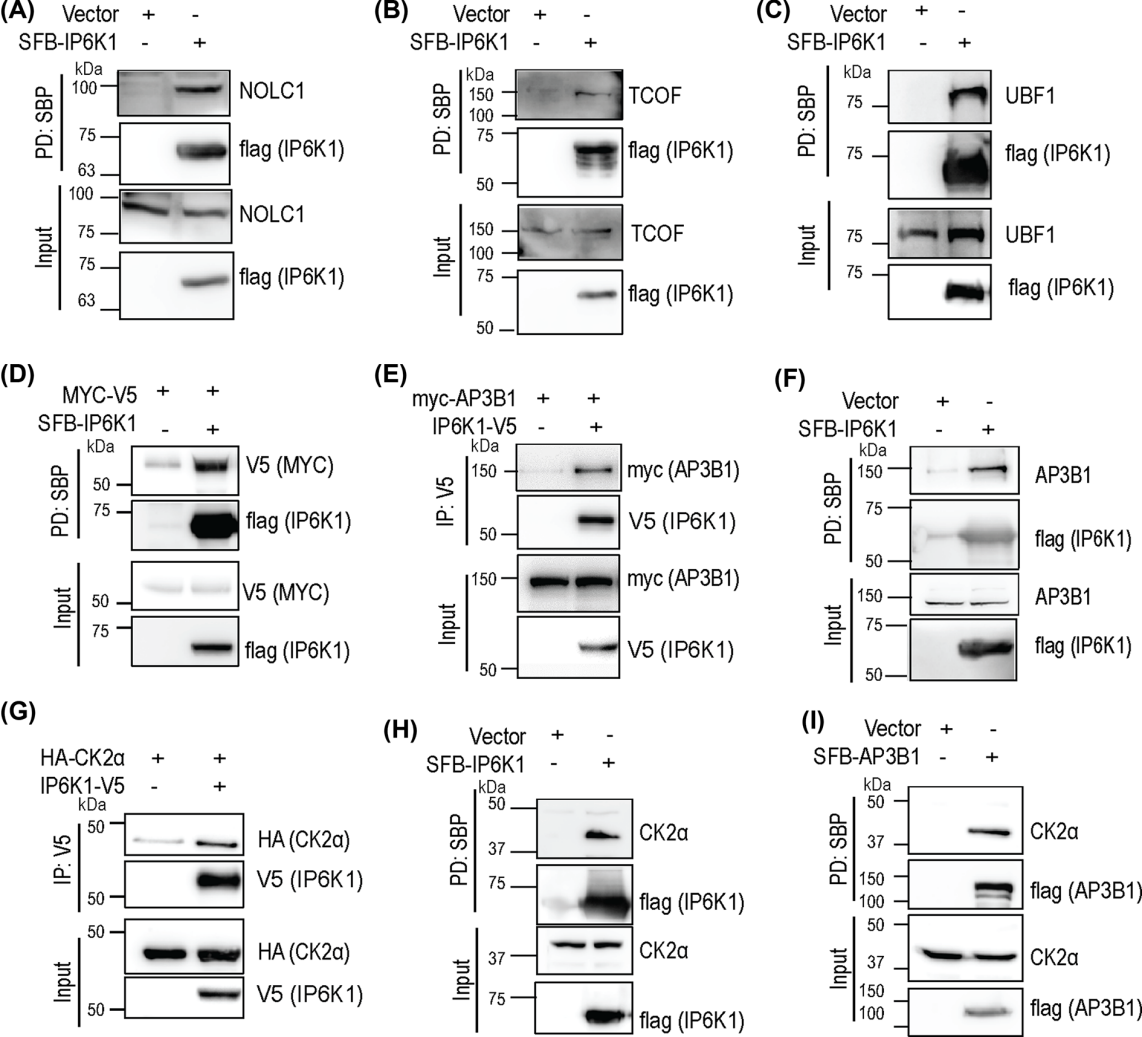
IP6K1 binds pyrophosphorylation substrate proteins and CK2 (**A–C**) Co-precipitation of endogenous NOLC1 (A), TCOF (B) or UBF1 (C) with IP6K1. N-terminally SFB-tagged IP6K1 was expressed in HEK293T cells, pulled down with streptavidin-sepharose beads, and probed to detect endogenous NOLC1, TCOF or UBF1. (**D**) Co-precipitation of mouse MYC protein with IP6K1. N-terminally SFB-tagged IP6K1 and C-terminally V5-tagged MYC were co-expressed in HEK293T cells, pulled down with streptavidin-sepharose beads and probed to detect the V5 tag. (**E**) Co-immunoprecipitation of AP3B1 with IP6K1. C-terminally V5-tagged IP6K1 and N-terminally myc-tagged AP3B1 were transiently co-expressed in HEK293T cells, immunoprecipitated with an anti-V5 antibody, and probed to detect the myc epitope (**F**) Co-precipitation of endogenous AP3B1 with IP6K1. N-terminally SFB-tagged IP6K1 was overexpressed in HEK293T cells, pulled down with streptavidin-sepharose beads, and probed to detect endogenous AP3B1 (**G**) Co-immunoprecipitation of CK2α with IP6K1. C-terminally V5-tagged IP6K1 and N-terminally HA-tagged CK2α were co-expressed in HEK293T cells, immunoprecipitated with an anti-V5 antibody, and probed to detect the HA tag (**H**) Co-precipitation of endogenous CK2α with IP6K1. N-terminally SFB-tagged IP6K1 was overexpressed in HEK293 cells, pulled down with streptavidin-sepharose beads, and probed to detect endogenous CK2α. (**I**) Co-precipitation of endogenous CK2α with AP3B1. N-terminally SFB-tagged AP3B1 was overexpressed in HEK293 cells, pulled down with streptavidin-sepharose beads, and probed to detect endogenous CK2α. An anti-Flag tag antibody was used to detect SFB-tagged proteins. Where indicated, cells were transfected with an empty vector as a control. All figures are representative of three independent experiments.

To validate the binding of IP6K1 to pyrophosphorylation substrate proteins, we expressed tagged IP6K1 in HEK293T cells, and performed co-immunoprecipitation analyses. IP6K1 was able to pull down endogenous NOLC1 and TCOF (Figure 2A,B), the two most extensively pyrophosphorylated proteins identified in human cells [12]. UBF1, another nucleolar 5-InsP_7_ pyrophosphorylation substrate protein [12], also co-precipitated with IP6K1 (Figure 2C). We have earlier shown that the oncoprotein MYC is a substrate for pyrophosphorylation by 5-InsP_7_ synthesized by IP6K1 [16]. Although MYC was not present in the IP6K1 interactome, perhaps due to its short half-life, IP6K1 was able to pull-down co-overexpressed MYC (Figure 2D). We also validated binding of the adaptor protein subunit AP3B1 to IP6K1. Both overexpressed and endogenous AP3B1 co-precipitated with IP6K1 in HEK293T cells (Figure 2E,F). Next, we confirmed the interaction of IP6K1 with protein kinase CK2. Human CK2 is a heterotetramer consisting of two copies each of the catalytic α-subunit (sometimes replaced by the α′-subunit) and the regulatory β-subunit [33]. IP6K1 was able to pull-down co-overexpressed and endogenous CK2 α-subunit (CK2α) (Figure 2G,H). We also detected the interaction of endogenous CK2α with AP3B1 (Figure 2I). In summary, we observed the binding of IP6K1 to several 5-InsP_7_ pyrophosphorylation substrate proteins – AP3B1, NOLC1, UBF1, TCOF and MYC, and to CK2α. As AP3B1 is known to be pre-phosphorylated by CK2 prior to its pyrophosphorylation, and the effect of pyrophosphorylation on AP3B1 function has been clearly demonstrated [13], we chose to further study the influence of AP3B1-CK2-IP6K1 complex formation on AP3B1 pyrophosphorylation.

### IP6K1 binds directly to AP3B1 and indirectly to CK2

**Figure 3:**
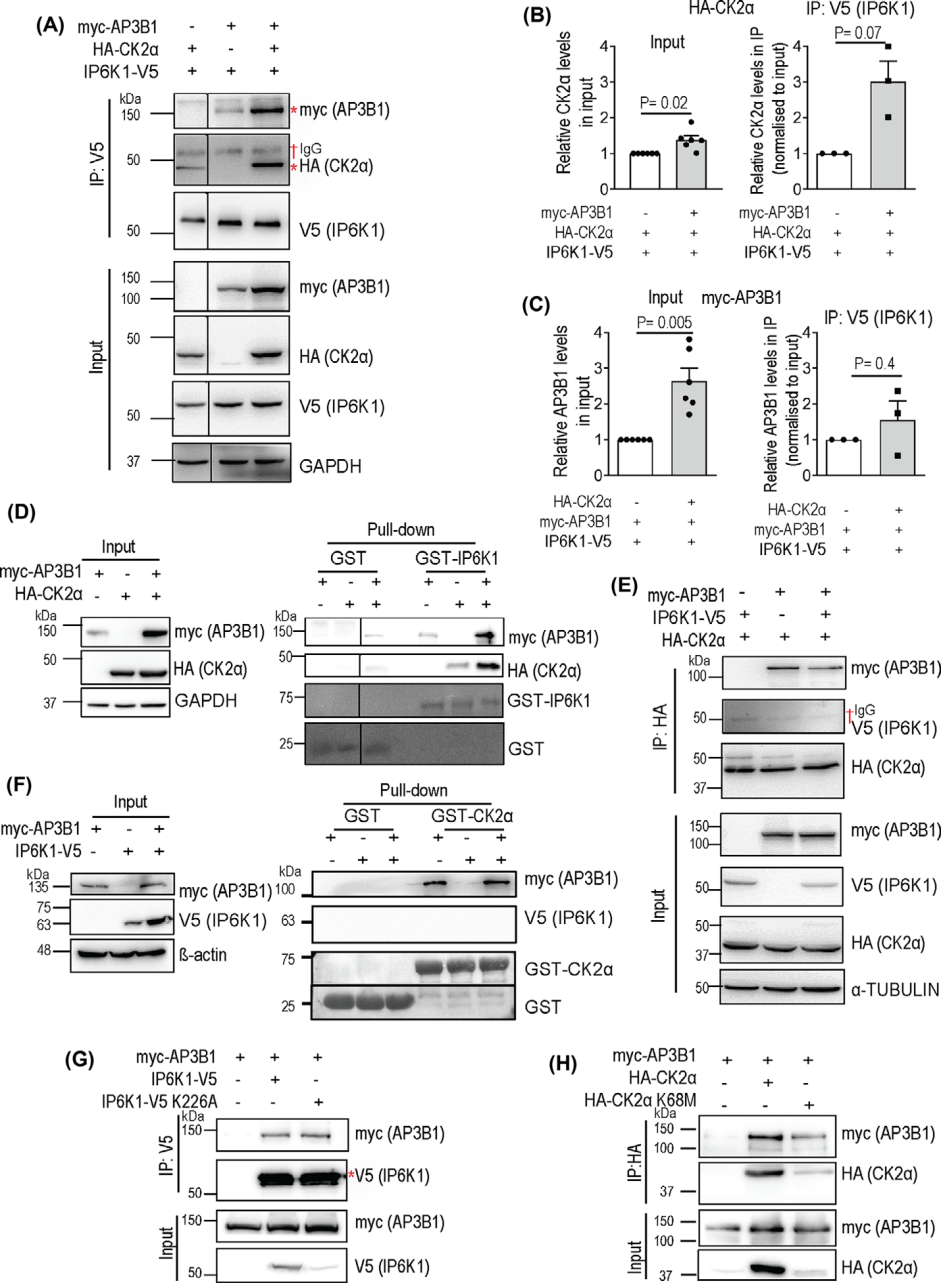
IP6K1 binds CK2 via AP3B1 (**A**) Representative immunoblots showing the effect of CK2α and AP3B1 co-expression on the extent of pull-down by IP6K1. V5-tagged IP6K1 was co-expressed with HA-tagged CK2α and/or myc-tagged AP3B1 in HEK293T cells. Lysates were immunoprecipitated with an anti-V5 antibody and probed to detect the HA or myc epitopes. (**B**) Cell lysates from (A) were quantified for HA-CK2α expression level (Input) and the extent of co-immunoprecipitation with IP6K1-V5 (IP) in the presence or absence of co-expressed myc-AP3B1. Data (mean ± SEM) were analyzed using a one-sample *t*-test (*N* = 6 for Input and *N* = 3 for IP). (**C**) Cell lysates from (A) were quantified for myc-AP3B1 expression level (Input) and the extent of co-immunoprecipitation with IP6K1-V5 (IP) in the presence or absence of co-expressed HA-CK2α. Data (mean ± SEM) were analyzed using a one-sample *t*-test (*N* = 6 for Input and *N* = 3 for IP). (**D**) Pull-down of AP3B1 and CK2α by purified IP6K1. GST or GST-IP6K1 purified from *E. coli* and immobilized on glutathione-sepharose beads was incubated with lysates from HEK293T cells transfected with HA-tagged CK2α and/or myc-tagged AP3B1. HA- and myc-tagged proteins were detected by immunoblotting and GST-tagged proteins were detected by Ponceau S staining. (**E**) Representative immunoblots showing the effect of AP3B1 and IP6K1 co-expression on the extent of pull-down by CK2α. HA-tagged CK2α was co-expressed with myc-tagged AP3B1 and/or V5-tagged IP6K1 in HEK293T cells. Lysates were immunoprecipitated with an anti-HA antibody and probed to detect the V5 or myc epitopes. IP6K1 could not be detected in the precipitate. (**F**) Pull-down of AP3B1 and IP6K1 by purified CK2α. GST or GST-CK2α purified from *E. coli* and immobilized on glutathione-sepharose beads was incubated with lysates from HEK293T cells transfected with V5-tagged IP6K1 and/or myc-tagged AP3B1. V5- and myc-tagged proteins were detected by immunoblotting and GST-tagged proteins were detected by Ponceau S staining. (**G**) Co-precipitaion of AP3B1 with either active or catalytically inactive IP6K1. N-terminally myc-tagged AP3B1 was co-expressed with C-terminally V5-tagged active or catalytically inactive IP6K1 in HEK293T cells, immunoprecipitated with an anti-V5 antibody, and probed to detect myc-AP3B1. (**H**) Co-precipitaion of AP3B1 with either active or catalytically inactive CK2α. N-terminally myc-tagged AP3B1 was co-expressed with N-terminally HA-tagged active or catalytically inactive CK2α in HEK293T cells, immunoprecipitated with an anti-HA antibody, and probed to detect the myc-AP3B1. A dagger symbol (†) corresponds to the IgG band and an asterisk (*) marks the specific band of interest. The vertical lines in (A) and (D) indicate the removal of nonessential lanes from a single original blot for better depiction; the horizontal black line in (F) indicates cropping of the intervening region in the same blot. Data presented in (D–H) are representative of three independent experiments.

We examined the expression level and the extent of co-precipitation of AP3B1 or CK2α with IP6K1. IP6K1 was able to pull-down both CK2α and AP3B1, independently, and when these proteins were co-expressed (Figure 3A). There was a marginal increase in the level of CK2α when it was co-overexpressed with AP3B1 (Figure 3A,B). However, when corrected for this increase in expression, the extent of binding of CK2α to IP6K1 was enhanced ∼3-fold when AP3B1 was co-expressed, suggesting that IP6K1 binds CK2α via AP3B1 (Figure 3A,B). Interestingly, the expression of AP3B1 increased by ∼2.6 fold when CK2α was co-expressed, and IP6K1 expression was held constant across both samples (Figure 3A,C). When normalized to increased AP3B1 input levels, there was no further effect of the presence of CK2α on the co-precipitation of AP3B1 with IP6K1, suggesting a direct interaction between AP3B1 and IP6K1, independent of CK2 (Figure 3A,C). To verify these findings, we assessed AP3B1 and CK2α binding with GST-tagged recombinant IP6K1 expressed in *E. coli*. GST or GST-IP6K1 immobilized on glutathione-sepharose beads were incubated with lysates from HEK293T cells expressing CK2α and/or AP3B1. GST-IP6K1 bound independently with CK2α and AP3B1, but the extent of binding with CK2α was significantly enhanced in the presence of AP3B1 (Figure 3D). The apparent increase in GST-IP6K1 binding with AP3B1 in the presence of CK2α could be attributed to increased expression of AP3B1 in cells co-expressing CK2α (also see Figure 3A,C). Next, we examined the binding of AP3B1 and IP6K1 to immobilized CK2α. Although we observed robust pull-down of AP3B1 with CK2α in HEK293T cell lysates, we could not detect any IP6K1 signal in the CK2α immunoprecipitate even in the presence of AP3B1 (Figure 3E). We then incubated recombinant GST-tagged CK2α with HEK293T cell extracts expressing IP6K1 or AP3B1 independently or together. Whereas AP3B1 showed robust binding to GST-CK2α, we did not detect any IP6K1 in the pull-down (Figure 3F). This suggests that IP6K1 may be a weak and indirect binder of CK2α, competed out by other more abundant CK2α interactors in the cell lysate. The indirect binding of CK2α to IP6K1 in the absence of co-expressed AP3B1 may have been facilitated by other endogenous IP6K1 interacting partners in the cell extract (Figure 3A,D).

To examine whether the interaction between AP3B1 and IP6K1 is influenced by the ability of IP6K1 to synthesize 5-InsP_7_, we compared the extent of pull-down of AP3B1 with catalytically active or inactive (Lys226Ala; [22]) IP6K1 (Figure 3G). We observed that the binding between AP3B1 and IP6K1 is independent of IP6K1 activity. Similarly, we tested whether the protein kinase activity of CK2α influences its interaction with AP3B1. We observed no difference in the extent of binding of AP3B1 to active or catalytically inactive (Lys68Met; [34]) forms of CK2α (Figure 3H). Together, these data suggest that in the tripartite protein complex formed by IP6K1, AP3B1, and CK2, IP6K1 binds directly with AP3B1, and indirectly with CK2 via AP3B1, and that the activity of CK2 or IP6K1 does not influence this interaction.

### CK2 phosphorylates and regulates the level of AP3B1

**Figure 4:**
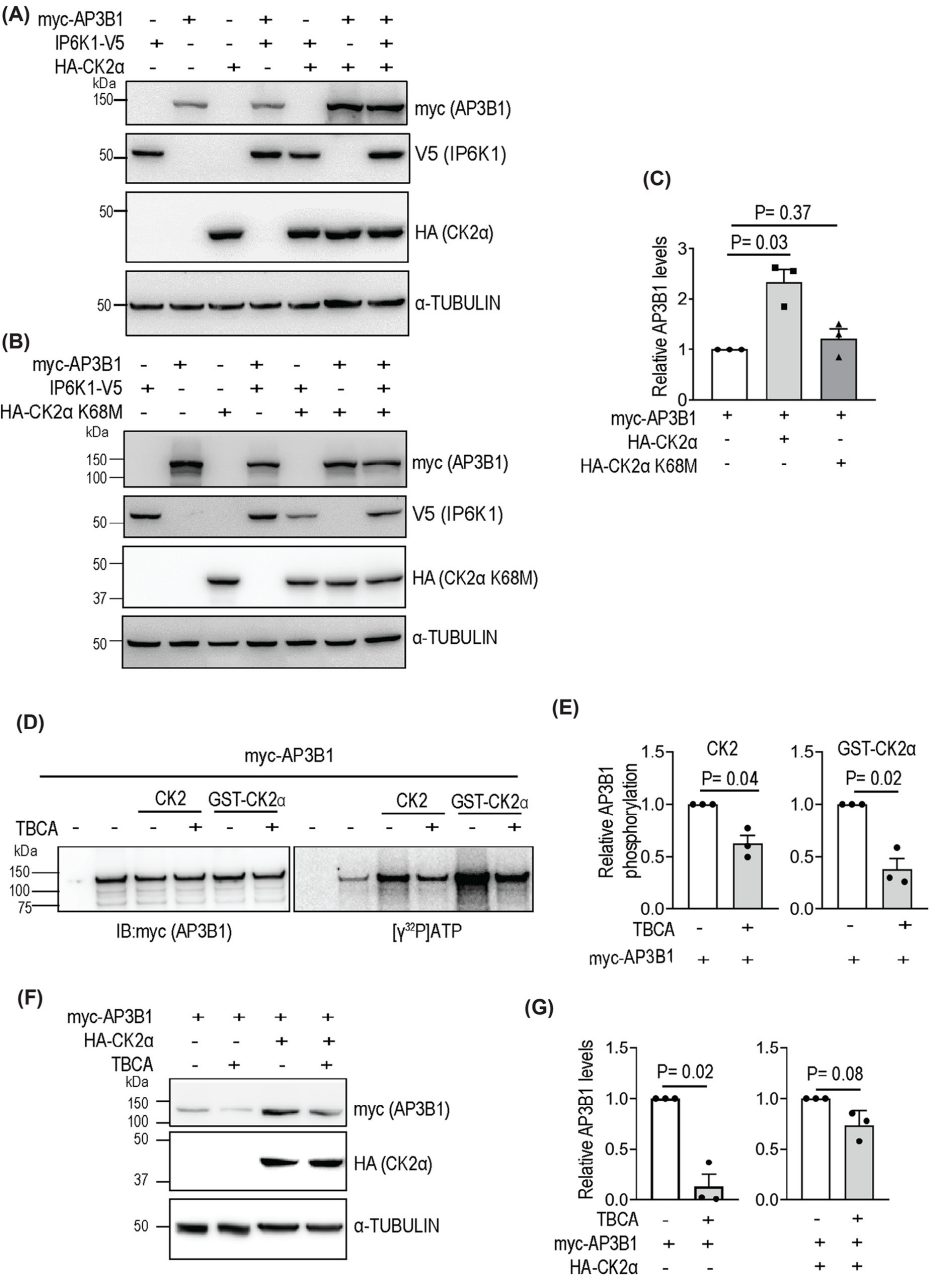
CK2 phosphorylates and elevates AP3B1 levels (**A,B**) Effect of CK2 activity on the level of AP3B1. Myc-tagged AP3B1, V5-tagged IP6K1, and active (A) or catalytically inactive Lys68Met (B) HA-tagged CK2α were expressed in HEK293T cells individually or in combination. Lysates were immunoblotted to detect myc, V5 and HA epitopes. (**C**) Quantification of (A) and (B), where the scatter bar graph represents mean fold change ± SEM in the levels of myc-AP3B1, when co-expressed with native CK2α or inactive CK2α K68M, in the absence of IP6K1, normalized to the loading control. Data were analyzed using a one-sample *t*-test (*N*=3). (**D**) Phosphorylation of AP3B1 by CK2. Myc-tagged AP3B1 immunoprecipitated from HEK293T cells was incubated with CK2 holoenzyme or GST-CK2α and radiolabeled [γ-^32^P]ATP in the absence or presence of 15 µM TBCA (CK2 inhibitor). DMSO was used as a vehicle control. Images show immunoblotting with a myc-tag antibody (left) and autoradiography to detect phosphorylation (right). Cells transfected with an empty-vector were used as a negative control. (**E**) Quantification of (D), where the scatter bar graph represents mean fold change ± SEM in the extent of phosphorylation of myc-AP3B1 in the presence or absence of TBCA. Data were analyzed using a one-sample *t*-test (*N*=3). (**F**) Effect of TBCA treatment on cellular levels of AP3B1. Myc-tagged AP3B1 was expressed with or without HA-tagged CK2α in HEK293T cells, followed by treatment with 50 µM TBCA (16 h) or DMSO as a vehicle control. Lysates were immunoblotted to detect myc and HA epitopes, and tubulin as a loading control. (**G**) Quantification of (F), where the scatter bar graph represents mean fold change ± SEM in the levels of AP3B1 in TBCA treated compared with untreated cells, normalized to the loading control. Data were analyzed using a one-sample *t*-test (*N*=3).

We next determined whether the protein kinase activity of CK2 is essential for the observed increase in AP3B1 levels upon co-expression with CK2α (Figure 3A,C). The level of AP3B1 increased upon co-expression of active CK2α, but inactive CK2α had no effect on AP3B1 expression (Figure 4A,B – compare lanes 2 and 6; quantified in Figure 4C), confirming that CK2α regulates AP3B1 via its kinase activity. Co-expression of IP6K1 had no impact on the levels of AP3B1 or CK2α.

To validate the phosphorylation of AP3B1 by CK2, we immunoprecipitated myc-tagged AP3B1 and incubated it with CK2 holoenzyme or purified CK2α subunit in the presence of radiolabeled [γ-^32^P]ATP. We observed robust phosphorylation of AP3B1 by CK2, which was lowered when the CK2 inhibitor TBCA was added to the reaction (Figure 4D,E) [35]. TBCA treatment of HEK293T cells led to a significant drop in the levels of AP3B1, but in cells co-expressing CK2α along with AP3B1, there was a marginal impact of TBCA on AP3B1 (Figure 4F,G). The data presented here suggest that phosphorylation of AP3B1 by CK2 directly upregulates its intracellular levels, but we cannot rule out the possibility that CK2 may indirectly up-regulate AP3B1 by acting on other protein targets.

### IP6K1 binds AP3B1 via an intrinsically disordered region in its N-lobe

**Figure 5:**
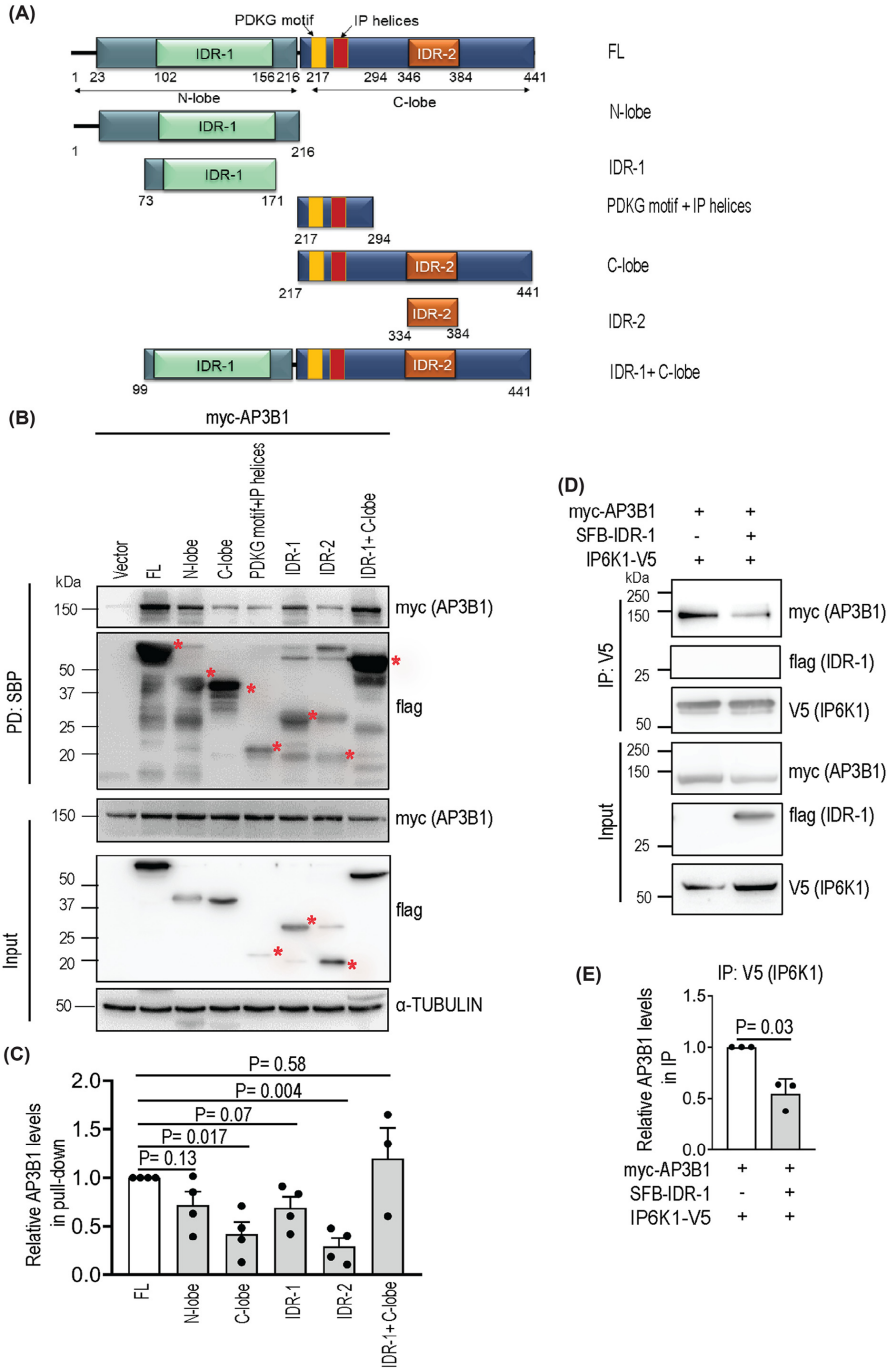
IP6K1 binds AP3B1 via its IDR-1 motif (**A**) Domain map of human IP6K1 fragments used in this study. The first and last amino acid residue numbers of each construct are indicated. (**B**) Pull-down of AP3B1 with IP6K1 fragments. SFB-tagged IP6K1 fragments, co-expressed in HEK293T cells with myc-tagged AP3B1, were pulled down with streptavidin-sepharose beads and probed to detect the myc epitope. IP6K1 fragments were probed using an anti-Flag antibody (asterisks [*] mark the specific bands of interest). (**C**) Quantification of (B) showing the extent of pull-down of AP3B1 with IP6K1 fragments compared with full-length IP6K1. Data (mean ± SEM) were analyzed using a one-sample *t*-test (*N*=4). (**D**) Effect of IDR-1 overexpression on IP6K1-AP3B1 interaction. V5-tagged IP6K1 and myc-tagged AP3B1 were co-expressed in HEK293T cells in the absence or presence of SFB-tagged IDR-1 fragment of IP6K1, immunoprecipitated with an anti-V5 antibody, and probed to detect the myc epitope. (**E**) Quantification of (D) showing the extent of co-immunoprecipitation of AP3B1 with IP6K1, in the absence or presence of SFB-IDR-1. Data (mean ± SEM) were analyzed using a one-sample *t*-test (*N*=3).

The predicted structure of IP6K1 is broadly divided into amino (N-) terminal and carboxyl (C-) terminal lobes [36] (https://alphafold.ebi.ac.uk/entry/Q92551), and contains an intrinsically disordered region (IDR) within each lobe (IUPred3; https://iupred3.elte.hu/). Within the C-lobe lie the D[I/V]K[I/L]G motif and IP helices, which are conserved across the family of IP kinases, and are involved in binding with the substrate InsP_6_ [36,37]. We generated expression constructs corresponding to different fragments of IP6K1 spanning the N-lobe, C-lobe, a segment spanning the PDKG motif and IP helices, and intrinsically disordered regions designated IDR-1 and IDR-2, that lie within the N- and C-lobes respectively (Figure 5A). SFB-tagged IP6K1 fragments were co-overexpressed with AP3B1, and subjected to pull-down using streptavidin-sepharose beads. The extent of binding of AP3B1 with IP6K1 fragments corresponding to the N-lobe, IDR-1, and IDR-1 + C-lobe was comparable with binding to full-length IP6K1, whereas the C-lobe and IDR-2 fragments showed significantly lower interaction with AP3B1 (Figure 5B,C). These data suggest that IP6K1 primarily interacts with AP3B1 via the long disordered region within its N-lobe. To confirm this, we monitored the effect of IDR-1 expression on the interaction between full-length IP6K1 and AP3B1. The extent of co-precipitation of AP3B1 with IP6K1 was reduced by ∼50% in the presence of IDR-1 (Figure 5C,D), suggesting that IDR-1 expression has a dominant negative effect on the binding of IP6K1 with AP3B1.

### Binding of IP6K1 to AP3B1 assists its pyrophosphorylation *in vivo*

**Figure 6:**
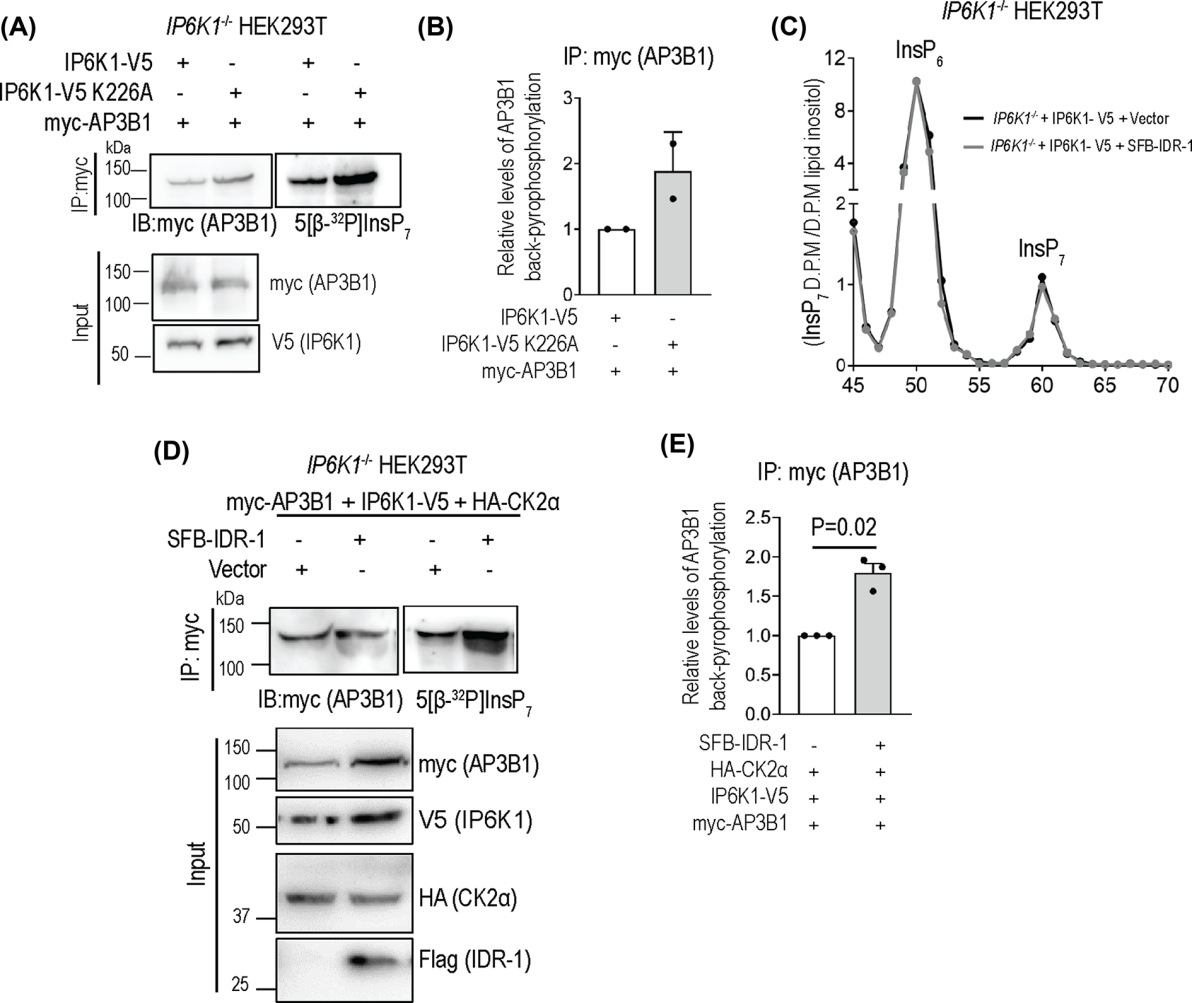
Pyrophosphorylation of AP3B1 is reduced by destabilizing protein complex formation (**A**) Back-pyrophosphorylation assay to demonstrate in vivo AP3B1 pyrophosphorylation. Myc-tagged AP3B1 co-expressed with either active or inactive V5-tagged IP6K1 in *IP6K1^−/−^* HEK293T cells was immunoprecipitated, incubated with 5[β-^32^P]InsP_7_, resolved on a 4–12% NuPAGE Bis-Tris gel and transferred to a PVDF membrane. Membranes were subjected to autoradiography to detect pyrophosphorylation (right) and immunoblotting to detect the myc epitope (left). Input levels of all the co-expressed proteins are shown. (**B**) Quantification of (A) showing the fold change (mean ± range) in the extent of back-pyrophosphorylation of myc-AP3B1, normalized to its immunoprecipitated protein levels, when co-expressed with inactive *vs* active IP6K1 (*N*=2). (**C**) HPLC profiles of [^3^H] inositol labeled *IP6K1^−/−^* HEK293T cells overexpressing V5-tagged IP6K1 with SFB-IDR-1 or a vector control. Soluble inositol phosphate counts were normalized to the total lipid inositol count for each sample. Peaks corresponding to InsP_6_ and InsP_7_ are indicated. Data are representative of two independent experiments. (**D**) Back-pyrophosphorylation assay to monitor effect of IDR-1 on in vivo AP3B1 pyrophosphorylation. Myc-tagged AP3B1, was co-expressed in *IP6K1^−/−^* HEK293T cells with V5-tagged IP6K1 and HA-tagged CK2α, in the presence or absence of SFB-tagged IDR-1. AP3B1 was immunoprecipitated with the myc-tag antibody and subjected to pyrophosphorylation in presence of 5[β-^32^P]InsP_7_ as in (A). Images show immunoblotting to detect the myc epitope (left) and autoradiography to detect pyrophosphorylation (right). Input levels of all the co-expressed proteins are shown. (**E**) Quantification of (**D**) showing the fold change in the extent of back-pyrophosphorylation of myc-AP3B1, normalized to its immunoprecipitated protein levels, when expressed in the absence or presence of IDR-1. Data (mean ± SEM) were analyzed using a one-sample *t*-test (*N*=3).

Finally, we monitored how formation of a protein complex between IP6K1 and AP3B1 affects intracellular pyrophosphorylation of AP3B1 by 5-InsP_7_. AP3B1 has been shown to be pyrophosphorylated by radiolabeled 5-InsP_7_ within its hinge region (residues 576-902) [13]. To establish that AP3B1 is indeed pyrophosphorylated by 5-InsP_7_ synthesized by IP6K1 inside mammalian cells, we conducted a ‘back-pyrophosphorylation’ assay [12,17,28]. This technique compares the extent of *in vitro* pyrophosphorylation of a substrate protein isolated from cells with different intracellular levels of 5-InsP_7_. A target protein isolated from cells with lower 5-InsP_7_ levels would exist in a hypo-pyrophosphorylated state compared with the same protein isolated from cells with higher 5-InsP_7_ levels. Therefore, in the presence of radiolabeled 5-InsP_7_, the hypo-pyrophosphorylated protein obtained from 5-InsP_7_ deficient cells would accept a higher amount of radiolabeled phosphate compared with the protein isolated from 5-InsP_7_ abundant cells. To conduct this assay, we utilized IP6K1 knockout HEK293T cells expressing either catalytically active or inactive (Lys226Ala) IP6K1. We have earlier shown that IP6K1 knockout cells expressing inactive IP6K1 have 80% lower 5-InsP_7_ levels compared with cells expressing the active form of the kinase [12]. AP3B1 over-expressed under these two conditions was immunoprecipitated and incubated with radiolabeled 5-InsP_7_. There was a ∼2-fold higher extent of back-pyrophosphorylation on AP3B1 isolated from 5-InsP_7_-deficient cells compared with AP3B1 from 5-InsP_7_ sufficient cells (Figure 6A,B). This result firmly established that AP3B1 does indeed undergo intracellular pyrophosphorylation by 5-InsP_7_ synthesized by IP6K1.

Next, we relied on a similar back-pyrophosphorylation method to determine whether the binding between IP6K1 and AP3B1 aids intracellular pyrophosphorylation of AP3B1. For this, we used IDR-1 to disrupt the interaction between AP3B1 and full-length IP6K1. We first confirmed that over-expression of IDR-1 along with IP6K1 does not alter the total cellular levels of 5-InsP_7_ (Figure 6C). We then co-overexpressed IP6K1, AP3B1 and CK2α in the presence or absence of the IP6K1 IDR-1 fragment in IP6K1 knockout HEK293T cells. The lowered binding between IP6K1 and AP3B1 in cells expressing IDR-1 (Figure 5D) is likely to reduce the local synthesis of 5-InsP_7_ in the vicinity of AP3B1. We observed a ∼2-fold increase in back-pyrophosphorylation of AP3B1 isolated from cells over-expressing IDR-1 compared with control cells (Figure 6D,E). This data confirms that the dominant negative effect of IDR-1 on formation of the IP6K1-AP3B1 complex hinders in vivo pyrophosphorylation of AP3B1.

## Discussion

**Figure 7:**
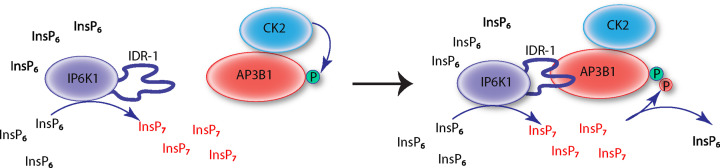
Formation of a protein complex facilitates 5-InsP_7_-mediated pyrophosphorylation The α-subunit of protein kinase CK2 binds to AP3B1 and catalyzes its phosphorylation, priming it for 5-InsP_7_-mediated pyrophosphorylation. IP6K1 interacts with AP3B1 via its IDR-1 region and synthesizes 5-InsP_7_ in the vicinity of the complex. A local increase in the concentration of 5-InsP_7_ promotes the mass-action driven transfer of its β-phosphate moiety to pyrophosphorylate AP3B1.

PP-InsP-mediated pyrophosphorylation has been studied for some years, but intracellular processes that influence this post-translational modification are not clearly understood. Here, we provide proof that the 5-InsP_7_ synthesizing enzyme IP6K1 binds to several proteins that are known to undergo 5-InsP_7_-mediated pyrophosphorylation. We demonstrate direct binding of the pyrophosphorylation substrate AP3B1 to a disordered region in the N-lobe of IP6K1, and to the α-subunit of protein kinase CK2. Phosphorylation of serine residues on AP3B1 by CK2 would prime them for pyrophosphorylation. The local synthesis of 5-InsP_7_ by IP6K1 bound to AP3B1 would increase the relative abundance of 5-InsP_7_ over InsP_6_ and promote mass action driven pyrophosphorylation of the prephosphorylated serine residues on AP3B1 (Figure 7). Binding of IP6K1 with AP3B1 may therefore contribute to disruption of the KIF3A-AP3 interaction lowering the release of virus particles from the cell. In this way, formation and dissolution of a tripartite complex between the pyrophosphorylation substrate, the priming protein kinase, and the 5-InsP_7_ synthesizing IP6 kinase, would provide cells with a mechanism to control enzyme-independent serine pyrophosphorylation by 5-InsP_7_.

Although an enzyme is not needed to catalyze the phosphotransfer from a PP-InsP to pSer, the presence of excess Mg^2+^ is essential for the reaction [9]. Both CK2 and IP6K1 require Mg^2+^ as a co-factor for enzyme activity [1,38], but the role of Mg^2+^ in the formation of the IP6K1-AP3B1-CK2 complex, if any, is still unclear. It is likely that Mg^2+^ ions coordinate with PP-InsP and Asp/Glu residues near the target pSer to bridge the two ‘substrates’ in the absence of a classical ‘enzyme’. Intrinsic disorder in the surrounding region may play an important role in facilitating access of the target pSer to the Mg^2+^-PP-InsP complex [9]. The data presented here show that a disordered region in the N-lobe of IP6K1 binds AP3B1. This region of intrinsic disorder is present in all three IP6K isoforms, and is the most variable sequence between them, possibly conferring differential binding specificity to the individual IP6Ks. Disordered sequences are known to provide scaffolds that facilitate protein-protein interaction [39,40]. Binding of the IDR regions in IP6Ks with the disordered pyrophosphorylation substrate sequence could be a conserved mechanism to bring these proteins together and facilitate 5-InsP_7_-mediated pyrophosphorylation.

One additional problem presented by the absence of an enzyme to catalyze pyrophosphorylation is that the target sequence would just as easily complex with Mg^2+^-InsP_6_ as it would with Mg^2+^-PP-InsP. In most organisms and cell types studied till date, the intracellular abundance of InsP_6_ exceeds that of PP-InsPs [6,21]. Given that transfer of the β-phosphate from a PP-InsP to a pSer is likely a reaction driven by crowding of reactants, the relative local abundance of PP-InsP over InsP_6_ could control the extent of intracellular protein pyrophosphorylation. Our data showing that IP6K1 binds to many known pyrophosphorylated proteins suggests that local synthesis of 5-InsP_7_ by an IP6 kinase bound to the pyrophosphorylation substrate may be a general mechanism to overcome the problem of excess InsP_6_ over PP-InsPs.

While this work has extensively characterized the interaction between AP3B1, IP6K1 and CK2, a similar complex may be formed between other pyrophosphorylation substrates, IP6K1 (or other IP6 kinases) and CK2 (or other acidophilic protein kinases). We have shown that IP6K1 binds the nucleolar proteins NOLC1, TCOF and UBF1, and the nuclear oncoprotein MYC, all of which are documented substrates for pyrophosphorylation [12,16]. IP6K1 is present in the cytoplasm and nucleus (https://www.proteinatlas.org/ENSG00000176095-IP6K1), and it is shown to co-localize with UBF1 in the nucleolus, where many other pyrophosphorylated proteins are found [12]. In addition to these validated interactors, we noted that the IP6K1 interactome includes several other pyrophosphorylated proteins identified by mass spectrometry (Supplementary Table S2). CK2 is expressed in most human cells and tissues, and is up-regulated in cancer cells [41,42]. It is enriched in the nucleus (https://www.proteinatlas.org/ENSG00000113712-CSNK1A1), where it may pre-phosphorylate several proteins to prime them for pyrophosphorylation. CK2 is known to target Ser/Thr residues with an acidic Glu/Asp at the +3 position [43,44]. Our data shows that CK2 phosphorylation increases the level of AP3B1. We do not yet know whether the phosphosites on AP3B1 responsible for this increase overlap with or are distinct from the sites that undergo subsequent pyrophosphorylation. Nevertheless, regulation of CK2 activity and subcellular localization may provide another handle for cells to control pyrophosphorylation. Interestingly, NOLC1 (also called Nopp140) has been shown to bind to CK2α and inhibit its catalytic activity, and InsP_6_ binding to CK2α at the same site reverses this inhibition [45]. 5-InsP_7_ synthesized by IP6K2 binds CK2 and promotes its kinase activity [30]. The regulation of CK2 activity by the binding of InsP_6_ or 5-InsP_7_, and competition with negatively charged protein substrates, would add another layer of complexity to the modulation of protein pyrophosphorylation by formation of a substrate-CK2-IP6K complex.

## Supplementary Material

Supplementary Tables S1-S3

## Data Availability

The mass spectrometry data used in this manuscript have been deposited to the MassIVE, a full member of the Proteome Xchange Consortium and can be accessed through Data set identifier ID- MassIVE MSV000092218 (https://massive.ucsd.edu).

## Abbreviations

ESI: electrospray ionization
IDR: intrinsically disordered region
PP-InsP: inositol pyrophosphate
InsP _6_: inositol hexakisphosphate
5-InsP _7_: 5-diphosphoinositol pentakisphosphate
