# Supplementary material for: Interaction with IP6K1 supports pyrophosphorylation of substrate proteins by the inositol pyrophosphate 5-InsP7
Source: Biosci Rep. 2024 Oct 4;44(10):BSR20240792. doi: 10.1042/BSR20240792 (PMC11461180; doi:10.1042/BSR20240792)
Supplement: Supplementary Tables S1-S3 [file BSR-2024-0792_supp.zip › BSR-2024-0792_suppst2.pdf]

Known interactor of IP6K1
Known substrate for IP7 mediated pyrophosphorylation
Casein kinase subunits
Pyrophosphorylated proteins identified by mass spectrometry
Cut-off FC-B score 1.4 used for GO term analysis by DAVID

		CRAPome Empirical fold change (FC) scores		Total peptide count			
PROTID	Known interactor / substrate (Reference)	IP6K1_FC_A	IP6K1_FC_B	GFP_1	GFP_2	IP6K1_1	IP6K1_2
IP6K1		34.65	31.82	3	0	178	194
USP9X		16.41	16.14	0	0	22	38
IP6K2	Huttlin EL (2021); PUBMED:33961781	13.21	11.23	0	0	23	16
UBE4A	Luck K (2020); PUBMED:32296183	10.28	10	0	0	14	21
AHCYL1		12.14	9.77	0	0	22	12
HADHA		8.61	7.56	0	1	20	16
CSNK2A1		6.06	5.55	0	0	9	8
AP3B1	Azevedo (2009); PUBMED: 19934039	5.9	5.3	0	0	9	7
PPM1G		5.7	5.26	0	0	3	21
RPS3A		4.64	4.63	0	0	4	12
RPS9		4.64	4.63	0	0	4	12
LRPPRC		4.13	3.98	0	6	11	50
HADHB		4.67	3.98	0	2	14	9
XRCC5		3.98	3.96	0	0	4	8
RPS19		3.8	3.63	0	0	2	12
DDB1	Rao F (2014); PUBMED:25349427	4.37	3.6	0	5	23	14
ILF2		3.74	3.54	0	1	3	18
MRPS22		3.64	3.51	0	0	2	11
TCOF1	Bhandari (2007); PUBMED 17873058,<br>Morgan (2024); PUBMED: 38664588	4.21	3.47	0	0	1	17
UBE2O		3.71	3.2	0	0	1	14
IRS4		3.41	2.93	0	0	5	2
POLD1		2.82	2.81	0	0	2	6
USP11		2.75	2.72	0	1	4	7
DDX21	Morgan (2024); PUBMED: 38664588	2.75	2.72	0	1	4	7
PRKDC		4.12	2.69	0	0	0	19
RPL6		3	2.68	0	0	4	2
RPL31		2.65	2.65	0	0	2	5
PTCD3		2.73	2.58	0	0	1	8
MRPS23		2.49	2.48	0	0	2	4
FBXO3		2.49	2.48	0	0	2	4
HNRNPH2		2.49	2.48	0	0	2	4
VIM	Luck K (2020); PUBMED:32296183	3.5	2.45	0	0	6	0
RCN2		2.58	2.41	0	0	3	2
PARP1		3.56	2.4	0	2	12	2
ILF3		2.79	2.37	0	1	1	15
TUBB6		2.4	2.33	0	0	1	6
CUL4B		2.4	2.33	0	0	1	6
MRPS9		2.33	2.3	0	0	2	3
RPS18		2.26	2.24	0	1	2	8
HSD17B10		2.97	2.22	0	0	0	12
DHX9		2.32	2.21	0	3	3	17
RPL23A		2.24	2.2	0	0	1	5
RPS25		2.24	2.2	0	0	1	5
SYNCRIP		2.32	2.17	0	2	2	14
AHCYL2		2.41	2.16	0	0	3	1
RPS7		2.27	2.11	0	1	4	3
HACD3		2.16	2.1	0	0	2	2

TPP1		2.16	2.1	0	0	2	2
RPS26		2.16	2.1	0	0	2	2
RPS8		2.09	2.05	0	1	3	4
CDK1		2.2	2.02	0	1	1	10
ANP32B		2.48	1.99	0	0	0	9
CUL1		2.48	1.99	0	0	0	9
MRPS27		2.48	1.99	0	0	0	9
DRG1		1.91	1.91	0	0	1	3
CCNB1		1.91	1.91	0	0	1	3
RPL13		1.91	1.91	0	0	1	3
SLC3A2		1.91	1.91	0	0	1	3
USP7		2.31	1.9	0	0	0	8
U2AF1L5		2.31	1.9	0	0	0	8
TMPO		2	1.88	0	0	2	1
SKP2		2	1.88	0	0	2	1
TECR		2	1.88	0	0	2	1
MATR3		2	1.88	0	0	2	1
UBR4		2	1.88	0	0	2	1
UBTF	Morgan (2024); PUBMED: 38664588	2.25	1.87	0	0	3	0
DBT		2.25	1.87	0	0	3	0
CHEK2		2.25	1.87	0	0	3	0
CSNK2A2		2.25	1.87	0	0	3	0
HNRNPC		2.25	1.87	0	0	3	0
HIST1H1C		2.25	1.87	0	0	3	0
DDX5		1.96	1.86	0	1	1	8
C1QBP		1.84	1.83	1	2	5	9
SET		2.15	1.82	0	0	0	7
RPS4X		1.86	1.82	0	2	2	9
PRMT1		2.15	1.82	0	0	0	7
RPS13		2.15	1.82	0	0	0	7
HNRNPR		2.09	1.8	0	2	1	14
HSPA5		2.03	1.79	3	3	14	11
XRCC6		1.77	1.75	0	2	2	8
RPS16		1.77	1.75	0	2	2	8
RPS10P5		1.75	1.74	0	0	1	2
RBMX		1.75	1.74	0	0	1	2
RPL22		1.75	1.74	0	0	1	2
MRPS26		1.75	1.74	0	0	1	2
MAGED1		1.75	1.74	0	0	1	2
RPS24		1.86	1.73	0	1	3	2
ANP32A		1.98	1.72	0	0	0	6
SHMT2		1.98	1.72	0	0	0	6
CSDE1		1.98	1.72	0	0	0	6
GNL3		1.98	1.72	0	0	0	6
HNRNPA1	Morgan (2024); PUBMED: 38664588	1.98	1.72	0	0	0	6
MRPS7		1.98	1.72	0	0	0	6
DIS3		1.98	1.72	0	0	0	6
PCCA		2.93	1.71	2	0	14	1
RPS17		1.73	1.68	0	1	1	6
RPS6		1.67	1.66	0	1	2	3
ANP32E		1.82	1.63	0	0	0	5
EIF2A		1.82	1.63	0	0	0	5
MCM2		1.82	1.63	0	0	0	5
DHX30		1.82	1.63	0	0	0	5
HNRNPAB		1.82	1.63	0	0	0	5
RTCB		1.82	1.63	0	0	0	5
MRPS10		1.82	1.63	0	0	0	5

MCM5		1.82	1.63	0	0	0	5
NOP56		1.83	1.63	0	0	2	0
MRPS2		1.82	1.63	0	0	0	5
HIST1H2BH		1.83	1.63	0	0	2	0
USP10		1.82	1.63	0	0	0	5
BOLA2		1.83	1.63	0	0	2	0
HDLBP		1.82	1.63	0	0	0	5
TRMT10C		1.82	1.63	0	0	0	5
G3BP1		1.82	1.63	0	0	0	5
RPS3		1.59	1.58	0	12	11	21
RAD18		1.58	1.56	0	0	1	1
EAF1		1.58	1.56	0	0	1	1
RPS26P11		1.58	1.56	0	0	1	1
HNRNPA3		1.58	1.56	0	0	1	1
SF3B3		1.58	1.56	0	0	1	1
MRPS16		1.58	1.56	0	0	1	1
HIST1H1A		1.58	1.56	0	0	1	1
PJA2		1.58	1.56	0	0	1	1
RPS29		1.58	1.56	0	0	1	1
RPL27		1.58	1.56	0	0	1	1
PTPMT1		1.58	1.56	0	0	1	1
GTF2I		1.66	1.52	0	0	0	4
SRPK1		1.66	1.52	0	0	0	4
PDE12		1.66	1.52	0	0	0	4
MRPL37		1.66	1.52	0	0	0	4
RANBP9		1.66	1.52	0	0	0	4
IGF2BP3		1.66	1.52	0	0	0	4
MRPS11		1.66	1.52	0	0	0	4
DNAJA2		1.66	1.52	0	0	0	4
SNRPF		1.66	1.52	0	0	0	4
MRPS35		1.66	1.52	0	0	0	4
MRPS5		1.66	1.52	0	0	0	4
NDUFS3		1.66	1.52	0	0	0	4
PUF60		1.66	1.52	0	0	0	4
SART3		1.66	1.52	0	0	0	4
NAP1L1	Morgan (2024); PUBMED: 38664588	1.49	1.48	0	1	1	4
RPL23		1.54	1.48	0	2	3	3
HNRNPU		1.56	1.47	0	6	3	18
HNRNPA2B1		1.53	1.46	0	3	4	4
AP1M1		1.49	1.41	0	0	0	3
SIRT1		1.49	1.41	0	0	0	3
MRPL15		1.49	1.41	0	0	0	3
CSNK2B		1.49	1.41	0	0	0	3
FLNA		1.49	1.41	0	0	0	3
ALDH3A2		1.49	1.41	0	0	0	3
LUC7L2		1.49	1.41	0	0	0	3
COPS4		1.49	1.41	0	0	0	3
DAP3		1.49	1.41	0	0	0	3
PFN1		1.49	1.41	0	0	0	3
RPLP2		1.49	1.41	0	0	0	3
SNRPA1		1.49	1.41	0	0	0	3
CDK5		1.49	1.41	0	0	0	3
UBAP2L		1.49	1.41	0	0	0	3
MRPL22		1.49	1.41	0	0	0	3
MRPS28		1.49	1.41	0	0	0	3
ENKD1		1.49	1.41	0	0	0	3
THUMPD3		1.49	1.41	0	0	0	3

PAPSS1		1.49	1.41	0	0	0	3
DDX3X		1.49	1.41	0	0	0	3
NXN		1.49	1.41	0	0	0	3
ATP2B4		1.49	1.41	0	0	0	3
KCTD12		1.49	1.41	0	0	0	3
EIF4G1	Shah (2021); PUBMED: 34841428	1.49	1.41	0	0	0	3
FEN1		1.49	1.41	0	0	0	3
DDX1		1.49	1.41	0	0	0	3
MRPL12		1.49	1.41	0	0	0	3
C14orf166		1.49	1.41	0	0	0	3
SNRPB		1.49	1.41	0	0	0	3
MRPS30		1.49	1.41	0	0	0	3
EEF1B2		1.49	1.41	0	0	0	3
RPS10		1.49	1.41	0	0	0	3
TSR1		1.49	1.41	0	0	0	3
DCAF1		1.49	1.41	0	0	0	3
YBX1		1.42	1.4	0	3	3	5
RPS15A		1.4	1.4	0	2	2	4
PABPC1		1.44	1.38	0	2	1	7
HNRNPD	Morgan (2024); PUBMED: 38664588	1.44	1.38	0	2	1	7
CCT6A		1.42	1.37	0	4	2	11
SLC25A3		1.41	1.37	1	2	4	5
CAMK2D		1.42	1.35	0	0	1	0
CAMK2G		1.42	1.35	0	0	1	0
RIF1		1.42	1.35	0	0	1	0
BANF1		1.42	1.35	0	0	1	0
ARFIP1		1.42	1.35	0	0	1	0
RPL12		1.42	1.35	0	0	1	0
tr|B4DX69|B4DX69_HUMAN		1.42	1.35	0	0	1	0
SRCAP		1.42	1.35	0	0	1	0
SLIRP		1.42	1.35	0	0	1	0
LMNA		1.42	1.35	0	0	1	0
DCAF8		1.42	1.35	0	0	1	0
DPM1		1.42	1.35	0	0	1	0
AP3S1		1.42	1.35	0	0	1	0
HIST2H2BD		1.42	1.35	0	0	1	0
RPL29		1.42	1.35	0	0	1	0
RPL8		1.42	1.35	0	0	1	0
PRSS3		1.42	1.35	0	0	1	0
GPC6		1.42	1.35	0	0	1	0
TRAK2		1.42	1.35	0	0	1	0
RPS2		1.33	1.33	0	4	3	7
PC		2.62	1.31	4	0	21	1
IQCB1		1.33	1.29	0	0	0	2
LARP1		1.33	1.29	0	0	0	2
POP1		1.33	1.29	0	0	0	2
CCT8		1.52	1.29	0	4	1	15
PSMD13		1.33	1.29	0	0	0	2
RPS4Y1		1.33	1.29	0	0	0	2
SCAF4		1.33	1.29	0	0	0	2
CHP1		1.33	1.29	0	0	0	2
EIF2S2		1.33	1.29	0	0	0	2
HEATR3		1.33	1.29	0	0	0	2
SLC25A10		1.33	1.29	0	0	0	2
EIF3A		1.33	1.29	0	0	0	2

EIF3B		1.33	1.29	0	0	0	2
MRPL39		1.33	1.29	0	0	0	2
NME2		1.33	1.29	0	0	0	2
MRPL41		1.33	1.29	0	0	0	2
MRPL44		1.33	1.29	0	0	0	2
ATP5O		1.33	1.29	0	0	0	2
DHX36		1.33	1.29	0	0	0	2
U2AF2		1.33	1.29	0	0	0	2
RPLP1		1.33	1.29	0	0	0	2
OXSR1		1.33	1.29	0	0	0	2
MYH9		1.33	1.29	0	0	0	2
ETFA		1.33	1.29	0	0	0	2
LARP4B		1.33	1.29	0	0	0	2
MRPS18B		1.33	1.29	0	0	0	2
RPL10L		1.33	1.29	0	0	0	2
MRPS17		1.33	1.29	0	0	0	2
FAM192A		1.33	1.29	0	0	0	2
ELAC2		1.33	1.29	0	0	0	2
AP1B1		1.33	1.29	0	0	0	2
CCT6B		1.33	1.29	0	0	0	2
LMAN1		1.33	1.29	0	0	0	2
AP2A2		1.33	1.29	0	0	0	2
NSUN2		1.33	1.29	0	0	0	2
TIMM44		1.33	1.29	0	0	0	2
PCK2		1.33	1.29	0	0	0	2
ZC3HAV1		1.33	1.29	0	0	0	2
CLCN7		1.33	1.29	0	0	0	2
ACOT8		1.33	1.29	0	0	0	2
RPL19		1.33	1.29	0	0	0	2
SHMT1		1.33	1.29	0	0	0	2
ARCN1		1.33	1.29	0	0	0	2
KPNA1		1.33	1.29	0	0	0	2
MRPL28		1.33	1.29	0	0	0	2
SRSF7		1.33	1.29	0	0	0	2
SRSF8		1.33	1.29	0	0	0	2
RBM39		1.33	1.29	0	0	0	2
FAM98B		1.33	1.29	0	0	0	2
BCCIP		1.33	1.29	0	0	0	2
GALK1		1.33	1.29	0	0	0	2
MRPS25		1.33	1.29	0	0	0	2
AKAP8L		1.33	1.29	0	0	0	2
FHL1		1.33	1.29	0	0	0	2
RPS28		1.33	1.29	0	0	0	2
ANKRD17		1.33	1.29	0	0	0	2
NAP1L4		1.33	1.29	0	0	0	2
NSF		1.33	1.29	0	0	0	2
NDUFS2		1.33	1.29	0	0	0	2
GLRX3		1.33	1.29	0	0	0	2
RPL5		1.33	1.29	0	0	0	2
UBXN4	Huttlin EL (2015); PUBMED:26186194	1.33	1.29	0	0	0	2
CTPS2		1.33	1.29	0	0	0	2
HTT		1.33	1.29	0	0	0	2
RPS11		1.33	1.29	0	0	0	2
MAP7D1		1.33	1.29	0	0	0	2
RPL35		1.33	1.29	0	0	0	2
MCCC2		2.16	1.28	2	0	10	0
DDX17		1.26	1.24	0	2	1	5
VPS35		1.43	1.24	0	1	0	6
NDUFS1		1.22	1.22	0	3	2	5

TCP1	1.22	1.19	0	7	3	14
RPL24	1.21	1.18	0	2	2	2
HNRNPM	1.25	1.18	2	7	9	10
NONO	1.32	1.17	0	1	2	0
QARS	1.31	1.17	0	1	0	5
DDX3Y	1.31	1.17	0	1	0	5
ATP5C1	1.31	1.17	0	1	0	5
ALDH18A1	1.31	1.17	0	1	0	5
PDIA6	1.31	1.17	0	1	0	5
DARS	1.48	1.16	0	2	0	10
KHSRP	1.16	1.15	0	0	0	1
NCBP1	1.16	1.15	0	0	0	1
MRPL54	1.16	1.15	0	0	0	1
MRPL50	1.16	1.15	0	0	0	1
MRPL58	1.16	1.15	0	0	0	1
LARP7	1.16	1.15	0	0	0	1
NUP93	1.16	1.15	0	0	0	1
OSBPL8	1.16	1.15	0	0	0	1
POP7	1.16	1.15	0	0	0	1
NOP9	1.16	1.15	0	0	0	1
QSOX2	1.16	1.15	0	0	0	1
NCAPD2	1.16	1.15	0	0	0	1
PYM1	1.16	1.15	0	0	0	1
ANAPC1	1.16	1.15	0	0	0	1
GART	1.16	1.15	0	0	0	1
RPS6KA2	1.16	1.15	0	0	0	1
SNRPB2	1.16	1.15	0	0	0	1
DNAJC9	1.16	1.15	0	0	0	1
PSMD14	1.16	1.15	0	0	0	1
MYBBP1A	1.16	1.15	0	0	0	1
ZNF428	1.16	1.15	0	0	0	1
POLR2C	1.16	1.15	0	0	0	1
POLR2B	1.16	1.15	0	0	0	1
DCTN4	1.16	1.15	0	0	0	1
MCAT	1.16	1.15	0	0	0	1
CDC45	1.16	1.15	0	0	0	1
TROVE2	1.16	1.15	0	0	0	1
PRKD1	1.16	1.15	0	0	0	1
PABPN1	1.16	1.15	0	0	0	1
CDC42	1.16	1.15	0	0	0	1
LARP1B	1.16	1.15	0	0	0	1
EEF1A2	1.16	1.15	0	0	0	1
PSMD3	1.16	1.15	0	0	0	1
CPSF6	1.16	1.15	0	0	0	1
ILVBL	1.16	1.15	0	0	0	1
ARIH1	1.16	1.15	0	0	0	1
NOLC1	1.16	1.15	0	0	0	1
Bhandari (2007); Pubmed 17873058						
Morgan (2024); PUBMED: 38664588						
LRRC1	1.16	1.15	0	0	0	1
NAF1	1.16	1.15	0	0	0	1
LBR	1.16	1.15	0	0	0	1
FAR1	1.16	1.15	0	0	0	1
ACTR1A	1.16	1.15	0	0	0	1
WDR77	1.16	1.15	0	0	0	1
TRMT112	1.16	1.15	0	0	0	1
ARAF	1.16	1.15	0	0	0	1
EIF3K	1.16	1.15	0	0	0	1

EIF3L		1.16	1.15	0	0	0	1
EIF3D		1.16	1.15	0	0	0	1
LPCAT1		1.16	1.15	0	0	0	1
NTPCR		1.16	1.15	0	0	0	1
PABPC4		1.16	1.15	0	0	0	1
H1FX		1.16	1.15	0	0	0	1
MRPL40		1.16	1.15	0	0	0	1
MRPL46		1.16	1.15	0	0	0	1
MRPL49		1.16	1.15	0	0	0	1
NOA1		1.16	1.15	0	0	0	1
RPL27A		1.16	1.15	0	0	0	1
YTHDF2		1.16	1.15	0	0	0	1
CCAR2		1.16	1.15	0	0	0	1
AP2B1		1.16	1.15	0	0	0	1
SF3A3		1.16	1.15	0	0	0	1
KPNA3		1.16	1.15	0	0	0	1
MTHFD2		1.16	1.15	0	0	0	1
HDAC2	Morgan (2024); PUBMED: 38664588	1.16	1.15	0	0	0	1
RFC5		1.16	1.15	0	0	0	1
COASY		1.16	1.15	0	0	0	1
FASTKD2		1.16	1.15	0	0	0	1
FIGNL1		1.16	1.15	0	0	0	1
LLGL1		1.16	1.15	0	0	0	1
SKIV2L2		1.16	1.15	0	0	0	1
MTERF3		1.16	1.15	0	0	0	1
MRPS15		1.16	1.15	0	0	0	1
MRPS14		1.16	1.15	0	0	0	1
NCLN		1.16	1.15	0	0	0	1
YWHAB		1.16	1.15	0	0	0	1
PRKCI		1.16	1.15	0	0	0	1
COPA		1.16	1.15	0	0	0	1
IARS2		1.16	1.15	0	0	0	1
LSM3		1.16	1.15	0	0	0	1
SLC16A1	Morgan (2024); PUBMED: 38664588	1.16	1.15	0	0	0	1
GSTP1		1.16	1.15	0	0	0	1
SSR3		1.16	1.15	0	0	0	1
CAPRIN1		1.16	1.15	0	0	0	1
AHSA1		1.16	1.15	0	0	0	1
CUL5		1.16	1.15	0	0	0	1
CUL2		1.16	1.15	0	0	0	1
CUL3		1.16	1.15	0	0	0	1
SLC25A1		1.16	1.15	0	0	0	1
SLC25A4		1.16	1.15	0	0	0	1
TMEM109		1.16	1.15	0	0	0	1
RPL10		1.16	1.15	0	0	0	1
DHCR7		1.16	1.15	0	0	0	1
RBM42		1.16	1.15	0	0	0	1
SNRPD2		1.16	1.15	0	0	0	1
SNRPD1		1.16	1.15	0	0	0	1
KPNA6		1.16	1.15	0	0	0	1
KPNA5		1.16	1.15	0	0	0	1
LYAR		1.16	1.15	0	0	0	1
RTCA		1.16	1.15	0	0	0	1
MRPL20		1.16	1.15	0	0	0	1
MED23		1.16	1.15	0	0	0	1
IGHA1		1.16	1.15	0	0	0	1
SRSF5	Morgan (2024); PUBMED: 38664588	1.16	1.15	0	0	0	1
SRSF6	Morgan (2024); PUBMED: 38664588	1.16	1.15	0	0	0	1
SRSF3		1.16	1.15	0	0	0	1

SRSF2	Morgan (2024); PUBMED: 38664588	1.16	1.15	0	0	0	1
USP47		1.16	1.15	0	0	0	1
CISD2		1.16	1.15	0	0	0	1
CLNS1A		1.16	1.15	0	0	0	1
UMPS		1.16	1.15	0	0	0	1
GID8		1.16	1.15	0	0	0	1
RSL1D1		1.16	1.15	0	0	0	1
MRPL3		1.16	1.15	0	0	0	1
MRPL1		1.16	1.15	0	0	0	1
MRPL9		1.16	1.15	0	0	0	1
ACAD9		1.16	1.15	0	0	0	1
MOGS		1.16	1.15	0	0	0	1
MRPS21		1.16	1.15	0	0	0	1
TOMM20		1.16	1.15	0	0	0	1
PPP2R5D		1.16	1.15	0	0	0	1
GPALPP1		1.16	1.15	0	0	0	1
SEC23A		1.16	1.15	0	0	0	1
DHX57		1.16	1.15	0	0	0	1
PSMA8		1.16	1.15	0	0	0	1
RPS6KA3		1.16	1.15	0	0	0	1
HTATSF1	Morgan (2024); PUBMED: 38664588	1.16	1.15	0	0	0	1
EIF2AK2		1.16	1.15	0	0	0	1
ATL2		1.16	1.15	0	0	0	1
PPIB		1.16	1.15	0	0	0	1
DCAF16		1.16	1.15	0	0	0	1
PPP2R1B		1.16	1.15	0	0	0	1
AIMP1		1.16	1.15	0	0	0	1
TMEM33		1.16	1.15	0	0	0	1
COPG2		1.16	1.15	0	0	0	1
CCDC47		1.16	1.15	0	0	0	1
ZCCHC6		1.16	1.15	0	0	0	1
ZNHIT6		1.16	1.15	0	0	0	1
MCM4		1.16	1.15	0	0	0	1
GTPBP1		1.16	1.15	0	0	0	1
EIF4G2		1.16	1.15	0	0	0	1
AHCY		1.16	1.15	0	0	0	1
PKMYT1		1.16	1.15	0	0	0	1
PYGB		1.16	1.15	0	0	0	1
SOD1		1.16	1.15	0	0	0	1
P4HB		1.16	1.15	0	0	0	1
HK1		1.16	1.15	0	0	0	1
UBR5		1.16	1.15	0	0	0	1
EXD2		1.16	1.15	0	0	0	1
MAGED2		1.16	1.15	0	0	0	1
NRDC		1.16	1.15	0	0	0	1
TRIM33		1.16	1.15	0	0	0	1
RPL4		1.16	1.15	0	0	0	1
TBRG4		1.16	1.15	0	0	0	1
CHD4		1.16	1.15	0	0	0	1
CKAP4		1.16	1.15	0	0	0	1
RCN1		1.16	1.15	0	0	0	1
MRPL17		1.16	1.15	0	0	0	1
MRPL18		1.16	1.15	0	0	0	1
GYS1		1.16	1.15	0	0	0	1
POLR2A		1.16	1.15	0	0	0	1
SLC25A40		1.16	1.15	0	0	0	1
STAT1		1.16	1.15	0	0	0	1
IPO4		1.16	1.15	0	0	0	1
NRBP1		1.16	1.15	0	0	0	1

FAM120A		1.16	1.15	0	0	0	1
MAPK10		1.16	1.15	0	0	0	1
TNPO3		1.16	1.15	0	0	0	1
POLDIP2		1.16	1.15	0	0	0	1
HNRNPUL2		1.16	1.15	0	0	0	1
METTL3		1.16	1.15	0	0	0	1
HP1BP3		1.16	1.15	0	0	0	1
RPS14		1.15	1.15	0	5	3	7
NDUFA4		1.16	1.15	0	0	0	1
IKBKAP		1.16	1.15	0	0	0	1
IDH3A		1.16	1.15	0	0	0	1
IDH3B		1.16	1.15	0	0	0	1
CAND2		1.16	1.15	0	0	0	1
EXOSC7	Rual JF (2005); PUBMED: 16189514	1.16	1.15	0	0	0	1
PLS3		1.16	1.15	0	0	0	1
TMEM165		1.16	1.15	0	0	0	1
ARMC8		1.16	1.15	0	0	0	1
LRRC59		1.16	1.15	0	0	0	1
SNRPE		1.16	1.15	0	0	0	1
IMMT		1.16	1.15	0	0	0	1
SERPINH1		1.16	1.15	0	0	0	1
CCT5		1.39	1.12	0	2	0	9
DNAJA1		1.14	1.12	0	1	1	1
EMD		1.14	1.12	0	1	1	1
RPS23		1.14	1.12	0	1	1	1
RPL7		1.14	1.12	0	1	1	1
ALDH1B1		1.14	1.12	0	1	1	1
FKBP8		1.19	1.09	0	1	0	4
CCT2		1.14	1.09	0	6	2	12
MSH2		1.19	1.09	0	1	0	4
RPL17		1.19	1.09	0	1	0	4
ZYX		1.19	1.09	0	1	0	4
SERBP1	Morgan (2024); PUBMED: 38664588	1.19	1.09	0	1	0	4
ATP2A1		1.12	1.06	0	2	2	1
RPL11	Luck K (2020); PUBMED :32296183	1.12	1.06	0	2	2	1
HNRNPA1L2		1.07	1.06	0	3	2	3
TRIM72		1.26	1.05	0	2	3	0
NCL		1.06	1.03	0	11	4	18
SLC25A13		1.21	1.02	0	2	0	7
IGF2BP1		1.21	1.02	0	2	0	7
GAPVD1		1.07	1.01	0	1	0	3
SFXN1		1.07	1.01	0	1	0	3
TUBA3C		1.07	1.01	0	1	0	3
EIF3F		1.07	1.01	0	1	0	3
PFKP		1.07	1.01	0	1	0	3
RPS20		1.07	1.01	0	1	0	3
ALDOA		1.07	1.01	0	1	0	3
KARS		1.07	1.01	0	1	0	3
CAD		1.01	1	1	2	2	4
TRIM28		1.12	1	0	12	3	25
SKP1		0.98	0.98	0	2	1	2
HSPBP1		1.02	0.97	0	1	1	0
HNRNPCL2		1.08	0.96	1	0	2	0
RUVBL1		0.97	0.96	0	8	4	8
MRPS31		1.07	0.96	1	0	0	5
AIFM1		0.96	0.95	0	3	1	4

GCN1	1.22	0.95	0	3	0	10
HIST1H2BB	1.54	0.94	3	0	9	0
LARP4	0.96	0.93	0	1	0	2
STIP1	0.96	0.93	0	1	0	2
XPO5	0.96	0.93	0	1	0	2
ACAT1	0.96	0.93	0	1	0	2
PSMC6	0.96	0.93	0	1	0	2
TRIP13	0.96	0.93	0	1	0	2
SLC25A11	0.96	0.93	0	1	0	2
NSDHL	0.96	0.93	0	1	0	2
RAB1A	0.96	0.93	0	1	0	2
ENO3	0.96	0.93	0	1	0	2
SSR1	0.96	0.93	0	1	0	2
GPI	0.96	0.93	0	1	0	2
TUBB3	0.96	0.93	0	1	0	2
RPSA	0.96	0.93	0	1	0	2
DHX15	0.96	0.93	0	1	0	2
GCN1L1	0.96	0.93	0	1	0	2
STOML2	0.96	0.93	0	1	0	2
DDOST	0.96	0.93	0	1	0	2
RPS27	0.93	0.92	1	0	1	1
RACK1	1.22	0.9	0	4	0	13
FASN	1.26	0.9	0	10	1	29
HIST1H2AH	1.12	0.89	4	0	7	2
ATP2A2	0.88	0.88	0	3	1	3
TIMM50	0.92	0.87	0	3	2	1
HSPA4	0.87	0.86	0	4	1	5
HSPA9	0.93	0.86	8	30	27	29
YBX3	0.93	0.85	0	2	0	4
EIF4A1	0.93	0.85	0	2	0	4
RPN1	0.93	0.85	0	2	0	4
SSB	0.93	0.85	0	2	0	4
ACTG1	0.87	0.85	0	6	3	4
CTPS1	0.93	0.85	0	2	0	4
RPLP0P6	0.93	0.85	0	2	0	4
GRWD1	0.84	0.83	0	1	0	1
PSMD11	0.84	0.83	0	1	0	1
AARSD1	0.84	0.83	0	1	0	1
IMPDH2	0.84	0.83	0	1	0	1
ATP5J2	0.84	0.83	0	1	0	1
TSFM	0.84	0.83	0	1	0	1
ARIH2	0.84	0.83	0	1	0	1
SACM1L	0.84	0.83	0	1	0	1
YWHAQ	0.84	0.83	0	1	0	1
OLA1	0.84	0.83	0	1	0	1
DNAAF5	0.84	0.83	0	1	0	1
TBC1D9B	0.84	0.83	0	1	0	1
FARSA	0.84	0.83	0	1	0	1
EIF5	0.84	0.83	0	1	0	1
PPP6R3	0.84	0.83	0	1	0	1
FLNB	0.84	0.83	0	1	0	1
PPP2CA	0.84	0.83	0	1	0	1
NUP205	0.84	0.83	0	1	0	1
HSP90AA2P	0.84	0.83	0	1	0	1
NACA	0.84	0.83	0	1	0	1
TUBAL3	0.84	0.83	0	1	0	1

UBA52		0.88	0.81	3	3	5	4
HNRNPK		1.03	0.81	0	4	0	10
EWSR1		0.8	0.8	0	3	1	2
GCC2		0.84	0.8	1	0	1	0
TUBB2B		0.81	0.8	1	7	3	8
XPOT		0.84	0.79	0	2	0	3
HSP90AB4P		0.84	0.79	0	2	0	3
VCP	Zuzow N (2018); PUBMED:29540532	0.84	0.79	0	2	0	3
CUL4A	Rao F (2014); PUBMED: 25349427	0.84	0.79	0	2	0	3
MDH2		0.84	0.79	0	2	0	3
MCM6		0.84	0.79	0	2	0	3
HIST2H3A		0.94	0.78	2	0	3	0
UQCRC2		0.97	0.78	0	4	0	9
SLC25A5		0.78	0.78	2	2	2	4
RPLP0		0.84	0.75	0	3	0	5
SLC25A6		0.75	0.75	9	7	9	17
NPM1	Morgan (2024); PUBMED: 38664588	0.79	0.74	1	4	3	2
CCT4		0.95	0.73	0	5	0	11
MRI1		0.75	0.72	0	2	0	2
STRAP		0.75	0.72	0	2	0	2
FANCI		0.75	0.72	0	2	0	2
YWHAH		0.75	0.72	0	2	0	2
PRMT5		0.75	0.72	0	2	0	2
COPG1		0.75	0.72	0	2	0	2
PCNA		0.75	0.72	0	2	0	2
MCM7		0.75	0.72	0	2	0	2
ST13		0.75	0.72	0	2	0	2
IPO9		0.75	0.72	0	2	0	2
IPO5		0.75	0.72	0	2	0	2
MARS		0.84	0.71	0	4	0	7
RARS		0.84	0.71	0	4	0	7
MCM3		0.84	0.71	0	4	0	7
EIF4A2	Shah A (2021), PUBMED: 34841428	0.76	0.7	0	3	0	4
PSMC2		0.76	0.7	0	3	0	4
UBA1		0.72	0.7	0	6	1	6
NAMPT		0.76	0.7	0	3	0	4
LDHA		0.76	0.7	0	3	0	4
EEF2		0.69	0.68	1	4	1	5
STUB1		0.69	0.68	1	0	0	1
LYZ		0.69	0.68	1	0	0	1
HNRNPF		0.68	0.68	0	4	1	2
ASNS		0.78	0.67	0	4	0	6
CAND1		0.78	0.67	0	4	0	6
AARS2		0.78	0.67	0	4	0	6
KPNB1		0.67	0.66	0	6	1	5
CCT7		0.78	0.65	0	5	0	8
PAICS		0.65	0.65	0	2	0	1
PSMD2		0.69	0.65	0	3	0	3
CBSL		0.65	0.65	0	2	0	1
FBXO22		0.65	0.65	0	2	0	1
FBXO21		0.65	0.65	0	2	0	1
YWHAZ		0.65	0.65	0	2	0	1
TFRC		0.65	0.65	0	2	0	1
PGK1		0.65	0.65	0	2	0	1
PSMA7		0.65	0.65	0	2	0	1
PPP2R1A		0.65	0.65	0	2	0	1
UNC45A		0.69	0.65	0	3	0	3
MCCC1		1.05	0.62	7	2	14	1

PRDX4		0.64	0.62	1	1	0	2
KPNA2		0.73	0.62	0	5	0	7
EEF1A1		0.63	0.61	2	8	2	10
ATP5A1		0.62	0.61	0	14	4	8
LARS		0.8	0.6	0	7	0	12
AGK		0.61	0.59	0	3	0	2
PSMC5		0.61	0.59	0	3	0	2
SLC1A5		0.59	0.59	0	5	1	2
YWHAG		0.61	0.59	0	3	0	2
CANX	Liu X (2018); PUBMED:29568061	0.61	0.59	0	3	0	2
	Morgan (2024); PUBMED: 38664588						
EEF1G		0.65	0.59	0	4	0	4
ACTA2		0.6	0.58	6	13	8	11
FABP5		0.59	0.57	2	0	1	0
PIP		0.59	0.57	2	0	1	0
CDSN		0.57	0.56	2	1	1	1
YWHAE		0.61	0.56	1	15	2	17
ACLY		0.58	0.55	0	4	0	3
ENO1		0.62	0.55	0	5	0	5
TUFM		0.58	0.55	0	4	0	3
HSPB1		0.55	0.55	2	2	1	2
HSP90AA1	Morgan (2024); PUBMED: 38664588	0.58	0.55	2	12	2	14
PCMT1		0.71	0.55	0	7	0	10
HIST1H4A		0.95	0.54	7	0	11	0
RPL7A		0.54	0.53	0	3	0	1
PSMC3		0.54	0.53	0	3	0	1
OTUB1		0.54	0.53	0	3	0	1
ATP1A1		0.59	0.52	0	6	0	6
MTHFD1		0.59	0.52	0	6	0	6
TUBB		0.56	0.52	4	11	6	6
HSPA8		0.53	0.51	18	16	15	20
PCCB		0.74	0.51	7	2	9	1
ACACA		1.03	0.5	27	15	58	7
CSE1L		0.52	0.5	0	4	0	2
RPN2		0.52	0.49	1	3	0	3
PRDX2		0.51	0.49	4	4	3	3
RUVBL2		0.5	0.48	0	16	2	11
PRDX1		0.5	0.48	2	5	2	2
HSPA2		0.48	0.47	5	5	3	5
PHGDH		0.5	0.46	0	6	0	4
HSPA1L		0.46	0.46	10	11	6	13
TRAP1		0.45	0.45	0	4	0	1
LDHB		0.49	0.44	0	7	0	5
TUBB4B		0.43	0.43	15	49	17	31
TUBA1C		0.46	0.43	20	25	17	20
PHB		0.45	0.42	0	6	0	3
HSP90B1		0.51	0.41	0	10	0	9
CCT3		0.5	0.39	0	11	0	10
IARS		0.47	0.39	0	10	0	8
ANXA2		0.41	0.39	3	1	1	0
CLTC		0.39	0.39	0	5	0	1
RNH1		0.42	0.39	0	15	1	9
XPO1		0.4	0.38	0	7	0	3
HSPA1B		0.39	0.38	11	8	5	9
TUBA4A		0.37	0.36	2	2	0	1
CKB		0.4	0.36	0	9	0	5
ATP5B		0.46	0.34	0	14	0	12
HSPD1		0.35	0.32	2	16	1	10

EPRS		0.41	0.31	0	15	0	11
HSP90AB2P		0.32	0.31	0	8	0	2
PKM		0.44	0.3	1	17	0	16
HSP90AB3P		0.3	0.28	2	16	1	7
HSP90AB1	Morgan (2024); PUBMED: 38664588	0.27	0.27	2	15	1	5
GFP_Aequorea		0.08	0.03	340	0	45	0
