# Supplementary material for: Interaction with IP6K1 supports pyrophosphorylation of substrate proteins by the inositol pyrophosphate 5-InsP7
Source: Biosci Rep. 2024 Oct 4;44(10):BSR20240792. doi: 10.1042/BSR20240792 (PMC11461180; doi:10.1042/BSR20240792)
Supplement: Supplementary Tables S1-S3 [file BSR-2024-0792_supp.zip › BSR-2024-0792_suppst3.pdf]

BP - Biological Process; CC - Cellular Component;
MF - Molecular Function
GO term used in Fig 1B

Annotation Cluster 1 — Enrichment Score: 33.0206881463327

Category	Term	Count	%	PValue	Genes	List Total	Pop Hits	Pop Total	Fold Enrichment	Bonferroni	Benjamini	FDR
GOTERM_MF_DIRECT	GO:0003735~structural constituent of ribosome	43	24.71264368	6.28E-47	MRPS16, MRPS35, RPL31, MRPS11, MRPS30, MRPL15, MRPL37, MRPL12, RPL6, RPS4X, RPS17, RPS15A, RPS16, RPS19, RPS18, RPS3, RPLP2, RPL13, RPS10, RPS13, RPS9, RPS7, RPS8, RPL23, MRPS22, MRPS23, RPL22, RPS6, MRPS2, RPL23A, RPS3A, MRPS7, MRPS5, MRPL22, RPS26, RPS25, MRPS9, RPS10P5, RPS29, RPS26P11, RPL27, RPS24, DAP3	173	189	18908	24.86601217	2.46E-44	1.23E-44	1.14E-44
GOTERM_CC_DIRECT	GO:0005840~ribosome	38	21.83908046	1.56E-41	MRPS16, RPL31, MRPS11, MRPS30, MRPL37, RPL6, RPS4X, RPS17, RPS15A, RPS16, RPS19, RPS18, RPS3, RPL13, RPS10, RPS13, MRPS28, PTCD3, RPS9, MRPS27, RPS7, RPS8, RPL23, MRPS22, MRPS23, RPL22, RPS6, MRPS2, RPL23A, RPS3A, MRPS7, RPS26, RPS25, MRPS9, RPS29, RPL27, RPS24, DAP3	174	176	20580	25.53683386	4.34E-39	4.34E-39	3.65E-39
GOTERM_BP_DIRECT	GO:0006412~translation	41	23.56321839	3.01E-41	MRPS16, RPL31, MRPS11, MRPS30, MRPL15, MRPL37, MRPL12, RPL6, RPS4X, RPS17, RPS15A, RPS16, RPS19, RPS18, RPS3, RPLP2, RPL13, IGF2BP3, RPS10, RPS13, RPS9, RPS7, RPS8, RPL23, MRPS23, RPL22, RPS6, MRPS2, RPL23A, RPS3A, MRPS7, MRPS5, MRPL22, RPS26, RPS25, MRPS9, RPS29, RPS26P11, RPL27, RPS24, EIF4G1	171	223	19333	20.7865366	3.38E-38	3.38E-38	3.29E-38
GOTERM_BP_DIRECT	GO:0002181~cytoplasmic translation	27	15.51724138	8.60E-33	RPL31, RPL6, RPS4X, RPS17, RPS15A, RPS16, RPS19, RPS18, RPS3, RPLP2, RPL13, RPS10, RPS13, RPS9, RPS7, RPS8, RPL23, RPL22, RPS6, RPL23A, RPS3A, RPS26, RPS25, RPS29, DRG1, RPL27, RPS24	171	90	19333	33.91754386	9.67E-30	4.83E-30	4.70E-30
GOTERM_CC_DIRECT	GO:0022627~cytosolic small ribosomal subunit	22	12.64367816	4.29E-32	RPS9, RPS7, RPS8, MRPS11, RPS6, RPS3A, RPS4X, RPS26, RPS25, RPS17, RPS10P5, RPS15A, RPS16, RPS19, RPS18, RPS29, RPS3, RPS26P11, RPS10, RPS24, RPS13, EIF2A	174	46	20580	56.56671664	1.19E-29	5.97E-30	5.02E-30
GOTERM_CC_DIRECT	GO:0022626~cytosolic ribosome	23	13.2183908	3.73E-28	RPS9, RPS7, RPL31, RPS8, RPL23, RPL22, RPS6, RPL23A, RPS3A, RPL6, RPS4X, RPS25, RPS17, RPS15A, RPS16, RPS19, RPS18, RPS3, RPL13, RPL27, RPS10, RPS24, RPS13	174	77	20580	35.32915361	1.04E-25	3.46E-26	2.91E-26
GOTERM_CC_DIRECT	GO:0005925~focal adhesion	25	14.36781609	1.76E-13	RPL31, HACD3, RPL6, RPS4X, RPS17, RPS16, RPS19, RPS18, G3BP1, RPS3, FLNA, RPLP2, RPS10, RPS13, RPS9, HSPA5, RPS7, RPS8, RPL23, RPL22, RPS3A, RPS29, RPL27, VIM, PFN1	174	425	20580	6.957403651	4.90E-11	4.46E-12	3.75E-12

Annotation Cluster 2 — Enrichment Score: 15.946728141284252

Category	Term	Count	%	PValue	Genes	List Total	Pop Hits	Pop Total	Fold Enrichment	Bonferroni	Benjamini	FDR
GOTERM_CC_DIRECT	GO:0005763~mitochondrial small ribosomal subunit	15	8.620689655	3.64E-21	MRPS28, PTCD3, MRPS26, MRPS27, MRPS16, MRPS35, MRPS11, MRPS22, MRPS23, MRPS10, MRPS2, MRPS7, MRPS5, MRPS9, DAP3	174	33	20580	53.76175549	1.01E-18	1.69E-19	1.42E-19
GOTERM_BP_DIRECT	GO:0032543~mitochondrial translation	20	11.49425287	9.45E-21	MRPS28, PTCD3, MRPS26, MRPS27, MRPS16, MRPS35, MRPS11, MRPS22, MRPS23, MRPS10, MRPS2, MRPL15, MRPS30, MRPL37, MRPL12, MRPS7, MRPS5, MRPL22, MRPS9, DAP3	171	96	19333	23.5538499	1.06E-17	3.54E-18	3.44E-18
GOTERM_CC_DIRECT	GO:0005743~mitochondrial inner membrane	26	14.94252874	3.09E-13	MRPS16, MRPS35, SHMT2, MRPS11, MRPS10, MRPS30, MRPL15, MRPL37, MRPL12, PTPMT1, RPS3, MRPS28, PTCD3, MRPS26, MRPS27, MRPS22, MRPS23, MRPS2, MRPS7, MRPS5, MRPL22, HADHB, HADHA, MRPS9, NDUFS3, DAP3	174	479	20580	6.419984162	8.59E-11	7.16E-12	6.03E-12
GOTERM_CC_DIRECT	GO:0005739~mitochondrion	41	23.56321839	1.54E-11	FEN1, MRPS16, MRPS35, SHMT2, MRPS11, DDX1, MRPS10, DDX21, MRPS30, MRPL15, MRPL37, MRPL12, HSD17B10, DHX30, C1QBP, PTPMT1, DBT, MRPS28, PTCD3, MRPS26, MRPS27, TRMT10C, PARP1, HSPA5, MRPS22, MRPS23, MRPS2, AP3B1, SIRT1, MRPS5, LRPPRC, MRPL22, HADHB, HADHA, MRPS9, ILF3, PCCA, PDE12, CDK1, NDUFS3, DAP3	174	1451	20580	3.342047102	4.28E-09	3.05E-10	2.57E-10

Annotation Cluster 3 — Enrichment Score: 6.744373421982328

Category	Term	Count	%	PValue	Genes	List Total	Pop Hits	Pop Total	Fold Enrichment	Bonferroni	Benjamini	FDR

GOTERM_CC_DIRECT	GO:0071013~catalytic step 2 spliceosome	13	7.471264368	1.15E-11	DDX5, HNRNPA3, SF3B3, HNRNPU, HNRNPR, SYNCRIP, HNRNPA2B1, SNRPA1, SNRPF, HNRNPC, HNRNPA1, RBMX, SNRPB	174	89	20580	17.27624952	3.19E-09	2.46E-10	2.07E-10
GOTERM_BP_DIRECT	GO:0000398~mRNA splicing, via spliceosome	14	8.045977011	2.68E-08	DDX5, HNRNPA3, SF3B3, HNRNPU, HNRNPR, SYNCRIP, SART3, HNRNPA2B1, SNRPA1, SNRPF, HNRNPC, HNRNPA1, RBMX, SNRPB	171	201	19333	7.874719967	3.01E-05	5.01E-06	4.87E-06
GOTERM_CC_DIRECT	GO:0005681~spliceosomal complex	10	5.747126437	1.06E-06	DDX5, SF3B3, HNRNPA2B1, SNRPA1, HNRNPR, SNRPF, HNRNPC, HNRNPA1, RBMX, SNRPB	174	124	20580	9.538375973	2.93E-04	1.28E-05	1.07E-05
GOTERM_BP_DIRECT	GO:0008380~RNA splicing	12	6.896551724	1.63E-06	SYNCRIP, SF3B3, PUF60, PRMT1, C1QBP, SNRPA1, YBX1, SNRPF, HNRNPC, HNRNPA1, SNRPB, SRPK1	171	200	19333	6.783508772	0.001825527	1.66E-04	1.62E-04
GOTERM_MF_DIRECT	GO:0003730~mRNA 3'-UTR binding	10	5.747126437	3.60E-04	DDX5, HNRNPA3, RPS7, HNRNPA2B1, HNRNPU, IGF2BP3, HNRNPR, HNRNPC, HNRNPA1, LRPPRC	173	240	18908	4.553949904	0.131662455	0.008324084	0.007688657

Annotation Cluster 4	Enrichment Score: 5.655127050865156											
Category	Term	Count	%	PValue	Genes	List Total	Pop Hits	Pop Total	Fold Enrichment	Bonferroni	Benjamini	FDR
GOTERM_BP_DIRECT	GO:0070934~CRD-mediated mRNA stabilization	6	3.448275862	2.19E-08	SYNCRIP, DHX9, CSDE1, HNRNPU, IGF2BP3, YBX1	171	11	19333	61.66826156	2.47E-05	4.93E-06	4.80E-06
GOTERM_CC_DIRECT	GO:0070937~CRD-mediated mRNA stability complex	5	2.873563218	7.14E-08	SYNCRIP, DHX9, CSDE1, HNRNPU, YBX1	174	6	20580	98.56321839	1.99E-05	1.10E-06	9.28E-07
GOTERM_BP_DIRECT	GO:1900152~negative regulation of nuclear-transcribed mRNA catabolic process, deadenylation-dependent decay	5	2.873563218	7.03E-07	SYNCRIP, DHX9, CSDE1, HNRNPU, YBX1	171	9	19333	62.81026641	7.89E-04	7.89E-05	7.68E-05
GOTERM_BP_DIRECT	GO:2000767~positive regulation of cytoplasmic translation	5	2.873563218	9.67E-06	SYNCRIP, DHX9, CSDE1, HNRNPU, YBX1	171	16	19333	35.33077485	0.010811851	9.05E-04	8.81E-04
GOTERM_BP_DIRECT	GO:0001649~osteoblast differentiation	6	3.448275862	0.00498	SYNCRIP, DHX9, DDX21, HNRNPU, HNRNPC, RBMX	171	125	19333	5.426807018	0.996342763	0.151137452	0.147099942

Annotation Cluster 5	Enrichment Score: 3.873840293839095											
Category	Term	Count	%	PValue	Genes	List Total	Pop Hits	Pop Total	Fold Enrichment	Bonferroni	Benjamini	FDR
GOTERM_MF_DIRECT	GO:0003684~damaged DNA binding	9	5.172413793	1.85E-07	DDB1, XRCC6, FEN1, PARP1, XRCC5, POLD1, RPS3, RAD18, CUL4B	173	68	18908	14.46548793	7.25E-05	1.04E-05	9.60E-06
GOTERM_BP_DIRECT	GO:0006974~cellular response to DNA damage stimulus	10	5.747126437	8.36E-04	DDB1, PARP1, XRCC5, PRKDC, USP10, CHEK2, RPS3, SIRT1, RAD18, CUL4B	171	279	19333	4.052275252	0.60959239	0.039138919	0.038093356
GOTERM_BP_DIRECT	GO:0006281~DNA repair	8	4.597701149	0.015444	DDB1, FEN1, PARP1, USP10, POLD1, CDK1, RPS3, RAD18	171	294	19333	3.076421212	0.999999975	0.340080136	0.330995181

Annotation Cluster 6	Enrichment Score: 3.7474208187642732											
Category	Term	Count	%	PValue	Genes	List Total	Pop Hits	Pop Total	Fold Enrichment	Bonferroni	Benjamini	FDR
GOTERM_BP_DIRECT	GO:1903608~protein localization to cytoplasmic stress granule	4	2.298850575	2.28E-05	DDX3X, DHX9, DDX1, YBX1	171	7	19333	64.60484545	0.025282442	0.001968035	0.001915461
GOTERM_MF_DIRECT	GO:0003724~RNA helicase activity	7	4.022988506	7.22E-05	DHX30, DDX5, DDX3X, DHX9, DDX1, G3BP1, DDX21	173	77	18908	9.935890699	0.027888592	0.002577818	0.002381038
GOTERM_MF_DIRECT	GO:0016887~ATPase activity	13	7.471264368	9.98E-05	XRCC6, DDX5, DDX3X, HSPA5, XRCC5, DHX9, DDX1, ATP2B4, DDX21, DHX30, G3BP1, MCM5, MCM2	173	354	18908	4.013650763	0.03835001	0.003015579	0.002785382
GOTERM_BP_DIRECT	GO:0032508~DNA duplex unwinding	6	3.448275862	2.14E-04	XRCC6, DDX3X, XRCC5, DHX9, DDX1, G3BP1	171	62	19333	10.94114318	0.213747647	0.012644096	0.01230632
GOTERM_MF_DIRECT	GO:0016787~hydrolase activity	9	5.172413793	0.005222	XRCC6, AHCYL2, DDX5, AHCYL1, DDX3X, XRCC5, DHX9, DDX1, SLC3A2	173	291	18908	3.380251475	0.871588484	0.062193133	0.057445566

Annotation Cluster 7	Enrichment Score: 3.64125436584559											
Category	Term	Count	%	PValue	Genes	List Total	Pop Hits	Pop Total	Fold Enrichment	Bonferroni	Benjamini	FDR
GOTERM_CC_DIRECT	GO:0042645~mitochondrial nucleoid	8	4.597701149	1.07E-07	HADHB, HADHA, DHX30, TRMT10C, SHMT2, DBT, HSD17B10, LRPPRC	174	46	20580	20.56971514	2.96E-05	1.56E-06	1.31E-06
GOTERM_MF_DIRECT	GO:0003857~3-hydroxyacyl-CoA dehydrogenase activity	3	1.724137931	0.001677	HADHB, HADHA, HSD17B10	173	7	18908	46.84062758	0.482006169	0.029950772	0.027664454

GOTERM_BP_DIRECT	GO:0006635~fatty acid beta-oxidation	3	1.724137931	0.066655	HADHB, HADHA, HSD17B10	171	48	19333	7.066154971	1	0.718526853	0.699332013

Annotation Cluster 8 Enrichment Score: 3.0061096250694024

Category	Term	Count	%	PValue	Genes	List Total	Pop Hits	Pop Total	Fold Enrichment	Bonferroni	Benjamini	FDR
GOTERM_MF_DIRECT	GO:0003697~single-stranded DNA binding	8	4.597701149	8.76E-05	DHX9, HNRNPU, MCM5, YBX1, HNRNPA1, LRPPRC, RAD18, MCM2	173	115	18908	7.603116361	0.033757815	0.002868905	0.002649905
GOTERM_BP_DIRECT	GO:0006260~DNA replication	8	4.597701149	9.78E-05	FEN1, SET, DHX9, POLD1, CDK1, NAP1L1, MCM5, MCM2	171	121	19333	7.474940796	0.104116783	0.007096485	0.006906908
GOTERM_MF_DIRECT	GO:0043138~3'-5' DNA helicase activity	3	1.724137931	0.006008	DHX9, MCM5, MCM2	173	13	18908	25.22187639	0.905791328	0.069445522	0.064144337
GOTERM_MF_DIRECT	GO:0003688~DNA replication origin binding	3	1.724137931	0.018363	DHX9, MCM5, MCM2	173	23	18908	14.25584318	0.999300468	0.176013472	0.162577329

Annotation Cluster 9 Enrichment Score: 2.583911509820779

Category	Term	Count	%	PValue	Genes	List Total	Pop Hits	Pop Total	Fold Enrichment	Bonferroni	Benjamini	FDR
GOTERM_BP_DIRECT	GO:0048511~rhythmic process	9	5.172413793	3.81E-07	DDB1, USP7, DDX5, CSNK2A1, CDK5, PRKDC, DHX9, USP9X, CDK1	171	77	19333	13.21462748	4.28E-04	4.75E-05	4.62E-05
GOTERM_BP_DIRECT	GO:0018107~peptidyl-threonine phosphorylation	6	3.448275862	4.04E-04	CSNK2A1, CDK5, PRKDC, CSNK2A2, CSNK2B, CDK1	171	71	19333	9.554237707	0.364971991	0.02159955	0.021022536
GOTERM_MF_DIRECT	GO:0016301~kinase activity	8	4.597701149	0.004723	CSNK2A1, CDK5, PRKDC, CHEK2, CSNK2B, SKP2, IP6K1, IP6K2	173	226	18908	3.868842396	0.84364255	0.059869291	0.055299116
GOTERM_BP_DIRECT	GO:0006468~protein phosphorylation	11	6.32183908	0.013706	ILF3, CCNB1, CSNK2A1, CDK5, PRKDC, CHEK2, CSNK2A2, CSNK2B, CDK1, EIF2A, SRPK1	171	503	19333	2.472451839	0.999999817	0.334607864	0.325669096
GOTERM_BP_DIRECT	GO:0018105~peptidyl-serine phosphorylation	6	3.448275862	0.022724	CSNK2A1, CDK5, PRKDC, CHEK2, CSNK2A2, CDK1	171	182	19333	3.727202622	1	0.455706086	0.443532282
GOTERM_MF_DIRECT	GO:0004674~protein serine/threonine kinase activity	9	5.172413793	0.027273	CSNK2A1, CDK5, PRKDC, CHEK2, CSNK2A2, CSNK2B, CDK1, GTF2I, SRPK1	173	392	18908	2.509319335	0.999980389	0.214363755	0.19800011
GOTERM_MF_DIRECT	GO:0004672~protein kinase activity	7	4.022988506	0.132575	CSNK2A1, CDK5, PRKDC, CHEK2, CSNK2A2, CDK1, SRPK1	173	379	18908	2.018637424	1	0.627735378	0.579816647

Annotation Cluster 10 Enrichment Score: 2.3703108885018924

Category	Term	Count	%	PValue	Genes	List Total	Pop Hits	Pop Total	Fold Enrichment	Bonferroni	Benjamini	FDR
GOTERM_BP_DIRECT	GO:1903241~U2-type prespliceosome assembly	4	2.298850575	0.00118	SF3B3, SNRPA1, SNRPF, SNRPB	171	24	19333	18.84307992	0.734830633	0.049089734	0.047778343
GOTERM_CC_DIRECT	GO:0005686~U2 snRNP	4	2.298850575	0.001816	SF3B3, SNRPA1, SNRPF, SNRPB	174	29	20580	16.31391201	0.396752256	0.013288635	0.011185398
GOTERM_CC_DIRECT	GO:0005689~U12-type spliceosomal complex	4	2.298850575	0.001816	SF3B3, YBX1, SNRPF, SNRPB	174	29	20580	16.31391201	0.396752256	0.013288635	0.011185398
GOTERM_CC_DIRECT	GO:0030532~small nuclear ribonucleoprotein complex	3	1.724137931	0.006865	SNRPA1, SNRPF, SNRPB	174	15	20580	23.65517241	0.85267517	0.042411867	0.035699197
GOTERM_CC_DIRECT	GO:0071005~U2-type precatalytic spliceosome	4	2.298850575	0.008567	SF3B3, SNRPA1, SNRPF, SNRPB	174	50	20580	9.462068966	0.908536346	0.049615706	0.041762861
GOTERM_CC_DIRECT	GO:0071007~U2-type catalytic step 2 spliceosome	3	1.724137931	0.026197	SNRPA1, SNRPF, SNRPB	174	30	20580	11.82758621	0.999376304	0.127767631	0.107545416

Annotation Cluster 11 Enrichment Score: 2.1446016257083134

Category	Term	Count	%	PValue	Genes	List Total	Pop Hits	Pop Total	Fold Enrichment	Bonferroni	Benjamini	FDR
GOTERM_CC_DIRECT	GO:0070418~DNA-dependent protein kinase complex	3	1.724137931	4.17E-04	XRCC6, XRCC5, PRKDC	174	4	20580	88.70689655	0.109455784	0.004139227	0.003484098
GOTERM_BP_DIRECT	GO:0006302~double-strand break repair	6	3.448275862	4.59E-04	FEN1, PARP1, XRCC5, PRKDC, CHEK2, DDX1	171	73	19333	9.29247777	0.403410237	0.023452157	0.022825653

GOTERM_MF_DIRECT	GO:0003690~double-stranded DNA binding	7	4.022988506	7.06E-04	XRCC6, FEN1, XRCC5, PRKDC, DHX9, HNRNPU, YBX1	173	117	18908	6.53900499	0.241809934	0.014601547	0.013486925
GOTERM_CC_DIRECT	GO:0070419~nonhomologous end joining complex	3	1.724137931	0.001903	XRCC6, XRCC5, PRKDC	174	8	20580	44.35344828	0.411103159	0.01356411	0.011417273
GOTERM_BP_DIRECT	GO:0002218~activation of innate immune response	4	2.298850575	0.002506	XRCC6, XRCC5, PRKDC, MATR3	171	31	19333	14.58819091	0.940388078	0.087933115	0.085584056
GOTERM_BP_DIRECT	GO:0048660~regulation of smooth muscle cell proliferation	3	1.724137931	0.004013	XRCC6, XRCC5, PRKDC	171	11	19333	30.83413078	0.989108061	0.125184563	0.121840363
GOTERM_CC_DIRECT	GO:0032993~protein-DNA complex	4	2.298850575	0.006799	XRCC6, PARP1, XRCC5, PRKDC	174	46	20580	10.28485757	0.849915357	0.042411867	0.035699197
GOTERM_BP_DIRECT	GO:0000723~telomere maintenance	4	2.298850575	0.015153	XRCC6, PARP1, XRCC5, PRKDC	171	59	19333	7.664981663	0.999999965	0.340080136	0.330995181
GOTERM_BP_DIRECT	GO:0006303~double-strand break repair via nonhomologous end joining	3	1.724137931	0.037839	XRCC6, XRCC5, PRKDC	171	35	19333	9.690726817	1	0.532390034	0.518167683
GOTERM_BP_DIRECT	GO:0007420~brain development	4	2.298850575	0.433926	XRCC6, XRCC5, PRKDC, SNRPB	171	274	19333	1.650488752	1	1	0.974153298
GOTERM_CC_DIRECT	GO:0005667~transcription factor complex	3	1.724137931	0.586508	XRCC6, PARP1, PRKDC	174	234	20580	1.516357206	1	1	0.844765343

Annotation Cluster 12 — Enrichment Score: 1.782293069354151

Category	Term	Count	%	PValue	Genes	List Total	Pop Hits	Pop Total	Fold Enrichment	Bonferroni	Benjamini	FDR
GOTERM_BP_DIRECT	GO:0006511~ubiquitin-dependent protein catabolic process	10	5.747126437	8.77E-04	DDB1, USP7, USP10, USP9X, USP11, CUL1, UBR4, UBE4A, SKP2, CUL4B	171	281	19333	4.023433435	0.626810717	0.039374437	0.038322582
GOTERM_BP_DIRECT	GO:0016579~protein deubiquitination	5	2.873563218	0.015248	USP7, USP10, USP9X, USP11, CDK1	171	108	19333	5.234188867	0.999999968	0.340080136	0.330995181
GOTERM_MF_DIRECT	GO:0004197~cysteine-type endopeptidase activity	4	2.298850575	0.058698	USP7, USP10, USP9X, USP11	173	97	18908	4.507001967	1	0.360439981	0.332925479
GOTERM_MF_DIRECT	GO:0004843~thiol-dependent ubiquitin-specific protease activity	4	2.298850575	0.09467	USP7, USP10, USP9X, USP11	173	119	18908	3.673774712	1	0.458460235	0.42346327

Annotation Cluster 13 — Enrichment Score: 1.2227830965938313

Category	Term	Count	%	PValue	Genes	List Total	Pop Hits	Pop Total	Fold Enrichment	Bonferroni	Benjamini	FDR
GOTERM_BP_DIRECT	GO:0000082~G1/S transition of mitotic cell cycle	4	2.298850575	0.027478	RPS6, CUL1, SKP2, CUL4B	171	74	19333	6.111269164	1	0.482097432	0.469218605
GOTERM_BP_DIRECT	GO:0031146~SCF-dependent proteasomal ubiquitin-dependent protein catabolic process	3	1.724137931	0.071578	CUL1, SKP2, CUL4B	171	50	19333	6.783508772	1	0.737445833	0.717745588
GOTERM_CC_DIRECT	GO:0019005~SCF ubiquitin ligase complex	3	1.724137931	0.109115	CUL1, SKP2, CUL4B	174	67	20580	5.295934122	1	0.388897241	0.327345159

Annotation Cluster 14 — Enrichment Score: 0.7332962442546522

Category	Term	Count	%	PValue	Genes	List Total	Pop Hits	Pop Total	Fold Enrichment	Bonferroni	Benjamini	FDR
GOTERM_CC_DIRECT	GO:0034774~secretory granule lumen	4	2.298850575	0.072987	XRCC6, DDX3X, XRCC5, CSNK2B	174	115	20580	4.113943028	0.999999999	0.286516494	0.241168559
GOTERM_CC_DIRECT	GO:1904813~ficolin-1-rich granule lumen	4	2.298850575	0.086922	XRCC6, DDX3X, CSNK2B, ILF2	174	124	20580	3.815350389	1	0.326544838	0.274861482
GOTERM_CC_DIRECT	GO:0005576~extracellular region	10	5.747126437	0.994798	XRCC6, ILF3, DDX3X, XRCC5, CSNK2B, HNRNPA2B1, FLNA, YBX1, HNRNPC, ILF2	174	2131	20580	0.555025162	1	1	0.994798195
