# Supplementary material for: Interaction with IP6K1 supports pyrophosphorylation of substrate proteins by the inositol pyrophosphate 5-InsP7
Source: Biosci Rep. 2024 Oct 4;44(10):BSR20240792. doi: 10.1042/BSR20240792 (PMC11461180; doi:10.1042/BSR20240792)

Supplementary data for Figure 2

(merged chemiluminescence and white light epi-illumination molecular weight marker images)

2A

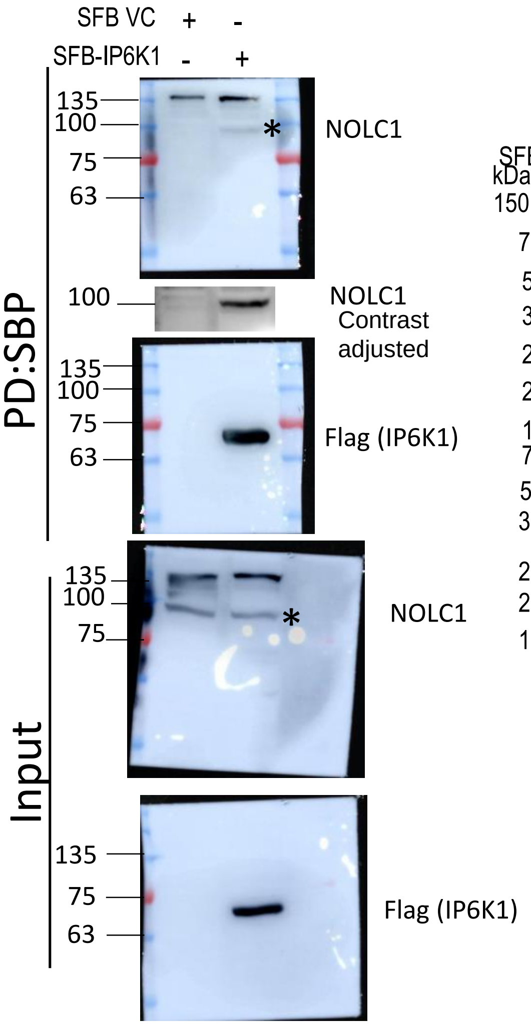

2B

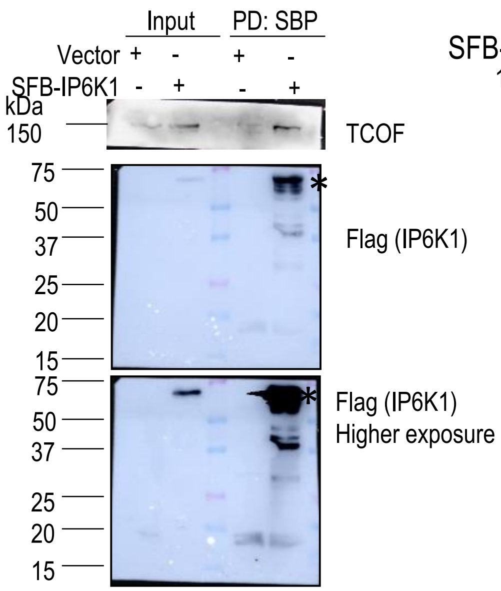

2C

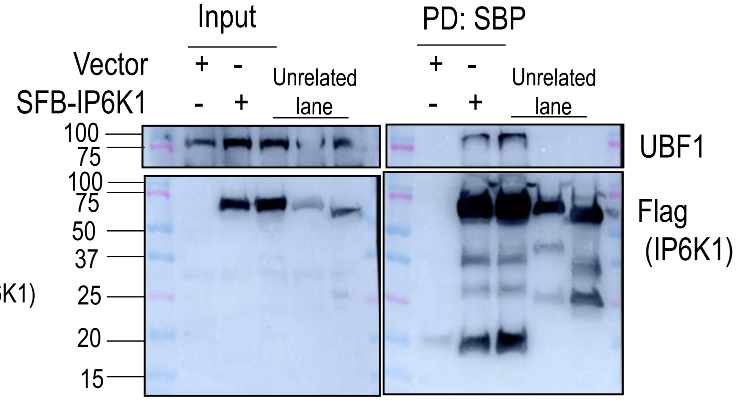

* Denotes specific band

Figure 2D

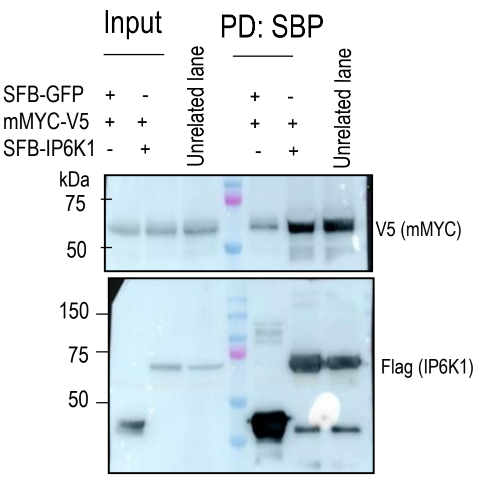

Figure 2E

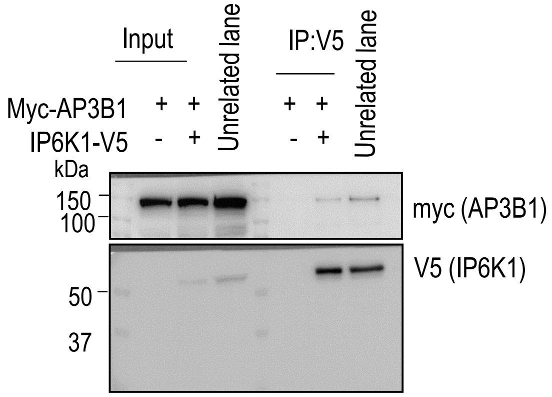

Figure 2F

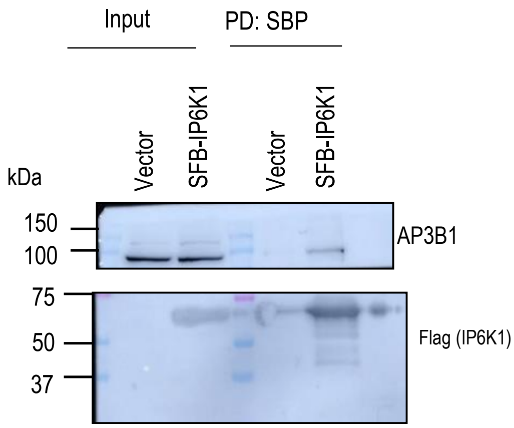

Supplementary data for Figure 2
(merged chemiluminescence and white light epi-illumination molecular weight marker images)

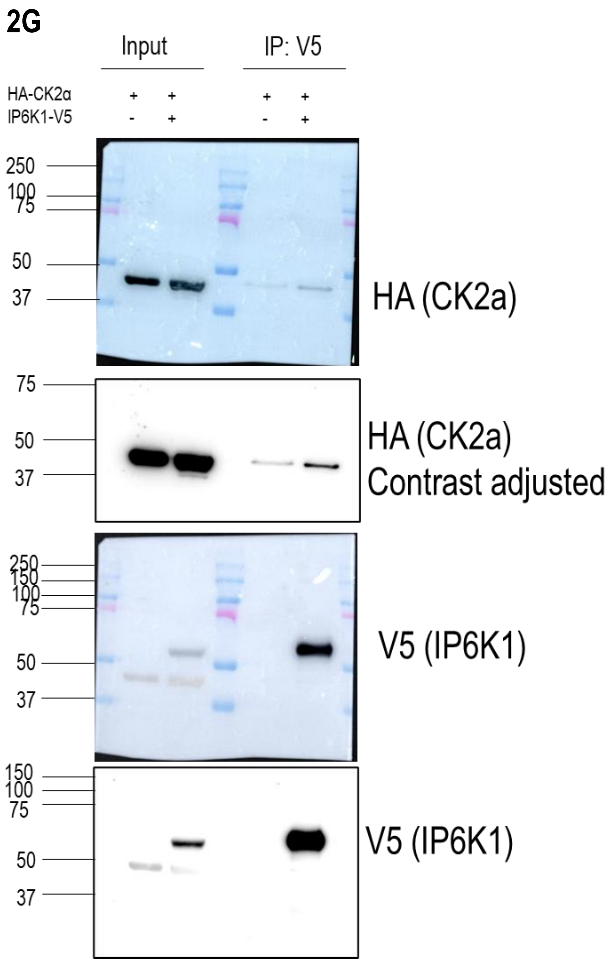

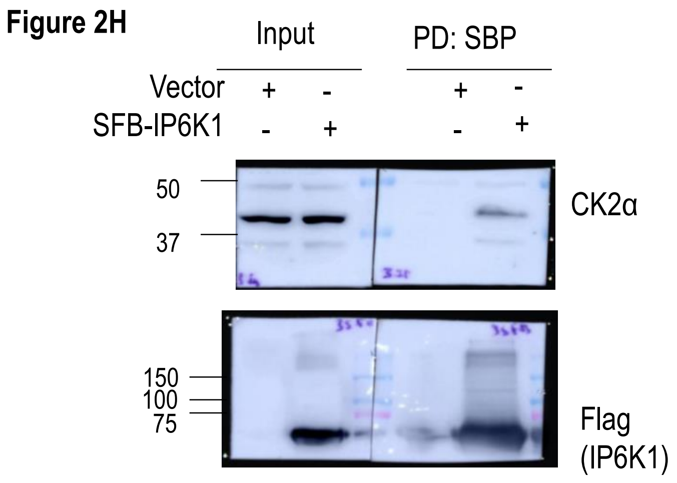

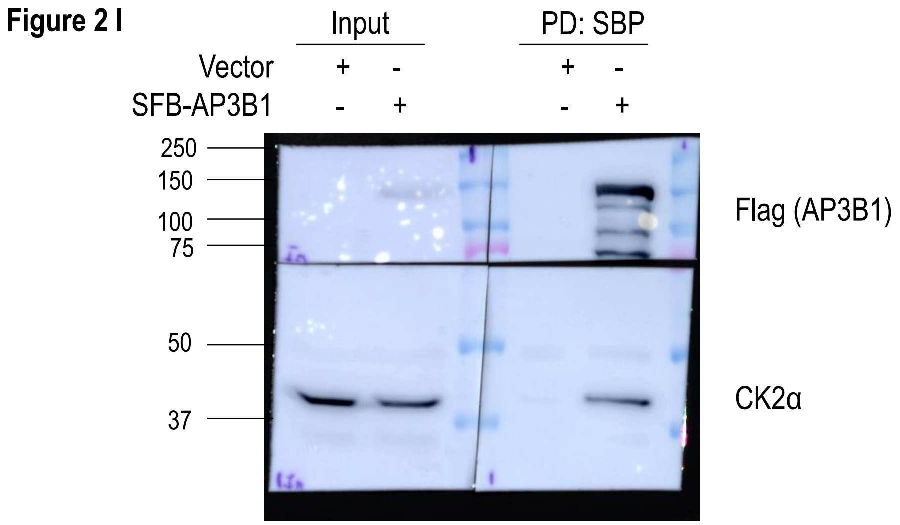

Supplementary data for Figure 3

(merged chemiluminescence and white light epi-illumination molecular weight marker images)

3A_replicate 1 Representative figure

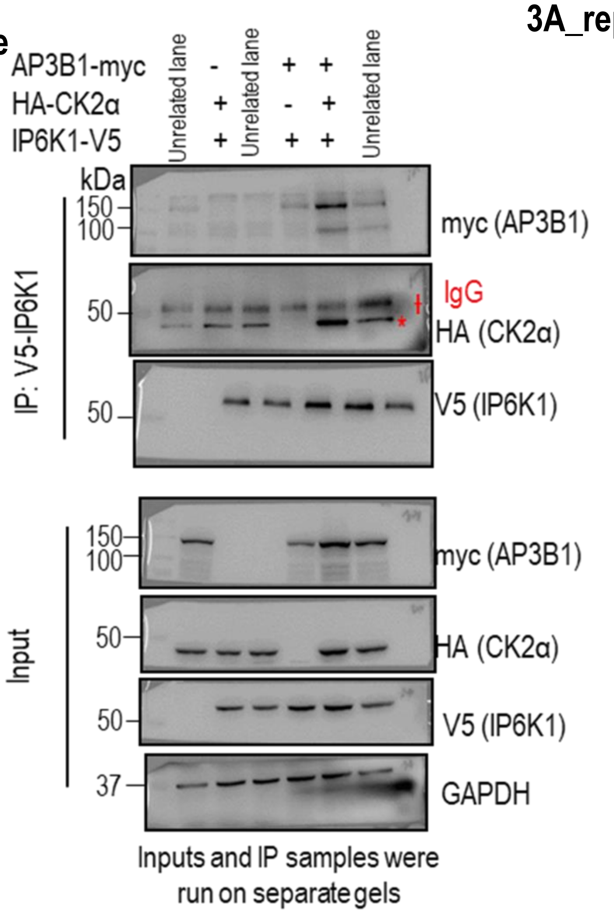

3A_replicate 2

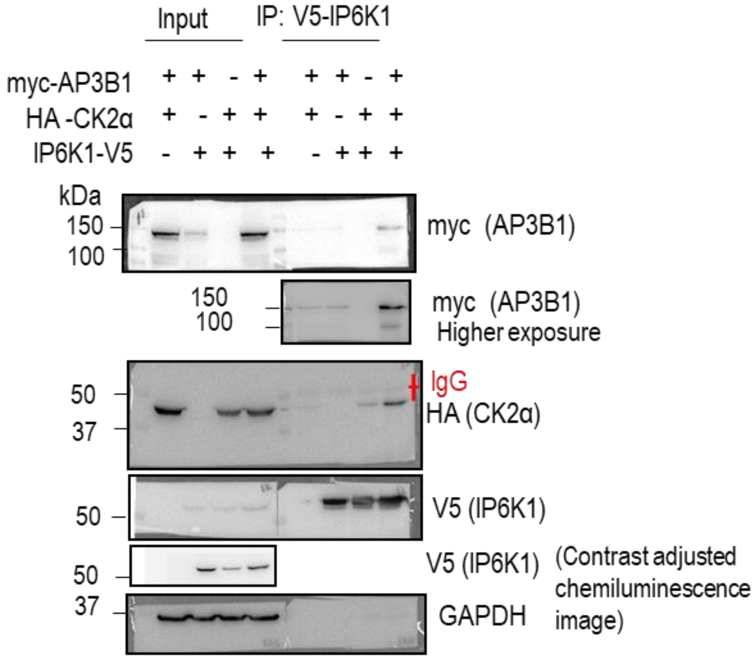

3A_1, 3A_2, 3A_3 are the biological replicates used for immunoprecipitation quantification of CK2α and AP3B1 in Fig 3 B and 3 C, respectively.

3A_ replicate 3

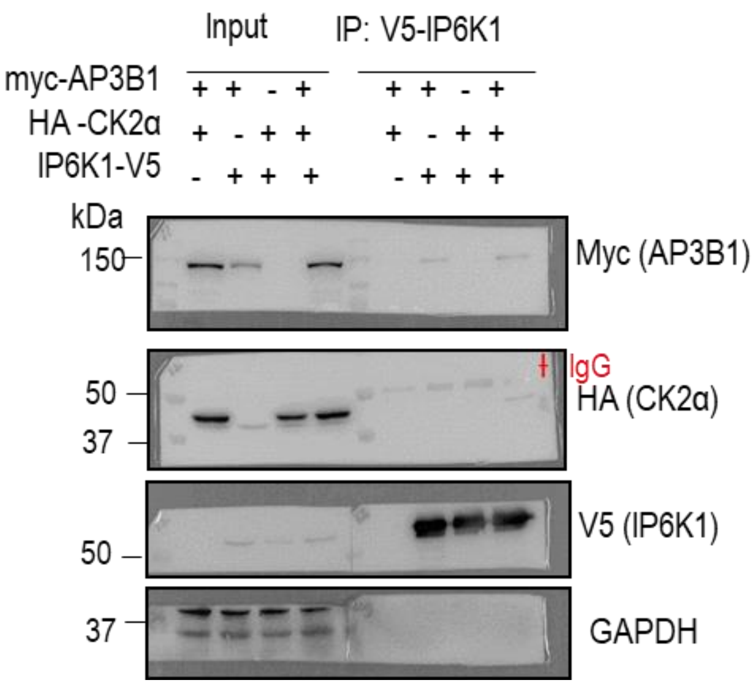

Supplementary data for Figure 3

(merged chemiluminescence and white light epi-illumination molecular weight marker images)

Figure 3D

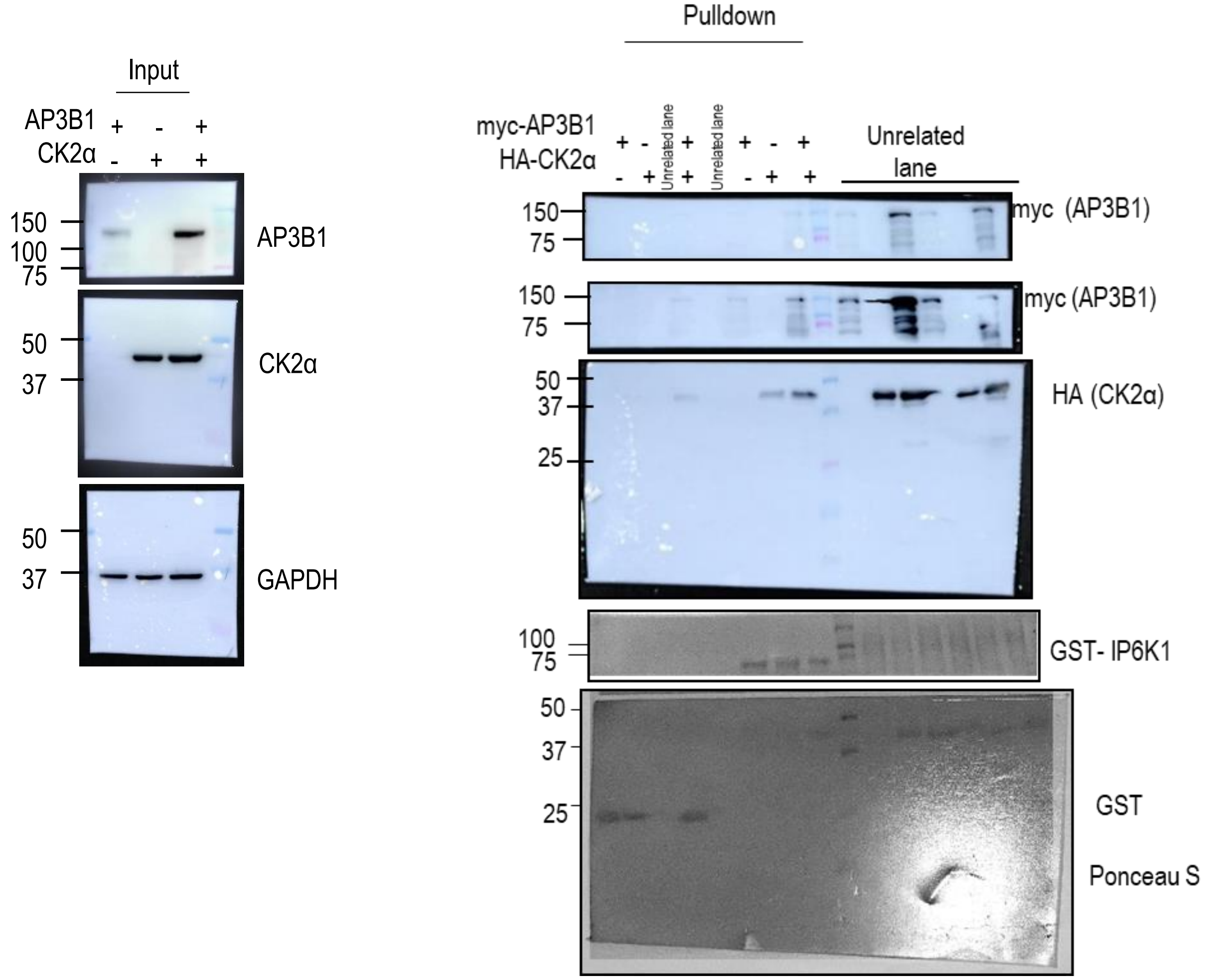

Figure 3E

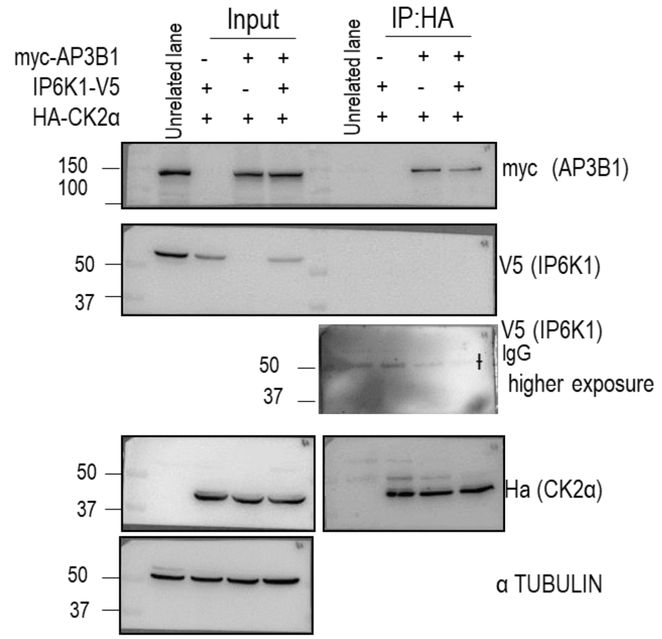

Figure 3F

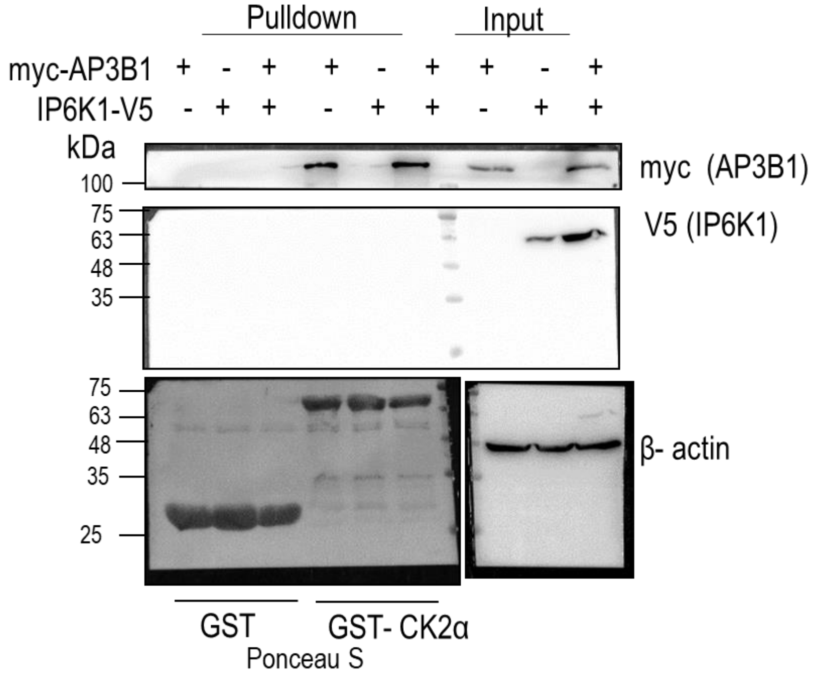

Supplementary data for Figure 3

(merged chemiluminescence and white light epi-illumination molecular weight marker images)

Figure 3G

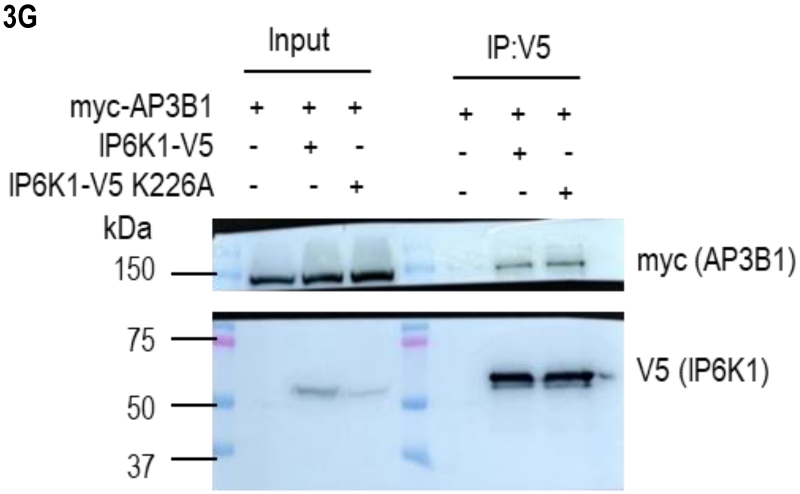

Figure 3H

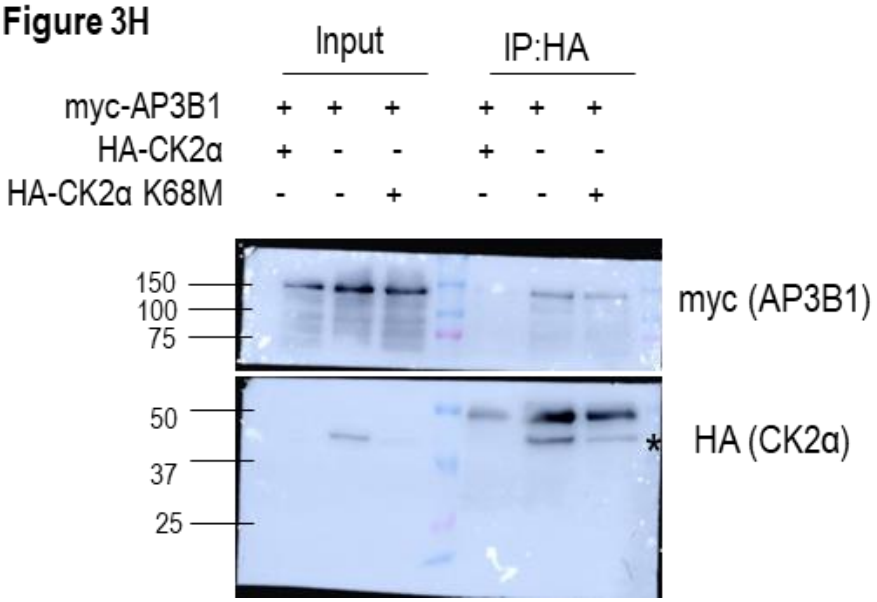

Supplementary data for Figure 4
(merged chemiluminescence and white light epi-illumination molecular weight marker images)

Figure 4A_Representative figure

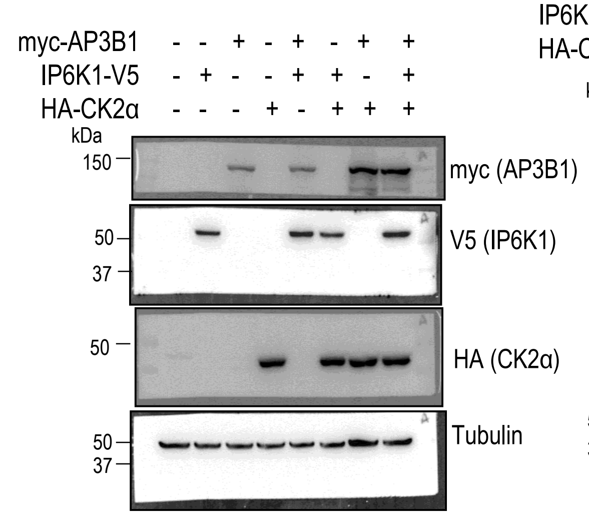

Figure 4A_replicate 2

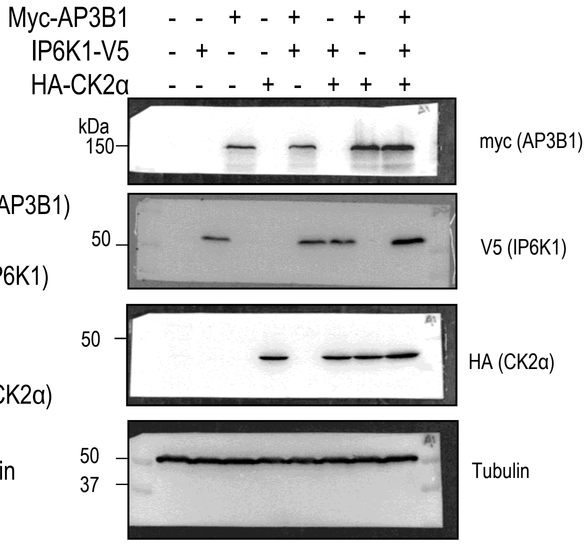

Figure 4A_replicate 3

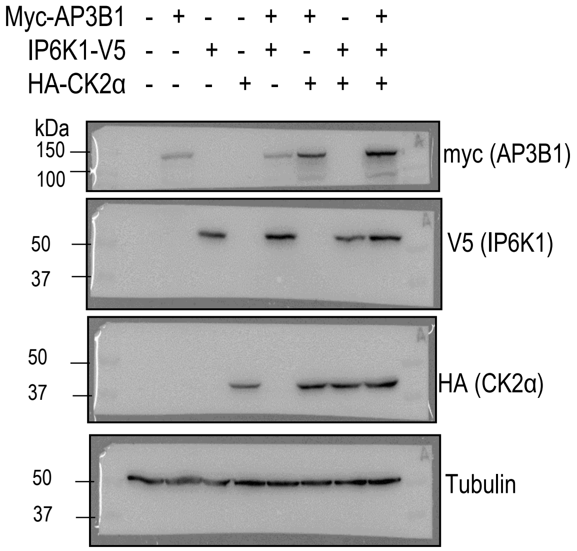

4A_1, 4A_2, 4A_3 are the biological replicates used for quantification of AP3B1 levels in presence of CK2 α shown in figure 4C

Figure 4B_Representative figure

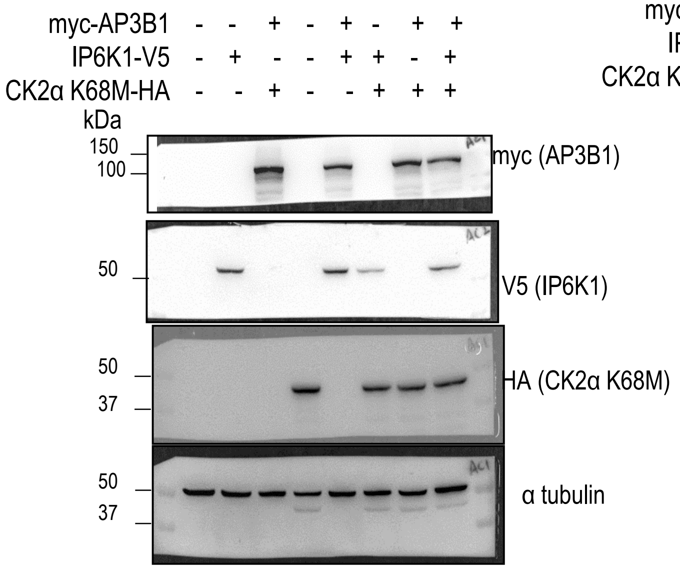

Figure 4B_replicate 2

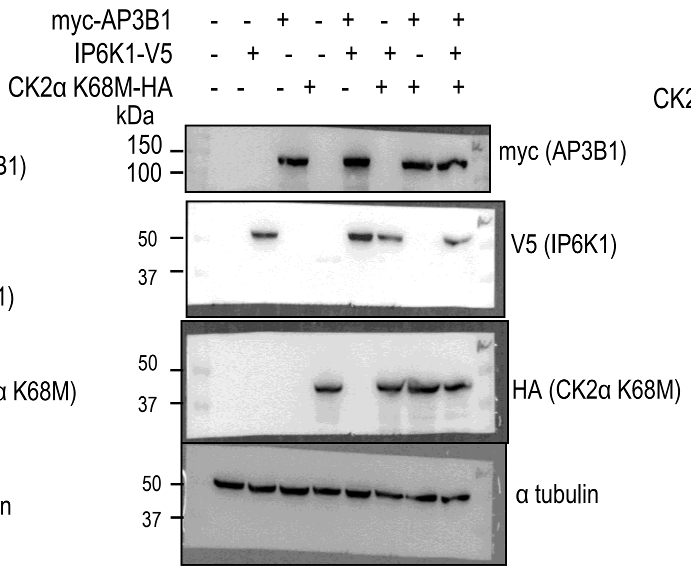

Figure 4B_replicate 3

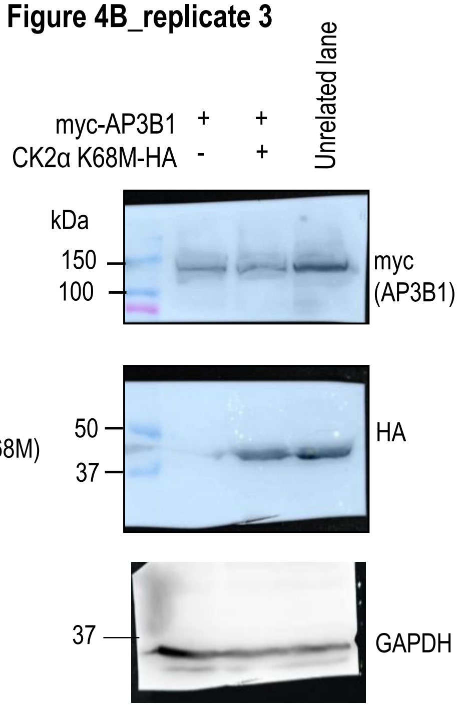

4B_1, 4B_2, 4B_3 are the biological replicates used for quantification of AP3B1 levels in presence of CK2 α shown in figure 4C

Supplementary data for Figure 4
(merged chemiluminescence and white light epi-illumination molecular weight marker images)

Figure 4D

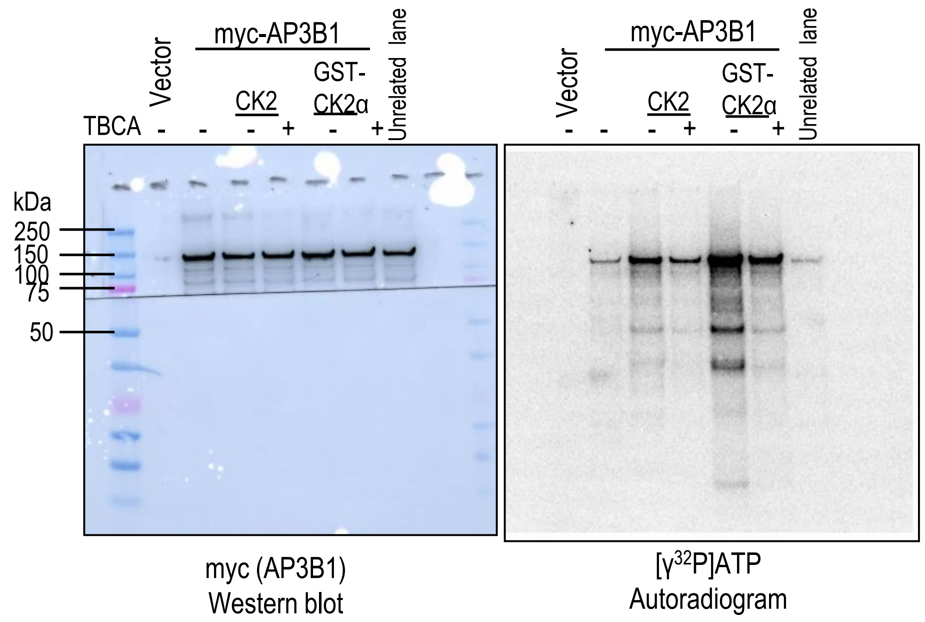

Figure 4F

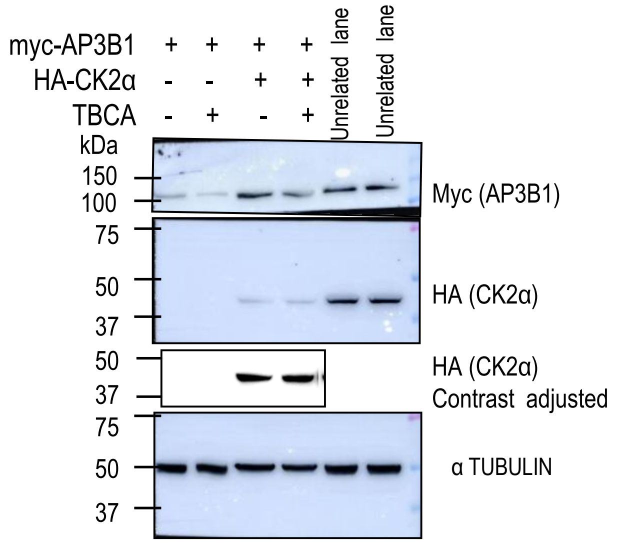

Supplementary data for Figure 5

(merged chemiluminescence and white light epi-illumination molecular weight marker images)

Figure 5B

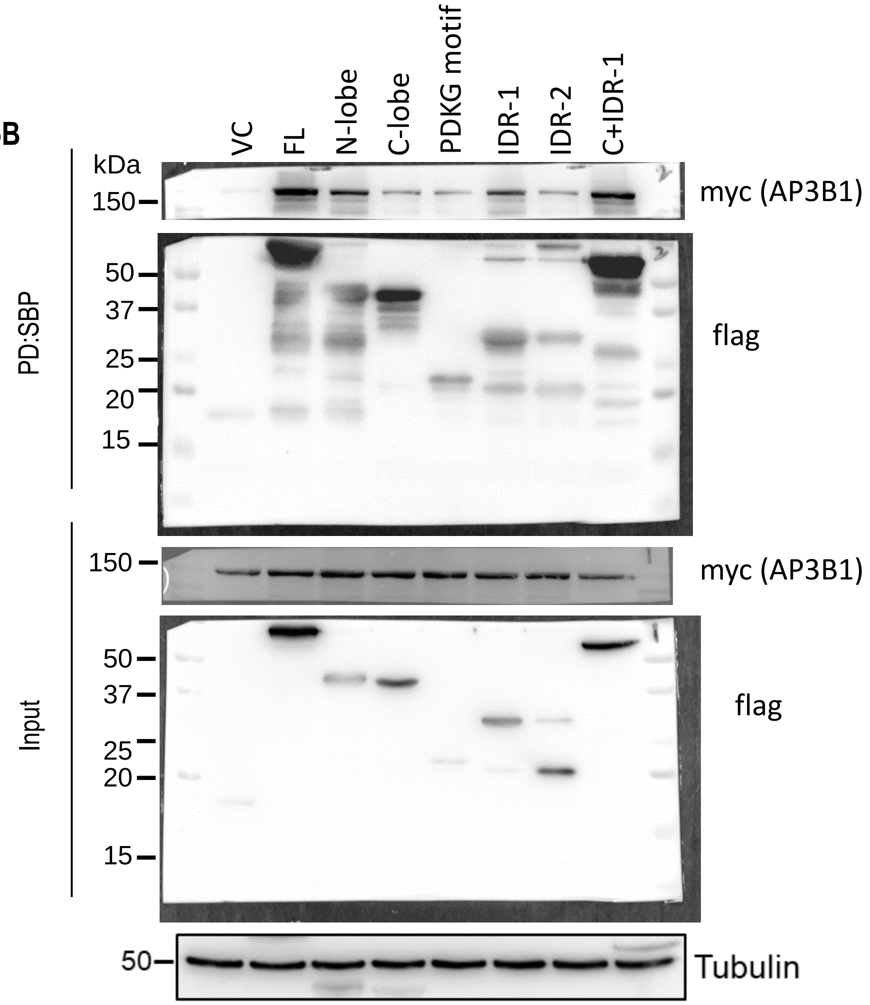

Figure 5D

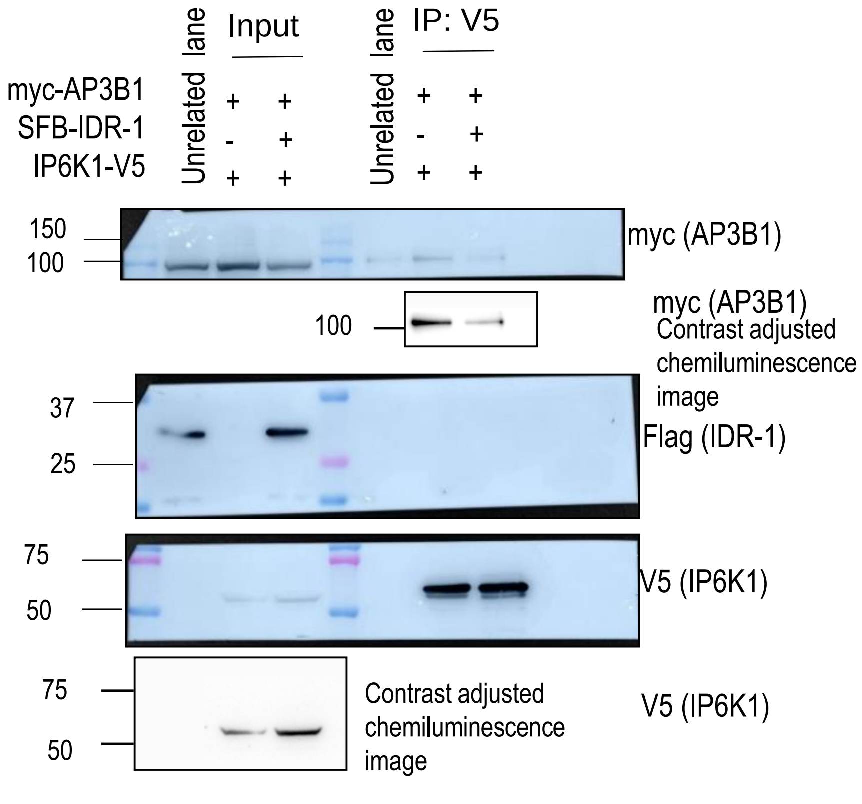

Supplementary data for Figure 6

(merged chemiluminescence and white light epi-illumination molecular weight marker images)

Figure 6A

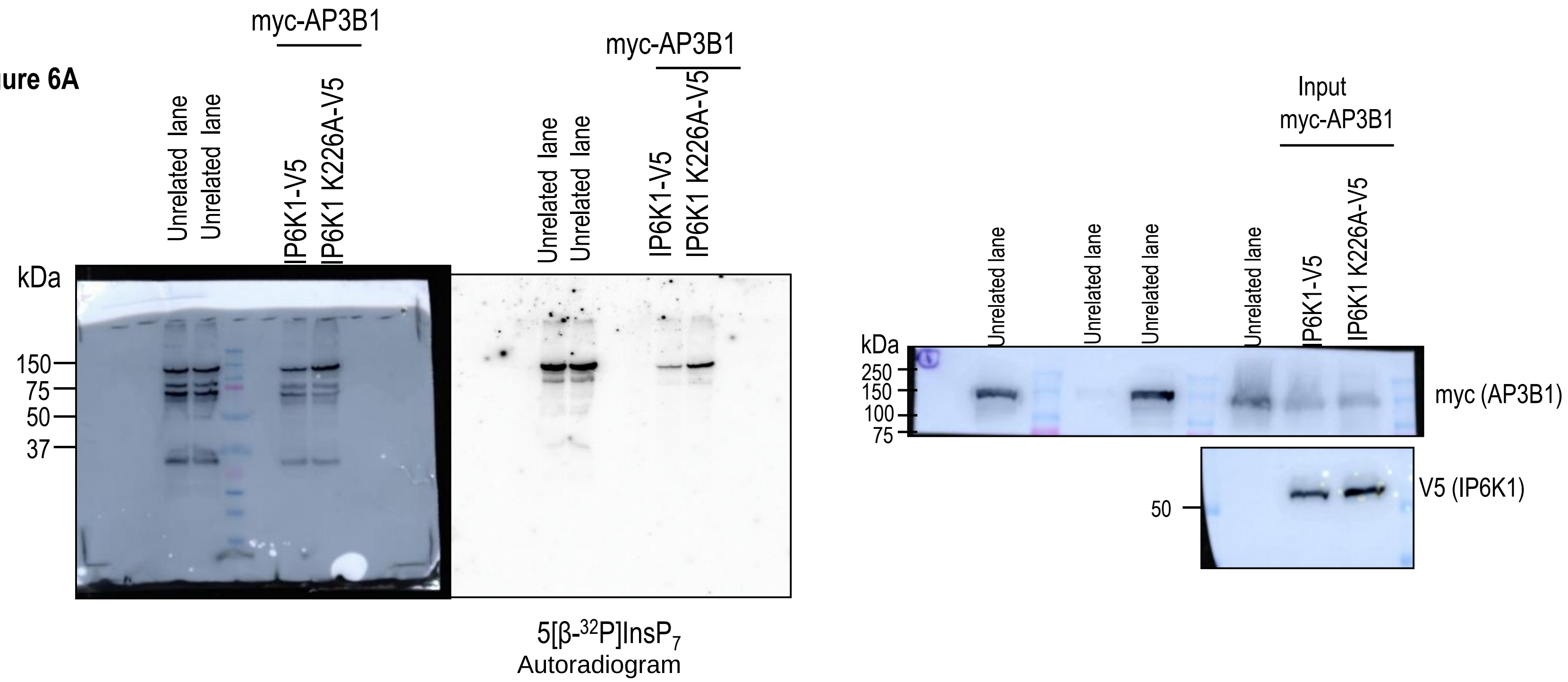

Figure 6D

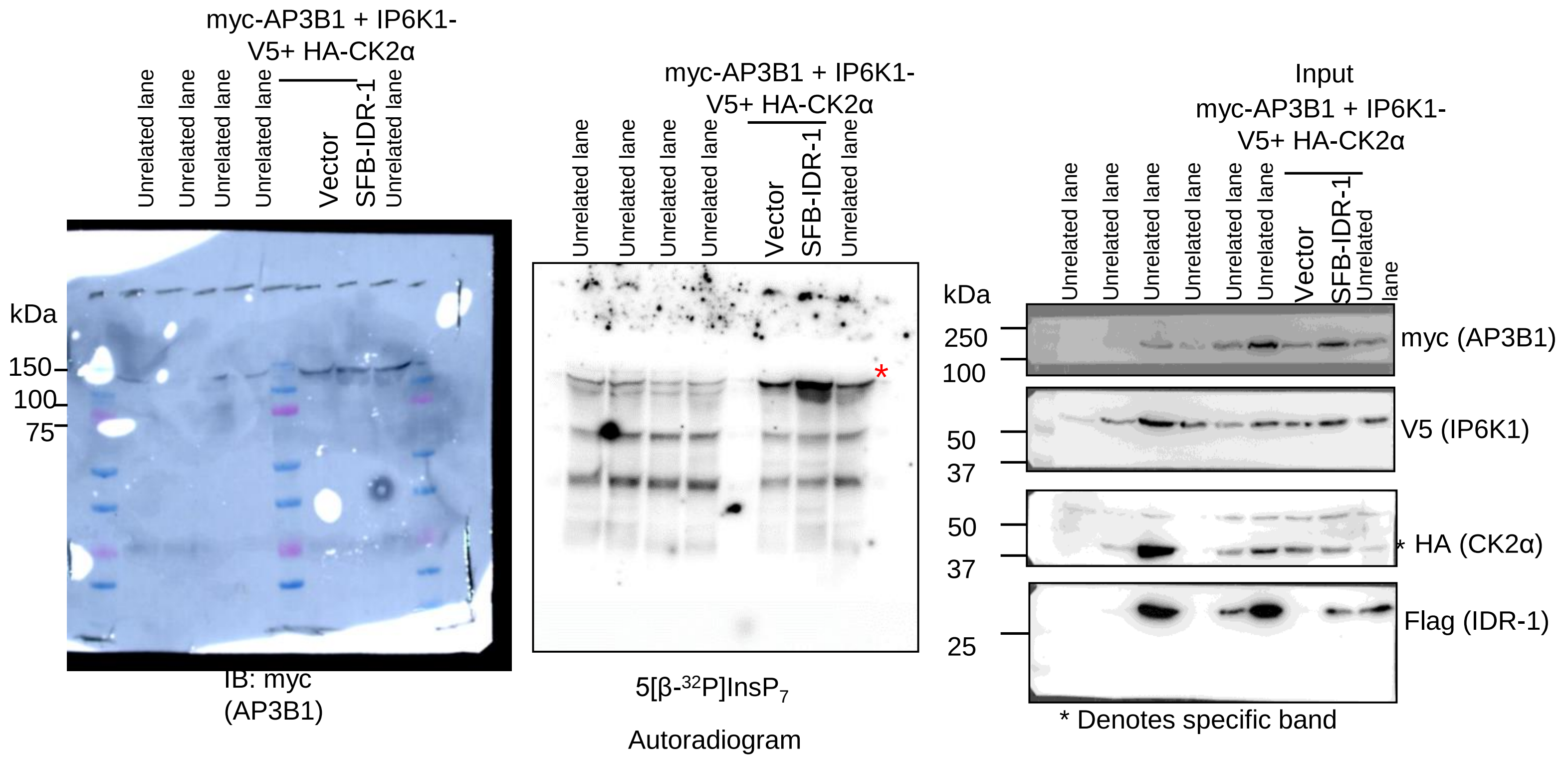

Supplement: Supplementary Tables S1-S3 [file BSR-2024-0792_supp.zip › BSR-2024-0792_suppsf.pdf]
