# Supplementary material for: Interaction with IP6K1 supports pyrophosphorylation of substrate proteins by the inositol pyrophosphate 5-InsP7
Source: Biosci Rep. 2024 Oct 4;44(10):BSR20240792. doi: 10.1042/BSR20240792 (PMC11461180; doi:10.1042/BSR20240792)
Supplement: Supplementary Tables S1-S3 [file BSR-2024-0792_supp.zip › BSR-2024-0792_suppst1.pdf]

GFP_Replicate 1 (GFP_1)

Unique peptides	Total peptides	reference	Gene Symbol	MWT(kDa)	AVG
25	27	sp|Q13085|ACACA_HUMAN	ACACA	265.38	2.876
13	340	GFP_Aequorea	GFP_Aequorea	26.87	2.8253
13	18	sp|P11142|HSP7C_HUMAN	HSPA8	70.85	3.4056
12	20	sp|Q9BQE3|TBA1C_HUMAN	TUBA1C	49.86	3.0531
12	15	sp|P68371|TBB4B_HUMAN	TUBB4B	49.8	2.9777
9	11	sp|P0DMV9|HS71B_HUMAN	HSPA1B	70.01	3.0861
9	10	sp|P34931|HS71L_HUMAN	HSPA1L	70.33	2.7971
8	8	sp|P38646|GRP75_HUMAN	HSPA9	73.63	3.2366
7	7	sp|Q96RQ3|MCCA_HUMAN	MCCC1	80.42	2.6025
6	9	sp|P12236|ADT3_HUMAN	SLC25A6	32.85	2.4643
5	7	sp|P05166|PCCB_HUMAN	PCCB	58.18	3.6281
5	7	sp|P62805|H4_HUMAN	HIST1H4A	11.36	2.7755
5	6	sp|P62736|ACTA_HUMAN	ACTA2	41.98	2.7629
4	5	sp|P54652|HSP72_HUMAN	HSPA2	69.98	3.011
4	4	sp|P07437|TBB5_HUMAN	TUBB	49.64	3.3287
4	4	sp|P11498|PYC_HUMAN	PC	129.55	3.145
4	4	sp|Q96KK5|H2A1H_HUMAN	HIST1H2AH	13.9	2.8798
4	4	sp|P32119|PRDX2_HUMAN	PRDX2	21.88	2.7509
3	3	sp|P62987|RL40_HUMAN	UBA52	14.72	3.2589
3	3	sp|P07355|ANXA2_HUMAN	ANXA2	38.58	3.2122
3	3	sp|P51571|SSRD_HUMAN	SSR4	18.99	3.1548
3	3	sp|P33778|H2B1B_HUMAN	HIST1H2BB	13.94	2.9824
3	3	sp|P25311|ZA2G_HUMAN	AZGP1	34.24	2.9204
3	3	sp|P04040|CATA_HUMAN	CAT	59.72	2.7696
3	3	sp|Q92551|IP6K1_HUMAN	IP6K1	50.2	2.4614
2	4	sp|Q92820|GGH_HUMAN	GGH	35.94	2.7933
2	3	sp|Q13257|MD2L1_HUMAN	MAD2L1	23.5	4.0886
2	3	sp|P11021|GRP78_HUMAN	HSPA5	72.29	3.525
2	3	sp|P10599|THIO_HUMAN	TXN	11.73	2.639
2	2	sp|P05141|ADT2_HUMAN	SLC25A5	32.83	3.6893
2	2	sp|P12273|PIP_HUMAN	PIP	16.56	3.6083
2	2	sp|P68104|EF1A1_HUMAN	EEF1A1	50.11	3.3776
2	2	sp|Q13867|BLMH_HUMAN	BLMH	52.53	3.3704
2	2	sp|Q15517|CDSN_HUMAN	CDSN	51.49	3.1774
2	2	sp|Q9HCC0|MCCB_HUMAN	MCCC2	61.29	3.1232
2	2	sp|P07900|HS90A_HUMAN	HSP90AA1	84.61	3.1018
2	2	sp|P04792|HSPB1_HUMAN	HSPB1	22.77	2.9531
2	2	sp|Q01469|FABP5_HUMAN	FABP5	15.15	2.9226
2	2	sp|P52272|HNRPM_HUMAN	HNRNPM	77.46	2.9086
2	2	sp|P56537|IF6_HUMAN	EIF6	26.58	2.8361
2	2	sp|P05165|PCCA_HUMAN	PCCA	80.01	2.6936
2	2	sp|Q58FF7|H90B3_HUMAN	HSP90AB3P	68.28	2.3001
2	2	sp|Q71DI3|H32_HUMAN	HIST2H3A	15.38	2.2317
2	2	sp|Q06830|PRDX1_HUMAN	PRDX1	22.1	2.1947
1	2	sp|P10809|CH60_HUMAN	HSPD1	61.02	3.9041
1	2	sp|P08238|HS90B_HUMAN	HSP90AB1	83.21	3.1673

1	2	tr|B4DR29|B4DR29_HUMAN	tr|B4DR29|B4DR	112.63	2.9903
1	2	sp|P68366|TBA4A_HUMAN	TUBA4A	49.89	2.8521
1	1	sp|P14618|KPYM_HUMAN	PKM	57.9	4.2053
1	1	sp|Q6UWP8|SBSN_HUMAN	SBSN	60.5	3.9784
1	1	sp|Q9UI42|CBPA4_HUMAN	CPA4	47.32	3.7502
1	1	sp|P06748|NPM_HUMAN	NPM1	32.55	3.7321
1	1	sp|Q9BVA1|TBB2B_HUMAN	TUBB2B	49.92	3.6259
1	1	sp|P06396|GELS_HUMAN	GSN	85.64	3.5807
1	1	sp|P04844|RPN2_HUMAN	RPN2	69.24	3.5634
1	1	sp|Q99707|METH_HUMAN	MTR	140.44	3.5539
1	1	sp|P04350|TBB4A_HUMAN	TUBB4A	49.55	3.4505
1	1	sp|P61626|LYSC_HUMAN	LYZ	16.53	3.2497
1	1	sp|Q8WVV4|POF1B_HUMAN	POF1B	68.02	3.1221
1	1	sp|P04406|G3P_HUMAN	GAPDH	36.03	3.0793
1	1	sp|Q16610|ECM1_HUMAN	ECM1	60.64	2.9864
1	1	sp|Q07021|C1QBP_HUMAN	C1QBP	31.34	2.8541
1	1	sp|O75223|GGCT_HUMAN	GGCT	20.99	2.8193
1	1	sp|P01834|IGKC_HUMAN	IGKC	11.76	2.7901
1	1	sp|P28072|PSB6_HUMAN	PSMB6	25.34	2.7688
1	1	tr|A0PJA5|A0PJA5_HUMAN	GCC2	48.72	2.6896
1	1	sp|P13639|EF2_HUMAN	EEF2	95.28	2.6688
1	1	sp|Q9UNE7|CHIP_HUMAN	STUB1	34.83	2.6686
1	1	sp|Q00325|MPCP_HUMAN	SLC25A3	40.07	2.6081
1	1	sp|Q9H0W9|CK054_HUMAN	C11orf54	35.09	2.6032
1	1	sp|Q13162|PRDX4_HUMAN	PRDX4	30.52	2.5278
1	1	sp|B2RXH8|HNRC2_HUMAN	HNRNPCL2	32.05	2.5098
1	1	sp|P42677|RS27_HUMAN	RPS27	9.45	2.4729
1	1	sp|P27708|PYR1_HUMAN	CAD	242.83	2.4668
1	1	sp|P62258|1433E_HUMAN	YWHAE	29.16	2.4544
1	1	sp|O14735|CDIPT_HUMAN	CDIPT	23.52	2.4076
1	1	sp|P04083|ANXA1_HUMAN	ANXA1	38.69	2.3599
1	1	sp|Q5VTQ0|TT39B_HUMAN	TTC39B	76.91	2.3011
1	1	sp|Q16650|TBR1_HUMAN	TBR1	74.01	2.236
1	1	sp|Q92665|RT31_HUMAN	MRPS31	45.29	2.2108

GFP_Replicate 2 (GFP_2)

Unique peptides	Total peptides	reference	Gene Symb	MWT(kDa)	AVG
21	370	GFP_Aequorea		26.87	2.8706
20	49	sp|P68371|TBB4B_HUMAN	TUBB4B	49.8	2.8833
19	30	sp|P38646|GRP75_HUMAN	HSPA9	73.63	2.8643
15	17	sp|P14618|KPYM_HUMAN	PKM	57.9	2.83
14	25	sp|Q9BQE3|TBA1C_HUMAN	TUBA1C	49.86	3.0295
14	16	sp|P11142|HSP7C_HUMAN	HSPA8	70.85	3.319
14	15	sp|Q13085|ACACA_HUMAN	ACACA	265.38	2.9373
13	16	sp|P10809|CH60_HUMAN	HSPD1	61.02	3.3448
13	16	sp|Q9Y230|RUVB2_HUMAN	RUVBL2	51.12	3.0322
13	14	sp|P06576|ATPB_HUMAN	ATP5B	56.52	2.9576
12	15	sp|P07814|SYEP_HUMAN	EPRS	170.48	3.1445
12	15	sp|P62258|1433E_HUMAN	YWHAE	29.16	2.8255
11	14	sp|P25705|ATPA_HUMAN	ATP5A1	59.71	3.2421
11	12	sp|P07900|HS90A_HUMAN	HSP90AA1	84.61	2.8713
11	12	sp|Q13263|TIF1B_HUMAN	TRIM28	88.49	2.8095
10	15	sp|P08238|HS90B_HUMAN	HSP90AB1	83.21	3.1136
10	12	sp|P23396|RS3_HUMAN	RPS3	26.67	2.7269
10	11	sp|P19338|NUCL_HUMAN	NCL	76.57	2.8287
10	10	sp|P49327|FAS_HUMAN	FASN	273.25	3.267
10	10	sp|P14625|ENPL_HUMAN	HSP90B1	92.41	2.9234
9	15	sp|P13489|RINI_HUMAN	RNH1	49.94	3.1961
9	11	sp|P34931|HS71L_HUMAN	HSPA1L	70.33	2.7793
9	10	sp|P41252|SYIC_HUMAN	IARS	144.41	3.0882
8	13	sp|P62736|ACTA_HUMAN	ACTA2	41.98	2.459
8	11	sp|P49368|TCPG_HUMAN	CCT3	60.5	2.7222
8	8	sp|Q9Y265|RUVB1_HUMAN	RUVBL1	50.2	3.3539
8	8	sp|Q58FF8|H90B2_HUMAN	HSP90AB2I	44.32	2.7228
7	16	sp|Q58FF7|H90B3_HUMAN	HSP90AB3I	68.28	2.7554
7	8	sp|P0DMV9|HS71B_HUMAN	HSPA1B	70.01	3.139
7	8	sp|P68104|EF1A1_HUMAN	EEF1A1	50.11	2.3106
7	7	sp|Q9P2J5|SYLC_HUMAN	LARS	134.38	3.0721
7	7	sp|P22061|PIMT_HUMAN	PCMT1	24.62	3.0409
7	7	sp|P12236|ADT3_HUMAN	SLC25A6	32.85	2.9721
7	7	sp|P07195|LDHB_HUMAN	LDHB	36.62	2.7912
7	7	sp|P52272|HNRPM_HUMAN	HNRNPM	77.46	2.6395
6	11	sp|P07437|TBB5_HUMAN	TUBB	49.64	3.33
6	9	sp|P12277|KCRB_HUMAN	CKB	42.62	3.9118
6	7	sp|P17987|TCPA_HUMAN	TCP1	60.31	2.6938
6	7	sp|P49915|GUAA_HUMAN	GMPS	76.67	2.5737
6	6	sp|Q00839|HNRPU_HUMAN	HNRNPU	90.53	3.5506
6	6	sp|P42704|LPPRC_HUMAN	LRPPRC	157.81	3.541
6	6	sp|P78371|TCPB_HUMAN	CCT2	57.45	3.4086
6	6	sp|O43175|SERA_HUMAN	PHGDH	56.61	3.0099
6	6	sp|P22314|UBA1_HUMAN	UBA1	117.77	2.9657
6	6	sp|P05023|AT1A1_HUMAN	ATP1A1	112.82	2.4796

6	6	sp|P35232|PHB_HUMAN	PHB	29.79	2.4449
5	7	sp|O14980|XPO1_HUMAN	XPO1	123.31	3.2887
5	6	sp|P63261|ACTG_HUMAN	ACTG1	41.77	3.3452
5	6	sp|Q14974|IMB1_HUMAN	KPNB1	97.11	3.1109
5	6	sp|P11586|C1TC_HUMAN	MTHFD1	101.5	2.6784
5	5	sp|P06733|ENOA_HUMAN	ENO1	47.14	3.43
5	5	sp|Q00610|CLH1_HUMAN	CLTC	191.49	3.1954
5	5	sp|Q99832|TCPH_HUMAN	CCT7	59.33	2.8193
5	5	sp|Q15758|AAAT_HUMAN	SLC1A5	56.56	2.7162
5	5	sp|Q06830|PRDX1_HUMAN	PRDX1	22.1	2.2698
4	7	sp|Q9BVA1|TBB2B_HUMAN	TUBB2B	49.92	2.6301
4	5	sp|P50991|TCPD_HUMAN	CCT4	57.89	3.1881
4	5	sp|P52292|IMA1_HUMAN	KPNA2	57.83	2.8369
4	5	sp|P54652|HSP72_HUMAN	HSPA2	69.98	2.7703
4	5	sp|Q16531|DDB1_HUMAN	DDB1	126.89	2.7503
4	4	sp|P11177|ODPB_HUMAN	PDHB	39.21	3.6354
4	4	sp|P50990|TCPQ_HUMAN	CCT8	59.58	3.466
4	4	sp|P40227|TCPZ_HUMAN	CCT6A	57.99	3.4284
4	4	sp|P61978|HNRPK_HUMAN	HNRNPK	50.94	3.4255
4	4	sp|P49588|SYAC_HUMAN	AARS	106.74	3.3256
4	4	sp|Q12931|TRAP1_HUMAN	TRAP1	80.06	3.1736
4	4	sp|P49411|EFTU_HUMAN	TUFM	49.51	3.0904
4	4	sp|P54136|SYRC_HUMAN	RARS	75.33	3.0194
4	4	sp|P06748|NPM_HUMAN	NPM1	32.55	3.0076
4	4	sp|P56192|SYMC_HUMAN	MARS	101.05	2.9763
4	4	sp|P32119|PRDX2_HUMAN	PRDX2	21.88	2.9214
4	4	sp|P29401|TKT_HUMAN	TKT	67.83	2.8636
4	4	sp|P55060|XPO2_HUMAN	CSE1L	110.35	2.8402
4	4	sp|P22695|QCR2_HUMAN	UQCRC2	48.41	2.7069
4	4	sp|P15880|RS2_HUMAN	RPS2	31.3	2.6774
4	4	sp|P25205|MCM3_HUMAN	MCM3	90.92	2.6522
4	4	sp|Q86VP6|CAND1_HUMAN	CAND1	136.29	2.6446
4	4	sp|P13639|EF2_HUMAN	EEF2	95.28	2.5405
4	4	sp|P63244|RACK1_HUMAN	RACK1	35.05	2.4495
3	5	sp|P62263|RS14_HUMAN	RPS14	16.26	2.7772
3	4	sp|P04350|TBB4A_HUMAN	TUBB4A	49.55	4.427
3	4	sp|Q5JTZ9|SYAM_HUMAN	AARS2	107.27	3.5669
3	4	sp|P34932|HSP74_HUMAN	HSPA4	94.27	3.5496
3	4	sp|P08243|ASNS_HUMAN	ASNS	64.33	3.1746
3	4	sp|P53396|ACLY_HUMAN	ACLY	120.76	2.8984
3	4	sp|P26641|EF1G_HUMAN	EEF1G	50.09	2.623
3	3	sp|P11021|GRP78_HUMAN	HSPA5	72.29	3.9888
3	3	sp|Q14240|IF4A2_HUMAN	EIF4A2	46.37	3.711
3	3	sp|P67809|YBOX1_HUMAN	YBX1	35.9	3.6431
3	3	sp|Q9H3U1|UN45A_HUMAN	UNC45A	103.01	3.3938
3	3	sp|P61981|1433G_HUMAN	YWHAG	28.28	3.3507
3	3	sp|P05388|RLA0_HUMAN	RPLP0	34.25	3.3461

3	3	sp|Q32P51|RA1L2_HUMAN	HNRNPA1L	34.2	3.2574
3	3	sp|Q96FW1|OTUB1_HUMAN	OTUB1	31.26	3.1456
3	3	sp|P28331|NDUS1_HUMAN	NDUFS1	79.42	3.1421
3	3	sp|P17980|PRS6A_HUMAN	PSMC3	49.17	3.1289
3	3	sp|P62195|PRS8_HUMAN	PSMC5	45.6	3.0804
3	3	sp|Q3ZCQ8|TIM50_HUMAN	TIMM50	39.62	3.0604
3	3	sp|O95831|AIFM1_HUMAN	AIFM1	66.86	3.0028
3	3	sp|P62987|RL40_HUMAN	UBA52	14.72	2.9392
3	3	sp|P00338|LDHA_HUMAN	LDHA	36.67	2.8833
3	3	sp|O95373|IPO7_HUMAN	IPO7	119.44	2.8535
3	3	sp|P26599|PTBP1_HUMAN	PTBP1	57.19	2.8469
3	3	sp|P04844|RPN2_HUMAN	RPN2	69.24	2.7876
3	3	sp|P27824|CALX_HUMAN	CANX	67.53	2.7129
3	3	sp|P16615|AT2A2_HUMAN	ATP2A2	114.68	2.6866
3	3	sp|P22626|ROA2_HUMAN	HNRNPA2B	37.41	2.6536
3	3	sp|Q08211|DHX9_HUMAN	DHX9	140.87	2.5411
3	3	sp|P43490|NAMPT_HUMAN	NAMPT	55.49	2.5313
3	3	sp|Q13200|PSMD2_HUMAN	PSMD2	100.14	2.2263
2	4	sp|P52597|HNRPF_HUMAN	HNRNPF	45.64	4.3521
2	3	sp|O43852|CALU_HUMAN	CALU	37.08	3.713
2	3	sp|Q13257|MD2L1_HUMAN	MAD2L1	23.5	3.7084
2	3	sp|Q01844|EWS_HUMAN	EWSR1	68.44	3.7018
2	3	sp|P62424|RL7A_HUMAN	RPL7A	29.98	3.0702
2	3	sp|Q92616|GCN1_HUMAN	GCN1	292.57	3
2	3	sp|P35998|PRS7_HUMAN	PSMC2	48.6	2.8236
2	3	sp|Q53H12|AGK_HUMAN	AGK	47.11	2.6253
2	2	sp|P46940|IQGA1_HUMAN	IQGAP1	189.13	4.3983
2	2	sp|Q8NEZ5|FBX22_HUMAN	FBXO22	44.48	4.0331
2	2	sp|Q96P70|IPO9_HUMAN	IPO9	115.89	3.9672
2	2	sp|P05455|LA_HUMAN	SSB	46.81	3.9367
2	2	sp|P0DN79|CBSL_HUMAN	CBSL	60.55	3.9128
2	2	sp|P16989|YBOX3_HUMAN	YBX3	40.07	3.8342
2	2	sp|O14983|AT2A1_HUMAN	ATP2A1	110.18	3.635
2	2	sp|P55072|TERA_HUMAN	VCP	89.27	3.5274
2	2	sp|P05141|ADT2_HUMAN	SLC25A5	32.83	3.4747
2	2	sp|Q9Y3F4|STRAP_HUMAN	STRAP	38.41	3.4436
2	2	sp|P63104|1433Z_HUMAN	YWHAZ	27.73	3.4196
2	2	sp|O94952|FBX21_HUMAN	FBXO21	72.22	3.3517
2	2	sp|P09874|PARP1_HUMAN	PARP1	113.01	3.3464
2	2	sp|P22234|PUR6_HUMAN	PAICS	47.05	3.3224
2	2	sp|Q14566|MCM6_HUMAN	MCM6	92.83	3.2462
2	2	sp|P62701|RS4X_HUMAN	RPS4X	29.58	3.2399
2	2	sp|Q9BV20|MTNA_HUMAN	MRI1	39.13	3.1848
2	2	sp|Q14103|HNRPD_HUMAN	HNRNPD	38.41	3.1516
2	2	sp|Q9GZZ9|UBA5_HUMAN	UBA5	44.83	3.1444
2	2	sp|P04406|G3P_HUMAN	GAPDH	36.03	3.1238
2	2	sp|P48643|TCPE_HUMAN	CCT5	59.63	3.1228

2	2	sp|P04843|RPN1_HUMAN	RPN1	68.53	3.1155
2	2	sp|P12956|XRCC6_HUMAN	XRCC6	69.8	3.1151
2	2	sp|Q9NZI8|IF2B1_HUMAN	IGF2BP1	63.44	3.0942
2	2	sp|P62913|RL11_HUMAN	RPL11	20.24	3.0518
2	2	sp|P33993|MCM7_HUMAN	MCM7	81.26	3.045
2	2	sp|P27708|PYR1_HUMAN	CAD	242.83	3.0223
2	2	sp|P28066|PSA5_HUMAN	PSMA5	26.39	3.0137
2	2	sp|O14818|PSA7_HUMAN	PSMA7	27.87	3.0133
2	2	sp|Q92841|DDX17_HUMAN	DDX17	80.22	2.9866
2	2	sp|P04792|HSPB1_HUMAN	HSPB1	22.77	2.973
2	2	sp|P11940|PABP1_HUMAN	PABPC1	70.63	2.9718
2	2	sp|P61204|ARF3_HUMAN	ARF3	20.59	2.9645
2	2	sp|Q9NVI1|FANCI_HUMAN	FANCI	149.23	2.9512
2	2	sp|P00558|PGK1_HUMAN	PGK1	44.59	2.907
2	2	sp|Q8NHW5|RLA0L_HUMAN	RPLP0P6	34.34	2.9017
2	2	sp|P62244|RS15A_HUMAN	RPS15A	14.83	2.8418
2	2	sp|P62191|PRS4_HUMAN	PSMC1	49.15	2.8149
2	2	sp|Q99623|PHB2_HUMAN	PHB2	33.28	2.8078
2	2	sp|O00410|IPO5_HUMAN	IPO5	123.55	2.7963
2	2	sp|Q13619|CUL4A_HUMAN	CUL4A	87.62	2.7793
2	2	sp|P50502|F10A1_HUMAN	ST13	41.31	2.7769
2	2	sp|Q07021|C1QBP_HUMAN	C1QBP	31.34	2.7383
2	2	sp|P62249|RS16_HUMAN	RPS16	16.44	2.7149
2	2	sp|P60842|IF4A1_HUMAN	EIF4A1	46.12	2.6581
2	2	sp|P17812|PYRG1_HUMAN	CTPS1	66.65	2.6572
2	2	sp|Q58FF6|H90B4_HUMAN	HSP90AB4I	58.23	2.6247
2	2	sp|P02786|TFR1_HUMAN	TFRC	84.82	2.5886
2	2	sp|P12004|PCNA_HUMAN	PCNA	28.75	2.5824
2	2	sp|Q9UJS0|CMC2_HUMAN	SLC25A13	74.13	2.5621
2	2	sp|P30153|2AAA_HUMAN	PPP2R1A	65.27	2.5619
2	2	sp|Q96RQ3|MCCA_HUMAN	MCCC1	80.42	2.5503
2	2	sp|O43390|HNRPR_HUMAN	HNRNPR	70.9	2.5398
2	2	sp|O43707|ACTN4_HUMAN	ACTN4	104.79	2.4938
2	2	sp|O60506|HNRPQ_HUMAN	SYNCRIP	69.56	2.4452
2	2	sp|P83731|RL24_HUMAN	RPL24	17.77	2.4373
2	2	sp|Q00325|MPCP_HUMAN	SLC25A3	40.07	2.3783
2	2	sp|P14868|SYDC_HUMAN	DARS	57.1	2.3681
2	2	sp|O43592|XPOT_HUMAN	XPOT	109.89	2.3371
2	2	sp|P63208|SKP1_HUMAN	SKP1	18.65	2.2898
2	2	sp|Q9Y678|COPG1_HUMAN	COPG1	97.66	2.2892
2	2	sp|O14744|ANM5_HUMAN	PRMT5	72.64	2.1921
2	2	sp|P40926|MDHM_HUMAN	MDH2	35.48	2.1377
1	2	sp|P05166|PCCB_HUMAN	PCCB	58.18	4.2221
1	2	sp|P68366|TBA4A_HUMAN	TUBA4A	49.89	3.324
1	2	sp|Q6ZMU5|TRI72_HUMAN	TRIM72	52.7	3.2239
1	2	sp|P55084|ECHB_HUMAN	HADHB	51.26	3.1348
1	2	tr|Q8IWP6|Q8IWP6_HUMAN		49.72	3.0095

1	2	sp|Q9NZD2|GLTP_HUMAN	GLTP	23.83	2.7919
1	2	sp|Q04917|1433F_HUMAN	YWHAH	28.2	2.6282
1	2	sp|P62829|RL23_HUMAN	RPL23	14.86	2.6016
1	2	sp|Q96EK5|KBP_HUMAN	KIF1BP	71.77	2.6015
1	1	sp|Q8NC51|PAIRB_HUMAN	SERBP1	44.94	5.2841
1	1	sp|P43897|EFTS_HUMAN	TSFM	35.37	4.6046
1	1	sp|O00303|EIF3F_HUMAN	EIF3F	37.54	4.5109
1	1	sp|Q13509|TBB3_HUMAN	TUBB3	50.4	4.5009
1	1	sp|Q9BQ67|GRWD1_HUMAN	GRWD1	49.39	4.4201
1	1	sp|P50402|EMD_HUMAN	EMD	28.98	4.3362
1	1	sp|Q9UN86|G3BP2_HUMAN	G3BP2	54.09	4.3215
1	1	sp|P27348|1433T_HUMAN	YWHAQ	27.75	4.2882
1	1	sp|Q66K14|TBC9B_HUMAN	TBC1D9B	140.44	4.252
1	1	sp|P07478|TRY2_HUMAN	PRSS2	26.47	4.1436
1	1	sp|P24752|THIL_HUMAN	ACAT1	45.17	4.0274
1	1	sp|A6NHL2|TBAL3_HUMAN	TUBAL3	49.88	4.0099
1	1	sp|P43686|PRS6B_HUMAN	PSMC4	47.34	3.9712
1	1	sp|Q13765|NACA_HUMAN	NACA	23.37	3.9568
1	1	sp|P07355|ANXA2_HUMAN	ANXA2	38.58	3.8098
1	1	sp|Q13748|TBA3C_HUMAN	TUBA3C	49.93	3.7489
1	1	sp|O95376|ARI2_HUMAN	ARIH2	57.78	3.7446
1	1	sp|P50914|RL14_HUMAN	RPL14	23.42	3.7162
1	1	sp|P62241|RS8_HUMAN	RPS8	24.19	3.7161
1	1	sp|Q15084|PDIA6_HUMAN	PDIA6	48.09	3.6993
1	1	sp|Q9Y285|SYFA_HUMAN	FARSA	57.53	3.6886
1	1	sp|P12268|IMDH2_HUMAN	IMPDH2	55.77	3.6638
1	1	sp|Q5H9R7|PP6R3_HUMAN	PPP6R3	97.61	3.6334
1	1	tr|Q5CAQ4|Q5CAQ4_HUMAN	TRAP1	57.2	3.6039
1	1	sp|P06493|CDK1_HUMAN	CDK1	34.07	3.5527
1	1	sp|Q12905|ILF2_HUMAN	ILF2	43.04	3.5492
1	1	sp|P43307|SSRA_HUMAN	SSR1	32.22	3.5473
1	1	sp|P62333|PRS10_HUMAN	PSMC6	44.15	3.5425
1	1	sp|O43143|DHX15_HUMAN	DHX15	90.88	3.5251
1	1	sp|P08708|RS17_HUMAN	RPS17	15.54	3.479
1	1	sp|P04075|ALDOA_HUMAN	ALDOA	39.4	3.4701
1	1	sp|P60174|TPIS_HUMAN	TPI1	30.77	3.4696
1	1	sp|P62820|RAB1A_HUMAN	RAB1A	22.66	3.4631
1	1	sp|Q14318|FKBP8_HUMAN	FKBP8	44.53	3.4349
1	1	sp|P67775|PP2AA_HUMAN	PPP2CA	35.57	3.4196
1	1	sp|Q15942|ZYX_HUMAN	ZYX	61.24	3.4134
1	1	sp|Q9NXR7|BABA2_HUMAN	BABAM2	43.52	3.3955
1	1	sp|P06744|G6PI_HUMAN	GPI	63.11	3.3654
1	1	sp|Q96QK1|VPS35_HUMAN	VPS35	91.65	3.3407
1	1	sp|P31689|DNJA1_HUMAN	DNAJA1	44.84	3.3199
1	1	sp|P60866|RS20_HUMAN	RPS20	13.36	3.2909
1	1	sp|Q53GQ0|DHB12_HUMAN	HSD17B12	34.3	3.2523
1	1	sp|P27695|APEX1_HUMAN	APEX1	35.53	3.2473

1	1 sp|Q9H9B4|SFXN1_HUMAN	SFXN1	35.6	3.2456
1	1 sp|P51571|SSRD_HUMAN	SSR4	18.99	3.2383
1	1 sp|Q15517|CDSN_HUMAN	CDSN	51.49	3.2357
1	1 tr|A0A024RBS1|A0A024RBS1_	GCN1L1	266.71	3.2296
1	1 sp|P42765|THIM_HUMAN	ACAA2	41.9	3.2168
1	1 sp|Q9NTK5|OLA1_HUMAN	OLA1	44.72	3.2039
1	1 sp|P13929|ENOB_HUMAN	ENO3	46.96	3.1658
1	1 sp|Q9HAV4|XPO5_HUMAN	XPO5	136.22	3.1441
1	1 sp|Q15046|SYK_HUMAN	KARS	68	3.1399
1	1 sp|P49720|PSB3_HUMAN	PSMB3	22.93	3.1236
1	1 sp|O15397|IPO8_HUMAN	IPO8	119.86	3.123
1	1 sp|P10515|ODP2_HUMAN	DLAT	68.95	3.1199
1	1 sp|P55010|IF5_HUMAN	EIF5	49.19	3.0716
1	1 sp|Q01813|PFKAP_HUMAN	PFKP	85.54	3.0658
1	1 sp|Q8N6T3|ARFG1_HUMAN	ARFGAP1	44.64	3.0547
1	1 sp|P47897|SYQ_HUMAN	QARS	87.74	3.0397
1	1 sp|P55209|NP1L1_HUMAN	NAP1L1	45.35	3.0034
1	1 sp|P61026|RAB10_HUMAN	RAB10	22.53	2.9856
1	1 sp|Q15233|NONO_HUMAN	NONO	54.2	2.9855
1	1 sp|Q9BTE6|AASD1_HUMAN	AARSD1	45.45	2.9677
1	1 sp|P31948|STIP1_HUMAN	STIP1	62.6	2.9619
1	1 sp|Q71RC2|LARP4_HUMAN	LARP4	80.55	2.9538
1	1 sp|O00231|PSD11_HUMAN	PSMD11	47.43	2.9303
1	1 sp|P62081|RS7_HUMAN	RPS7	22.11	2.9137
1	1 sp|Q32MZ4|LRRF1_HUMAN	LRRFIP1	89.2	2.9086
1	1 sp|Q01650|LAT1_HUMAN	SLC7A5	54.97	2.8977
1	1 sp|P43246|MSH2_HUMAN	MSH2	104.68	2.8851
1	1 sp|Q9UBB4|ATX10_HUMAN	ATXN10	53.45	2.8832
1	1 sp|P55786|PSA_HUMAN	NPEPPS	103.21	2.875
1	1 sp|Q02978|M2OM_HUMAN	SLC25A11	34.04	2.8604
1	1 sp|P18124|RL7_HUMAN	RPL7	29.21	2.839
1	1 sp|O15523|DDX3Y_HUMAN	DDX3Y	73.11	2.8243
1	1 sp|Q9NZL4|HPBP1_HUMAN	HSPBP1	39.45	2.8238
1	1 sp|P30048|PRDX3_HUMAN	PRDX3	27.68	2.7843
1	1 sp|Q14568|HS902_HUMAN	HSP90AA2I	39.34	2.7811
1	1 sp|O75369|FLNB_HUMAN	FLNB	277.99	2.7615
1	1 sp|P39656|OST48_HUMAN	DDOST	50.77	2.7329
1	1 sp|P54886|P5CS_HUMAN	ALDH18A1	87.25	2.7295
1	1 sp|Q12906|ILF3_HUMAN	ILF3	95.28	2.706
1	1 sp|Q92598|HS105_HUMAN	HSPH1	96.8	2.6941
1	1 sp|Q9NTJ5|SAC1_HUMAN	SACM1L	66.92	2.6854
1	1 sp|Q14651|PLSI_HUMAN	PLS1	70.21	2.6594
1	1 sp|Q15738|NSDHL_HUMAN	NSDHL	41.87	2.6559
1	1 sp|Q13867|BLMH_HUMAN	BLMH	52.53	2.5931
1	1 sp|P18621|RL17_HUMAN	RPL17	21.38	2.5869
1	1 sp|Q92621|NU205_HUMAN	NUP205	227.78	2.5813
1	1 sp|P62269|RS18_HUMAN	RPS18	17.71	2.5651

1	1	sp|P62847|RS24_HUMAN	RPS24	15.41	2.5483
1	1	sp|P17844|DDX5_HUMAN	DDX5	69.1	2.536
1	1	sp|Q15645|PCH2_HUMAN	TRIP13	48.52	2.5308
1	1	sp|Q70Z53|F10C1_HUMAN	FRA10AC1	37.52	2.5201
1	1	sp|Q9NT62|ATG3_HUMAN	ATG3	35.84	2.5177
1	1	sp|Q14C86|GAPD1_HUMAN	GAPVD1	164.88	2.5155
1	1	sp|P40939|ECHA_HUMAN	HADHA	82.95	2.5104
1	1	sp|P43003|EAA1_HUMAN	SLC1A3	59.53	2.4787
1	1	sp|P08865|RSSA_HUMAN	RPSA	32.83	2.4675
1	1	sp|Q8NBS9|TXND5_HUMAN	TXNDC5	47.6	2.4586
1	1	sp|Q15365|PCBP1_HUMAN	PCBP1	37.47	2.4526
1	1	sp|Q13162|PRDX4_HUMAN	PRDX4	30.52	2.4276
1	1	sp|Q00526|CDK3_HUMAN	CDK3	35.02	2.3925
1	1	sp|P62826|RAN_HUMAN	RAN	24.41	2.3713
1	1	sp|Q9Y4L1|HYOU1_HUMAN	HYOU1	111.27	2.3628
1	1	sp|P07741|APT_HUMAN	APRT	19.6	2.3295
1	1	sp|Q9NR82|KCNQ5_HUMAN	KCNQ5	102.12	2.3269
1	1	sp|P56134|ATPK_HUMAN	ATP5J2	10.91	2.3211
1	1	sp|Q9UJZ1|STML2_HUMAN	STOML2	38.51	2.3172
1	1	sp|P36542|ATPG_HUMAN	ATP5C1	32.98	2.3157
1	1	sp|P51784|UBP11_HUMAN	USP11	109.75	2.3097
1	1	sp|Q32P28|P3H1_HUMAN	P3H1	83.34	2.3008
1	1	sp|Q13685|AAMP_HUMAN	AAMP	46.72	2.2907
1	1	tr|S4R464|S4R464_HUMAN	MNX1	3.5	2.2882
1	1	sp|P30837|AL1B1_HUMAN	ALDH1B1	57.17	2.2818
1	1	sp|P05091|ALDH2_HUMAN	ALDH2	56.35	2.2653
1	1	sp|P39023|RL3_HUMAN	RPL3	46.08	2.2637
1	1	sp|P62266|RS23_HUMAN	RPS23	15.8	2.2634
1	1	sp|P62753|RS6_HUMAN	RPS6	28.66	2.2229
1	1	sp|Q9NR30|DDX21_HUMAN	DDX21	87.29	2.2152
1	1	sp|P17858|PFKAL_HUMAN	PFKL	84.96	2.1908
1	1	sp|Q86Y56|DAAF5_HUMAN	DNAAF5	93.46	2.1842
1	1	sp|Q14697|GANAB_HUMAN	GANAB	106.81	2.1642
1	1	sp|P45974|UBP5_HUMAN	USP5	95.73	2.1497
1	1	sp|P32969|RL9_HUMAN	RPL9	21.85	2.0753
1	1	sp|P49321|NASP_HUMAN	NASP	85.19	2.0309

IP6K1 Replicate 1 (IP6K1_1)

Unique peptides	Total peptides	reference	Gene Symbc	MWT(kDa)	AVG
51	58	13085|ACACA_HU	ACACA	265.38	2.8311
24	35	13428|TCOF_HUI	TCOF1	152.02	2.8285
20	178	92551|IP6K1_HUI	IP6K1	50.2	2.3792
20	23	16531|DDB1_HUI	DDB1	126.89	2.5498
20	22	93008|USP9X_HU	USP9X	292.09	2.7539
19	27	38646|GRP75_HU	HSPA9	73.63	3.0166
19	21	P11498|PYC_HUM	PC	129.55	3.0913
18	20	40939|ECHA_HUI	HADHA	82.95	3.0312
15	22	43865|SAHH2_HU	AHCYL1	58.91	3.1239
14	17	68371|TBB4B_HU	TUBB4B	49.8	3.1339
14	14	05165|PCCA_HUI	PCCA	80.01	3.286
14	14	11021|GRP78_HU	HSPA5	72.29	2.7561
13	17	9BQE3|TBA1C_HU	TUBA1C	49.86	2.991
12	15	11142|HSP7C_HU	HSPA8	70.85	3.0611
12	14	96RQ3|MCCA_HU	MCCC1	80.42	3.3057
12	14	14139|UBE4A_HU	UBE4A	122.48	3.2345
12	14	55084|ECHB_HUI	HADHB	51.26	2.8372
12	12	09874|PARP1_HU	PARP1	113.01	2.8889
10	11	P23396|RS3_HUM	RPS3	26.67	2.7332
10	10	9HCC0|MCCB_HU	MCCC2	61.29	2.4919
9	11	42704|LPPRC_HU	LRPPRC	157.81	3.399
9	9	05166|PCCB_HUI	PCCB	58.18	3.3703
9	9	12236|ADT3_HUI	SLC25A6	32.85	2.7763
9	9	00203|AP3B1_HU	AP3B1	121.24	2.7382
8	11	P62805|H4_HUM	HIST1H4A	11.36	2.6439
8	9	2272|HNRPM_HU	HNRNPM	77.46	2.8985
7	45	GFP_Aequorea	FP_Aequore	26.87	2.833
7	9	68400|CSK21_HU	CSNK2A1	45.11	3.1171
6	9	33778|H2B1B_HU	HIST1H2BB	13.94	2.3831
6	6	34931|HS71L_HU	HSPA1L	70.33	3.1216
5	8	62736|ACTA_HUI	ACTA2	41.98	2.121
5	6	08670|VIME_HUI	VIM	53.62	2.6891
5	5	0DMV9|HS71B_HU	HSPA1B	70.01	2.8429
4	7	96KK5|H2A1H_HU	HIST1H2AH	13.9	2.8477
4	6	07437|TBB5_HUI	TUBB	49.64	3.4113
4	5	07021|C1QBP_HU	C1QBP	31.34	3.0024
4	5	014654|IRS4_HUN	IRS4	133.68	2.6161
4	5	62987|RL40_HUN	UBA52	14.72	2.3952
4	4	19338|NUCL_HUI	NCL	76.57	2.7433
4	4	51784|UBP11_HU	USP11	109.75	2.7075
4	4	22626|ROA2_HUI	HNRNPA2B1	37.41	2.5814
4	4	13010|XRCC5_HU	XRCC5	82.65	2.5541
4	4	00325|MPCP_HU	SLC25A3	40.07	2.4041
4	4	9Y265|RUVB1_HU	RUVBL1	50.2	2.3999
4	4	P46781|RS9_HUM	RPS9	22.58	2.3368

4	4	P61247|RS3A_HUN	RPS3A	29.93	1.9216
3	4	P25705|ATPA_HUN	ATP5A1	59.71	3.0536
3	4	Q02878|RL6_HUN	RPL6	32.71	2.7403
3	4	P62081|RS7_HUN	RPS7	22.11	2.6948
3	4	9NR30|DDX21_HU	DDX21	87.29	2.6234
3	3	11182|ODB2_HUI	DBT	53.45	3.5209
3	3	P62241|RS8_HUN	RPS8	24.19	3.5012
3	3	00839|HNRPU_HU	HNRNPU	90.53	3.4428
3	3	P62847|RS24_HUN	RPS24	15.41	3.3447
3	3	Q12905|ILF2_HUN	ILF2	43.04	3.2573
3	3	P62829|RL23_HUN	RPL23	14.86	3.1553
3	3	P06748|NPM_HUN	NPM1	32.55	3.1091
3	3	P15880|RS2_HUN	RPS2	31.3	3.054
3	3	96HN2|SAHH3_HU	AHCYL2	66.68	3.0206
3	3	19784|CSK22_HU	CSNK2A2	41.19	2.8858
3	3	P17987|TCPA_HUN	TCP1	60.31	2.862
3	3	P62263|RS14_HUN	RPS14	16.26	2.8586
3	3	54652|HSP72_HU	HSPA2	69.98	2.8578
3	3	Q08211|DHX9_HUI	DHX9	140.87	2.8494
3	3	32119|PRDX2_HU	PRDX2	21.88	2.8316
3	3	07910|HNRPC_HU	HNRNPC	33.65	2.8268
3	3	9BVA1|TBB2B_HU	TUBB2B	49.92	2.821
3	3	15355|PPM1G_HU	PPM1G	59.23	2.7632
3	3	P16403|H12_HUN	HIST1H1C	21.35	2.5711
3	3	P63261|ACTG_HUI	ACTG1	41.77	2.533
3	3	Q13263|TIF1B_HUI	TRIM28	88.49	2.4862
3	3	Q14257|RCN2_HUI	RCN2	36.85	2.2068
3	3	P17480|UBF1_HUN	UBTF	89.35	2.1825
2	3	67809|YBOX1_HU	YBX1	35.9	4.0282
2	3	96017|CHK2_HUI	CHEK2	60.88	2.9875
2	3	Q71DI3|H32_HUN	HIST2H3A	15.38	1.836
2	2	07900|HS90A_HU	HSP90AA1	84.61	3.9353
2	2	55795|HNRH2_HU	HNRNPH2	49.23	3.8136
2	2	32P51|RA1L2_HU	HNRNPA1L2	34.2	3.7559
2	2	P05141|ADT2_HUI	SLC25A5	32.83	3.4701
2	2	Q9UK99|FBX3_HUI	FBXO3	54.53	3.458
2	2	12956|XRCC6_HU	XRCC6	69.8	3.4316
2	2	62244|RS15A_HU	RPS15A	14.83	3.3024
2	2	9NS91|RAD18_HU	RAD18	56.19	3.2381
2	2	P78371|TCPB_HUN	CCT2	57.45	3.1488
2	2	14983|AT2A1_HU	ATP2A1	110.18	3.1234
2	2	43243|MATR3_HU	MATR3	94.56	3.0537
2	2	28331|NDUS1_HU	NDUFS1	79.42	3.0469
2	2	9Y230|RUVB2_HU	RUVBL2	51.12	3.0126
2	2	Q9Y3D9|RT23_HUI	MRPS23	21.76	3.0042
2	2	P62913|RL11_HUN	RPL11	20.24	2.9843
2	2	2RXH8|HNRC2_HU	HNRNPCL2	32.05	2.9217

2	2	²62249|RS16_HUI	RPS16	16.44	2.9086
2	2	²82933|RT09_HUI	MRPS9	45.81	2.7675
2	2	²83731|RL24_HUI	RPL24	17.77	2.7641
2	2	²62701|RS4X_HUI	RPS4X	29.58	2.7394
2	2	²62269|RS18_HUI	RPS18	17.71	2.7258
2	2	42167|LAP2B_HU	TMPO	50.64	2.7013
2	2	Q13309|SKP2_HUI	SKP2	47.73	2.6919
2	2	²39019|RS19_HUI	RPS19	16.05	2.6715
2	2	68104|EF1A1_HU	EEF1A1	50.11	2.6602
2	2	Q5T4S7|UBR4_HUI	UBR4	573.48	2.6586
2	2	²82650|RT22_HUI	MRPS22	41.25	2.6555
2	2	O14773|TPP1_HUI	TPP1	61.21	2.6066
2	2	9P035|HACD3_HU	HACD3	43.13	2.5622
2	2	62258|1433E_HU	YWHAE	29.16	2.4911
2	2	²62899|RL31_HUI	RPL31	14.45	2.4818
2	2	²40227|TCPZ_HUI	CCT6A	57.99	2.4455
2	2	15233|NONO_HU	NONO	54.2	2.3891
2	2	²27708|PYR1_HUI	CAD	242.83	2.2742
2	2	06830|PRDX1_HU	PRDX1	22.1	2.2293
2	2	93079|H2B1H_HU	HIST1H2BH	13.88	2.2008
2	2	P62753|RS6_HUI	RPS6	28.66	2.0901
2	2	²8340|DPOD1_HU	POLD1	123.55	2.0036
1	23	9UHH9|IP6K2_HU	IP6K2	49.15	3.3024
1	3	6ZMU5|TRI72_HU	TRIM72	52.7	2.9376
1	2	3ZCQ8|TIM50_HU	TIMM50	39.62	4.7828
1	2	O0567|NOP56_HU	NOP56	66.01	3.8679
1	2	9H3K6|BOLA2_HU	BOLA2	10.11	3.6197
1	2	²62854|RS26_HUI	RPS26	13.01	2.8407
1	2	60506|HNRPQ_HU	SYNCRIP	69.56	2.6812
1	2	Q9NZ01|TECR_HUI	TECR	36.01	2.1829
1	1	13620|CUL4B_HU	CUL4B	103.92	4.3665
1	1	34932|HSP74_HU	HSPA4	94.27	4.3332
1	1	P49327|FAS_HUI	FASN	273.25	4.2924
1	1	Q9BYN8|RT26_HUI	MRPS26	24.2	4.281
1	1	38159|RBMX_HUI	RBMX	42.31	4.2325
1	1	Q96JC9|EAF1_HUI	EAF1	29.02	4.0007
1	1	43390|HNRPR_HU	HNRNPR	70.9	3.9585
1	1	7ETY2|E7ETY2_HU	TCOF1	152.21	3.8642
1	1	O75531|BAF_HUI	BANF1	10.05	3.8253
1	1	²63208|SKP1_HUI	SKP1	18.65	3.8123
1	1	O43164|PJA2_HUI	PJA2	78.17	3.755
1	1	²62266|RS23_HUI	RPS23	15.8	3.7099
1	1	²50990|TCPQ_HUI	CCT8	59.58	3.616
1	1	55209|NP1L1_HU	NAP1L1	45.35	3.605
1	1	²35268|RL22_HUI	RPL22	14.78	3.5737
1	1	²10809|CH60_HUI	HSPD1	61.02	3.5211
1	1	²08708|RS17_HUI	RPS17	15.54	3.5005

1	1	?42677|RS27_HUM	RPS27	9.45	3.4598
1	1	5TAQ9|DCAF8_HU	DCAF8	66.81	3.4592
1	1	Q9Y625|GPC6_HUI	GPC6	62.69	3.4568
1	1	P12273|PIP_HUM	PIP	16.56	3.3864
1	1	08238|HS90B_HU	HSP90AB1	83.21	3.3749
1	1	96EY7|PTCD3_HU	PTCD3	78.5	3.3587
1	1	P18124|RL7_HUM	RPL7	29.21	3.3334
1	1	9NQ39|RS10L_HU	RPS10P5	20.11	3.3262
1	1	13557|KCC2D_HU	CAMK2D	56.33	3.3132
1	1	Q5UIP0|RIF1_HUM	RIF1	274.29	3.2757
1	1	31689|DNJA1_HU	DNAJA1	44.84	3.2308
1	1	07355|ANXA2_HU	ANXA2	38.58	3.2294
1	1	9Y5V3|MAGD1_HU	MAGED1	86.11	3.2132
1	1	60762|DPM1_HU	DPM1	29.62	3.1489
1	1	Q14974|IMB1_HUI	KPNB1	97.11	3.1381
1	1	Q12906|ILF3_HUM	ILF3	95.28	3.0852
1	1	16615|AT2A2_HU	ATP2A2	114.68	3.0851
1	1	Q9BUF5|TBB6_HUI	TUBB6	49.82	3.0811
1	1	.15393|SF3B3_HU	SF3B3	135.49	3.0683
1	1	?26373|RL13_HUM	RPL13	24.25	3.0562
1	1	?50402|EMD_HUM	EMD	28.98	3.019
1	1	WUK0|PTPM1_HU	PTPMT1	22.83	3.0096
1	1	Q02539|H11_HUM	HIST1H1A	21.83	3.0028
1	1	92841|DDX17_HU	DDX17	80.22	2.9701
1	1	04792|HSPB1_HU	HSPB1	22.77	2.8971
1	1	52597|HNRPF_HU	HNRNPF	45.64	2.8735
1	1	Q5JNZ5|RS26L_HU	RPS26P11	12.99	2.8271
1	1	?06493|CDK1_HUM	CDK1	34.07	2.8097
1	1	14103|HNRPD_HU	HNRNPD	38.41	2.8019
1	1	?62851|RS25_HUM	RPS25	13.73	2.7803
1	1	02545|LMNA_HU	LMNA	74.09	2.7423
1	1	Q9Y295|DRG1_HUI	DRG1	40.52	2.7297
1	1	P08195|4F2_HUM	SLC3A2	67.95	2.6989
1	1	P13489|RINI_HUM	RNH1	49.94	2.6769
1	1	P62917|RL8_HUM	RPL8	28.01	2.6347
1	1	Q9GZT3|SLIRP_HUI	SLIRP	12.34	2.6208
1	1	Q15517|CDSN_HUI	CDSN	51.49	2.613
1	1	58FF7|H90B3_HU	HSP90AB3P	68.28	2.601
1	1	4R2I7|A0A024R2I7	RAD18	56.16	2.5976
1	1	?35030|TRY3_HUM	PRSS3	32.51	2.5971
1	1	0PJA5|A0PJA5_HU	GCC2	48.72	2.5468
1	1	14635|CCNB1_HU	CCNB1	48.31	2.5408
1	1	92572|AP3S1_HU	AP3S1	21.72	2.5306
1	1	Q15758|AAAT_HUI	SLC1A5	56.56	2.5233
1	1	6ZRS2|SRCAP_HU	SRCAP	343.34	2.5024
1	1	?22314|UBA1_HUI	UBA1	117.77	2.4537
1	1	?17844|DDX5_HUI	DDX5	69.1	2.4263

1	1	11940|PABP1_HU	PABPC1	70.63	2.424
1	1	9C0C9|UBE2O_HU	UBE2O	141.21	2.4182
1	1	P13639|EF2_HUM	EEF2	95.28	2.3924
1	1	P62273|RS29_HUM	RPS29	6.67	2.2889
1	1	Q9Y3D3|RT16_HU	MRPS16	15.34	2.283
1	1	9NZL4|HPBP1_HU	HSPBP1	39.45	2.2132
1	1	53367|ARFP1_HU	ARFIP1	41.71	2.1986
1	1	P30050|RL12_HUM	RPL12	17.81	2.1794
1	1	62750|RL23A_HU	RPL23A	17.68	2.1793
1	1	01469|FABP5_HU	FABP5	15.15	2.1759
1	1	5DRA6|H2B2D_HU	HIST2H2BD	18.01	2.1692
1	1	30837|AL1B1_HU	ALDH1B1	57.17	2.1672
1	1	Q01844|EWS_HUM	EWSR1	68.44	2.1248
1	1	P61353|RL27_HUM	RPL27	15.79	2.1072
1	1	95831|AIFM1_HU	AIFM1	66.86	2.061
1	1	13555|KCC2G_HU	CAMK2G	62.57	2.0549
1	1	P47914|RL29_HUM	RPL29	17.74	2.0534
1	1	60296|TRAK2_HU	TRAK2	101.36	2.0394
1	1	51991|ROA3_HUI	HNRNPA3	39.57	2.0253
1	1	DX69|B4DX69_HU	9|B4DX69_	21.81	1.9208

IP6K1 Replicate 2 (IP6K1_2)

Unique	Total	reference	Gene Symb	MWT(kDa)	AVG
40	50	sp|P42704|LPPRC_HUMAN	LRPPRC	157.81	2.9365
28	38	sp|Q93008|USP9X_HUMAN	USP9X	292.09	2.6428
21	194	sp|Q92551|IP6K1_HUMAN	IP6K1	50.2	2.4405
21	29	sp|P49327|FAS_HUMAN	FASN	273.25	3.0458
17	31	sp|P68371|TBB4B_HUMAN	TUBB4B	49.8	2.8543
17	29	sp|P38646|GRP75_HUMAN	HSPA9	73.63	3.0057
17	19	sp|P78527|PRKDC_HUMAN	PRKDC	468.79	2.8943
16	21	sp|Q14139|UBE4A_HUMAN	UBE4A	122.48	3.2288
15	18	sp|Q00839|HNRPU_HUMAN	HNRNPU	90.53	2.9269
15	18	sp|P19338|NUCL_HUMAN	NCL	76.57	2.7763
15	17	sp|Q13428|TCOF_HUMAN	TCOF1	152.02	2.7817
15	16	sp|P40939|ECHA_HUMAN	HADHA	82.95	3.3487
15	16	sp|P14618|KPYM_HUMAN	PKM	57.9	3.2409
14	25	sp|Q13263|TIF1B_HUMAN	TRIM28	88.49	3.0056
14	21	sp|P23396|RS3_HUMAN	RPS3	26.67	2.7318
14	17	sp|Q08211|DHX9_HUMAN	DHX9	140.87	2.6447
14	15	sp|P50990|TCPQ_HUMAN	CCT8	59.58	3.081
14	15	sp|Q12906|ILF3_HUMAN	ILF3	95.28	2.6334
13	20	sp|P11142|HSP7C_HUMAN	HSPA8	70.85	3.258
13	14	sp|P17987|TCPA_HUMAN	TCP1	60.31	2.847
11	20	sp|Q9BQE3|TBA1C_HUMAN	TUBA1C	49.86	2.8473
11	14	sp|Q9C0C9|UBE2O_HUMAN	UBE2O	141.21	2.8813
11	14	sp|P07900|HS90A_HUMAN	HSP90AA1	84.61	2.6369
11	14	sp|Q16531|DDB1_HUMAN	DDB1	126.89	2.3335
11	12	sp|Q9P2J5|SYLC_HUMAN	LARS	134.38	3.3486
11	12	sp|P78371|TCPB_HUMAN	CCT2	57.45	3.3178
11	12	sp|O43865|SAHH2_HUMAN	AHCYL1	58.91	2.8468
11	12	sp|P61247|RS3A_HUMAN	RPS3A	29.93	2.4129
11	11	sp|P07814|SYEP_HUMAN	EPRS	170.48	3.1093
11	11	sp|Q9Y230|RUVB2_HUMAN	RUVBL2	51.12	2.8735
10	18	sp|Q12905|ILF2_HUMAN	ILF2	43.04	3.3765
10	14	sp|O43390|HNRPR_HUMAN	HNRNPR	70.9	2.9438
10	12	sp|P06576|ATPB_HUMAN	ATP5B	56.52	3.0102
10	11	sp|P11021|GRP78_HUMAN	HSPA5	72.29	3.215
10	10	sp|P10809|CH60_HUMAN	HSPD1	61.02	3.1811
9	17	sp|P62258|1433E_HUMAN	YWHAE	29.16	2.6606
9	14	sp|O60506|HNRPQ_HUMAN	SYNCRIP	69.56	2.9531
9	12	sp|P46781|RS9_HUMAN	RPS9	22.58	2.4056
9	11	sp|P50991|TCPD_HUMAN	CCT4	57.89	2.6791
9	10	sp|P22061|PIMT_HUMAN	PCMT1	24.62	2.914
9	10	sp|Q92616|GCN1_HUMAN	GCN1	292.57	2.8695
9	10	sp|P52272|HNRPM_HUMAN	HNRNPM	77.46	2.5464
9	9	sp|P55084|ECHB_HUMAN	HADHB	51.26	3.0726
8	21	sp|O15355|PPM1G_HUMAN	PPM1G	59.23	2.9505
8	13	sp|P34931|HS71L_HUMAN	HSPA1L	70.33	2.9694

8	11	sp|P82650|RT22_HUMAN	MRPS22	41.25	2.6005
8	10	sp|P14868|SYDC_HUMAN	DARS	57.1	2.8487
8	10	sp|P49368|TCPG_HUMAN	CCT3	60.5	2.7656
8	9	sp|P0DMV9|HS71B_HUMAN	HSPA1B	70.01	2.9075
8	9	sp|P14625|ENPL_HUMAN	HSP90B1	92.41	2.8928
8	9	sp|P48643|TCPE_HUMAN	CCT5	59.63	2.8339
8	9	sp|Q92552|RT27_HUMAN	MRPS27	47.58	2.824
8	9	sp|Q13616|CUL1_HUMAN	CUL1	89.62	2.6864
8	8	sp|P12956|XRCC6_HUMAN	XRCC6	69.8	3.1042
8	8	sp|Q96EY7|PTCD3_HUMAN	PTCD3	78.5	3.054
8	8	sp|P25705|ATPA_HUMAN	ATP5A1	59.71	2.9963
8	8	sp|P68400|CSK21_HUMAN	CSNK2A1	45.11	2.8639
7	17	sp|P12236|ADT3_HUMAN	SLC25A6	32.85	2.8273
7	13	sp|P63244|RACK1_HUMAN	RACK1	35.05	2.9133
7	12	sp|Q99714|HCD2_HUMAN	HSD17B10	26.91	3.1089
7	11	sp|P40227|TCPZ_HUMAN	CCT6A	57.99	2.8225
7	11	sp|P62736|ACTA_HUMAN	ACTA2	41.98	2.4763
7	10	sp|P06493|CDK1_HUMAN	CDK1	34.07	3.2778
7	10	sp|P61978|HNRPK_HUMAN	HNRNPK	50.94	3.2522
7	10	sp|P68104|EF1A1_HUMAN	EEF1A1	50.11	2.7928
7	9	sp|P22695|QCR2_HUMAN	UQCRC2	48.41	2.6728
7	8	sp|Q93009|UBP7_HUMAN	USP7	128.22	2.5724
7	8	sp|P17844|DDX5_HUMAN	DDX5	69.1	2.5545
7	7	sp|P62263|RS14_HUMAN	RPS14	16.26	3.4422
7	7	sp|Q9NZI8|IF2B1_HUMAN	IGF2BP1	63.44	3.2026
7	7	sp|P51784|UBP11_HUMAN	USP11	109.75	2.8099
7	7	sp|Q9UJS0|CMC2_HUMAN	SLC25A13	74.13	2.7481
7	7	sp|P11940|PABP1_HUMAN	PABPC1	70.63	2.6241
7	7	sp|O00203|AP3B1_HUMAN	AP3B1	121.24	2.5936
6	12	sp|P39019|RS19_HUMAN	RPS19	16.05	2.651
6	9	sp|P13489|RINI_HUMAN	RNH1	49.94	3.1435
6	9	sp|Q07021|C1QBP_HUMAN	C1QBP	31.34	2.9295
6	8	sp|Q99832|TCPH_HUMAN	CCT7	59.33	3.571
6	8	sp|Q9Y265|RUVB1_HUMAN	RUVBL1	50.2	3.2996
6	8	sp|P62269|RS18_HUMAN	RPS18	17.71	2.6389
6	8	sp|P41252|SYIC_HUMAN	IARS	144.41	2.565
6	8	sp|P62249|RS16_HUMAN	RPS16	16.44	2.5595
6	7	sp|Q9NR30|DDX21_HUMAN	DDX21	87.29	2.9949
6	7	sp|P54136|SYRC_HUMAN	RARS	75.33	2.7981
6	7	sp|Q13085|ACACA_HUMAN	ACACA	265.38	2.6053
6	7	sp|Q14103|HNRPD_HUMAN	HNRNPD	38.41	2.5668
6	6	sp|Q13620|CUL4B_HUMAN	CUL4B	103.92	3.1675
6	6	sp|P05023|AT1A1_HUMAN	ATP1A1	112.82	3.1572
6	6	sp|P34897|GLYM_HUMAN	SHMT2	55.96	3.1381
6	6	sp|Q86VP6|CAND1_HUMAN	CAND1	136.29	3.1051
6	6	sp|Q9Y2L1|RRP44_HUMAN	DIS3	108.93	3.0165
6	6	sp|P11586|C1TC_HUMAN	MTHFD1	101.5	2.7368

6	6	sp|P28340|DPOD1_HUMAN	POLD1	123.55	2.6514
6	6	sp|Q9Y2R9|RT07_HUMAN	MRPS7	28.12	2.5772
6	6	sp|O75534|CSDE1_HUMAN	CSDE1	88.83	2.1278
5	10	GFP_Aequorea		26.87	2.8348
5	9	sp|P62701|RS4X_HUMAN	RPS4X	29.58	3.0704
5	8	sp|P13010|XRCC5_HUMAN	XRCC5	82.65	2.6966
5	7	sp|Q01105|SET_HUMAN	SET	33.47	3.5381
5	7	sp|P52292|IMA1_HUMAN	KPNA2	57.83	2.9813
5	7	sp|P56192|SYMC_HUMAN	MARS	101.05	2.8941
5	7	sp|P15880|RS2_HUMAN	RPS2	31.3	2.8006
5	7	sp|P25205|MCM3_HUMAN	MCM3	90.92	2.7734
5	6	sp|Q9BUF5|TBB6_HUMAN	TUBB6	49.82	3.2856
5	6	sp|Q5JTZ9|SYAM_HUMAN	AARS2	107.27	2.9569
5	6	sp|P08243|ASNS_HUMAN	ASNS	64.33	2.7998
5	6	sp|Q96QK1|VPS35_HUMAN	VPS35	91.65	2.5477
5	5	sp|Q92665|RT31_HUMAN	MRPS31	45.29	3.3366
5	5	sp|Q14974|IMB1_HUMAN	KPNB1	97.11	3.2751
5	5	sp|Q92841|DDX17_HUMAN	DDX17	80.22	3.2511
5	5	sp|O15523|DDX3Y_HUMAN	DDX3Y	73.11	3.1187
5	5	sp|P54886|P5CS_HUMAN	ALDH18A1	87.25	3.0062
5	5	sp|P49736|MCM2_HUMAN	MCM2	101.83	2.9184
5	5	sp|Q9BY44|EIF2A_HUMAN	EIF2A	64.95	2.9071
5	5	sp|P06733|ENOA_HUMAN	ENO1	47.14	2.8197
5	5	sp|P54652|HSP72_HUMAN	HSPA2	69.98	2.8059
5	5	sp|Q99729|ROAA_HUMAN	HNRNPAB	36.2	2.7813
5	5	sp|Q9Y3I0|RTCB_HUMAN	RTCB	55.17	2.7061
5	5	sp|Q7L2E3|DHX30_HUMAN	DHX30	133.85	2.6419
5	5	sp|P13639|EF2_HUMAN	EEF2	95.28	2.5659
5	5	sp|P47897|SYQ_HUMAN	QARS	87.74	2.4097
5	5	sp|Q7L0Y3|MRRP1_HUMAN	TRMT10C	47.32	2.3886
5	5	sp|Q00325|MPCP_HUMAN	SLC25A3	40.07	2.3807
5	5	sp|P62750|RL23A_HUMAN	RPL23A	17.68	2.2575
5	5	sp|P36542|ATPG_HUMAN	ATP5C1	32.98	2.173
5	5	sp|P07195|LDHB_HUMAN	LDHB	36.62	2.137
4	9	sp|Q92688|AN32B_HUMAN	ANP32B	28.77	2.4812
4	8	sp|Q9BVA1|TBB2B_HUMAN	TUBB2B	49.92	3.1218
4	7	sp|Q99873|ANM1_HUMAN	PRMT1	41.49	2.9368
4	7	sp|Q58FF7|H90B3_HUMAN	HSP90AB3I	68.28	2.541
4	6	sp|P22314|UBA1_HUMAN	UBA1	117.77	3.6063
4	6	sp|P08708|RS17_HUMAN	RPS17	15.54	3.2373
4	6	sp|Q9BVP2|GNL3_HUMAN	GNL3	61.95	2.8659
4	6	sp|P39687|AN32A_HUMAN	ANP32A	28.57	2.8197
4	6	sp|P09651|ROA1_HUMAN	HNRNPA1	38.72	2.7195
4	5	sp|P28331|NDUS1_HUMAN	NDUFS1	79.42	4.3799
4	5	sp|Q13283|G3BP1_HUMAN	G3BP1	52.13	3.7922
4	5	sp|P34932|HSP74_HUMAN	HSPA4	94.27	3.4894
4	5	sp|P12277|KCRB_HUMAN	CKB	42.62	3.2424

4	5	sp|Q15084|PDIA6_HUMAN	PDIA6	48.09	3.1196
4	5	sp|P08238|HS90B_HUMAN	HSP90AB1	83.21	2.93
4	5	sp|P62899|RL31_HUMAN	RPL31	14.45	2.7901
4	5	sp|P62851|RS25_HUMAN	RPS25	13.73	2.7503
4	5	sp|P33992|MCM5_HUMAN	MCM5	82.23	2.6745
4	5	sp|Q9Y399|RT02_HUMAN	MRPS2	33.23	2.1609
4	4	sp|O00425|IF2B3_HUMAN	IGF2BP3	63.67	3.6345
4	4	sp|P16989|YBOX3_HUMAN	YBX3	40.07	3.5924
4	4	sp|P63261|ACTG_HUMAN	ACTG1	41.77	3.1981
4	4	sp|Q9UK99|FBX3_HUMAN	FBXO3	54.53	3.1899
4	4	sp|Q9UHX1|PUF60_HUMAN	PUF60	59.84	3.0064
4	4	sp|O95831|AIFM1_HUMAN	AIFM1	66.86	2.9749
4	4	sp|Q9Y3D9|RT23_HUMAN	MRPS23	21.76	2.9631
4	4	sp|Q15020|SART3_HUMAN	SART3	109.87	2.9522
4	4	sp|P62241|RS8_HUMAN	RPS8	24.19	2.9472
4	4	sp|O43175|SERA_HUMAN	PHGDH	56.61	2.9385
4	4	sp|P78347|GTF2I_HUMAN	GTF2I	112.35	2.8778
4	4	sp|P62987|RL40_HUMAN	UBA52	14.72	2.8597
4	4	sp|P43490|NAMPT_HUMAN	NAMPT	55.49	2.8582
4	4	sp|P43246|MSH2_HUMAN	MSH2	104.68	2.7024
4	4	sp|Q15942|ZYX_HUMAN	ZYX	61.24	2.6881
4	4	sp|P22626|ROA2_HUMAN	HNRNPA2B	37.41	2.6517
4	4	sp|P26641|EF1G_HUMAN	EEF1G	50.09	2.6144
4	4	sp|P17812|PYRG1_HUMAN	CTPS1	66.65	2.5088
4	4	sp|P82673|RT35_HUMAN	MRPS35	36.82	2.5058
4	4	sp|P60842|IF4A1_HUMAN	EIF4A1	46.12	2.5009
4	4	sp|P35998|PRS7_HUMAN	PSMC2	48.6	2.4787
4	4	sp|P62244|RS15A_HUMAN	RPS15A	14.83	2.2932
3	7	sp|P62277|RS13_HUMAN	RPS13	17.21	2.825
3	6	sp|P07437|TBB5_HUMAN	TUBB	49.64	3.0376
3	5	sp|Q9BTT0|AN32E_HUMAN	ANP32E	30.67	3.765
3	5	sp|P67809|YBOX1_HUMAN	YBX1	35.9	3.1469
3	5	sp|Q00341|VIGLN_HUMAN	HDLBP	141.37	3.1218
3	5	sp|P82664|RT10_HUMAN	MRPS10	22.99	3.0921
3	5	sp|P05388|RLA0_HUMAN	RPLP0	34.25	3.0424
3	5	sp|Q14694|UBP10_HUMAN	USP10	87.08	2.5462
3	4	sp|Q8NC51|PAIRB_HUMAN	SERBP1	44.94	3.6605
3	4	sp|Q14318|FKBP8_HUMAN	FKBP8	44.53	3.3893
3	4	sp|O75489|NDUS3_HUMAN	NDUFS3	30.22	3.2659
3	4	sp|P55209|NP1L1_HUMAN	NAP1L1	45.35	3.1925
3	4	sp|Q9BZE1|RM37_HUMAN	MRPL37	48.09	3.0412
3	4	sp|Q14240|IF4A2_HUMAN	EIF4A2	46.37	3.0353
3	4	sp|Q8NHW5|RLA0L_HUMAN	RPLP0P6	34.34	2.945
3	4	sp|P00338|LDHA_HUMAN	LDHA	36.67	2.8588
3	4	sp|Q6L8Q7|PDE12_HUMAN	PDE12	67.31	2.8142
3	4	sp|P82912|RT11_HUMAN	MRPS11	20.6	2.8088
3	4	sp|P27708|PYR1_HUMAN	CAD	242.83	2.7651

3	4	sp|P05455|LA_HUMAN	SSB	46.81	2.3524
3	4	sp|P04843|RPN1_HUMAN	RPN1	68.53	2.3182
3	4	sp|P18621|RL17_HUMAN	RPL17	21.38	2.2725
3	3	sp|Q12931|TRAP1_HUMAN	TRAP1	80.06	3.6077
3	3	sp|P60866|RS20_HUMAN	RPS20	13.36	3.3698
3	3	sp|P51398|RT29_HUMAN	DAP3	45.54	3.2993
3	3	sp|P82933|RT09_HUMAN	MRPS9	45.81	3.1593
3	3	sp|P40926|MDHM_HUMAN	MDH2	35.48	3.1125
3	3	sp|Q9Y295|DRG1_HUMAN	DRG1	40.52	3.1094
3	3	sp|P07737|PROF1_HUMAN	PFN1	15.04	3.0854
3	3	sp|P62753|RS6_HUMAN	RPS6	28.66	3.0672
3	3	sp|O00571|DDX3X_HUMAN	DDX3X	73.2	3.0451
3	3	sp|Q92499|DDX1_HUMAN	DDX1	82.38	3.0409
3	3	sp|Q14566|MCM6_HUMAN	MCM6	92.83	2.9905
3	3	sp|Q9Y383|LC7L2_HUMAN	LUC7L2	46.49	2.9787
3	3	sp|P16615|AT2A2_HUMAN	ATP2A2	114.68	2.9231
3	3	sp|P62829|RL23_HUMAN	RPL23	14.86	2.9194
3	3	sp|Q9P015|RM15_HUMAN	MRPL15	33.4	2.9189
3	3	sp|P14635|CCNB1_HUMAN	CCNB1	48.31	2.8957
3	3	sp|P53396|ACLY_HUMAN	ACLY	120.76	2.8822
3	3	sp|P08195|4F2_HUMAN	SLC3A2	67.95	2.8615
3	3	sp|P49411|EFTU_HUMAN	TUFM	49.51	2.8377
3	3	sp|Q9Y2Q9|RT28_HUMAN	MRPS28	20.83	2.829
3	3	sp|Q9H9B4|SFXN1_HUMAN	SFXN1	35.6	2.7767
3	3	sp|O14980|XPO1_HUMAN	XPO1	123.31	2.7376
3	3	sp|P04844|RPN2_HUMAN	RPN2	69.24	2.7146
3	3	sp|Q13200|PSMD2_HUMAN	PSMD2	100.14	2.7023
3	3	sp|P39748|FEN1_HUMAN	FEN1	42.57	2.6704
3	3	sp|Q96CX2|KCD12_HUMAN	KCTD12	35.68	2.6334
3	3	sp|Q6DKJ4|NXN_HUMAN	NXN	48.36	2.6327
3	3	sp|Q15046|SYK_HUMAN	KARS	68	2.6205
3	3	sp|P26373|RL13_HUMAN	RPL13	24.25	2.6169
3	3	sp|P62081|RS7_HUMAN	RPS7	22.11	2.5516
3	3	sp|Q96EB6|SIR1_HUMAN	SIRT1	81.63	2.5424
3	3	sp|O00303|EIF3F_HUMAN	EIF3F	37.54	2.4623
3	3	sp|Q9BXS5|AP1M1_HUMAN	AP1M1	48.56	2.4218
3	3	sp|Q9H0I2|ENKD1_HUMAN	ENKD1	38.74	2.3823
3	3	sp|P14678|RSMB_HUMAN	SNRPB	24.59	2.3661
3	3	sp|Q13619|CUL4A_HUMAN	CUL4A	87.62	2.3642
3	3	sp|Q58FF6|H90B4_HUMAN	HSP90AB4I	58.23	2.3549
3	3	sp|P67870|CSK2B_HUMAN	CSNK2B	24.93	2.3276
3	3	sp|P32119|PRDX2_HUMAN	PRDX2	21.88	2.3271
3	3	sp|P35232|PHB_HUMAN	PHB	29.79	2.2507
3	3	sp|Q9Y4B6|DCAF1_HUMAN	DCAF1	168.9	2.0187
2	4	sp|P05141|ADT2_HUMAN	SLC25A5	32.83	3.641
2	4	sp|Q96S59|RANB9_HUMAN	RANBP9	77.8	3.3495
2	4	sp|P82675|RT05_HUMAN	MRPS5	47.98	3.1353

2	4	sp|O60884|DNJA2_HUMAN	DNAJA2	45.72	2.9773
2	4	sp|Q96SB4|SRPK1_HUMAN	SRPK1	74.28	2.9151
2	3	sp|P05387|RLA2_HUMAN	RPLP2	11.66	3.6472
2	3	sp|Q9Y224|CN166_HUMAN	C14orf166	28.05	3.5919
2	3	sp|P09661|RU2A_HUMAN	SNRPA1	28.4	3.54
2	3	sp|Q14157|UBP2L_HUMAN	UBAP2L	114.47	3.3928
2	3	sp|P51648|AL3A2_HUMAN	ALDH3A2	54.81	3.3436
2	3	sp|P24534|EF1B_HUMAN	EEF1B2	24.75	3.3189
2	3	sp|Q01813|PFKAP_HUMAN	PFKP	85.54	3.252
2	3	sp|Q13748|TBA3C_HUMAN	TUBA3C	49.93	3.2265
2	3	sp|O43592|XPOT_HUMAN	XPOT	109.89	3.1309
2	3	sp|O43252|PAPS1_HUMAN	PAPSS1	70.79	3.0766
2	3	sp|Q2NL82|TSR1_HUMAN	TSR1	91.75	3.062
2	3	sp|Q32P51|RA1L2_HUMAN	HNRNPA1L	34.2	3.0162
2	3	sp|Q00535|CDK5_HUMAN	CDK5	33.28	2.9811
2	3	sp|Q9NWU5|RM22_HUMAN	MRPL22	23.63	2.8936
2	3	sp|Q9BT78|CSN4_HUMAN	COPS4	46.24	2.8376
2	3	sp|Q9H3U1|UN45A_HUMAN	UNC45A	103.01	2.8304
2	3	sp|P46783|RS10_HUMAN	RPS10	18.89	2.8165
2	3	sp|P55072|TERA_HUMAN	VCP	89.27	2.7908
2	3	sp|Q04637|IF4G1_HUMAN	EIF4G1	175.38	2.5964
2	3	sp|Q9BV44|THUM3_HUMAN	THUMPD3	56.97	2.5951
2	3	sp|P04075|ALDOA_HUMAN	ALDOA	39.4	2.568
2	3	sp|Q9NP92|RT30_HUMAN	MRPS30	50.33	2.3957
2	3	sp|P21333|FLNA_HUMAN	FLNA	280.56	2.3306
2	3	sp|P23634|AT2B4_HUMAN	ATP2B4	137.83	2.2118
2	3	sp|P52815|RM12_HUMAN	MRPL12	21.33	2.194
2	2	sp|Q9UJZ1|STML2_HUMAN	STOML2	38.51	4.891
2	2	sp|P82663|RT25_HUMAN	MRPS25	20.1	4.4026
2	2	sp|Q9Y678|COPG1_HUMAN	COPG1	97.66	4.2028
2	2	sp|P62847|RS24_HUMAN	RPS24	15.41	3.7865
2	2	sp|P52597|HNRPF_HUMAN	HNRNPF	45.64	3.7356
2	2	sp|P84098|RL19_HUMAN	RPL19	23.45	3.7321
2	2	sp|Q9NQ39|RS10L_HUMAN	RPS10P5	20.11	3.7016
2	2	sp|Q01844|EWS_HUMAN	EWSR1	68.44	3.6683
2	2	sp|P24752|THIL_HUMAN	ACAT1	45.17	3.647
2	2	sp|Q52LJ0|FA98B_HUMAN	FAM98B	37.17	3.6281
2	2	sp|P04792|HSPB1_HUMAN	HSPB1	22.77	3.6008
2	2	sp|O43143|DHX15_HUMAN	DHX15	90.88	3.5808
2	2	sp|Q92575|UBXN4_HUMAN	UBXN4	56.74	3.567
2	2	sp|Q9NYK5|RM39_HUMAN	MRPL39	38.69	3.546
2	2	sp|Q96P70|IPO9_HUMAN	IPO9	115.89	3.5453
2	2	sp|Q13509|TBB3_HUMAN	TUBB3	50.4	3.534
2	2	sp|P20042|IF2B_HUMAN	EIF2S2	38.36	3.4827
2	2	sp|O00410|IPO5_HUMAN	IPO5	123.55	3.4672
2	2	sp|P63208|SKP1_HUMAN	SKP1	18.65	3.4359
2	2	sp|Q92615|LAR4B_HUMAN	LARP4B	80.5	3.4266

2	2	sp|Q9NVI1|FANCI_HUMAN	FANCI	149.23	3.4
2	2	sp|Q04917|1433F_HUMAN	YWHAH	28.2	3.3679
2	2	sp|Q7Z4Q2|HEAT3_HUMAN	HEATR3	74.53	3.3583
2	2	sp|P61981|1433G_HUMAN	YWHAG	28.28	3.346
2	2	sp|O75179|ANR17_HUMAN	ANKRD17	274.09	3.2808
2	2	sp|Q3KQU3|MA7D1_HUMAN	MAP7D1	92.76	3.2797
2	2	sp|Q9Y5V3|MAGD1_HUMAN	MAGED1	86.11	3.2776
2	2	sp|Q9HAV4|XPO5_HUMAN	XPO5	136.22	3.1825
2	2	sp|Q02878|RL6_HUMAN	RPL6	32.71	3.1601
2	2	sp|P83731|RL24_HUMAN	RPL24	17.77	3.1596
2	2	sp|Q9H9J2|RM44_HUMAN	MRPL44	37.51	3.1452
2	2	sp|P38159|RBMX_HUMAN	RBMX	42.31	3.1179
2	2	sp|Q14257|RCN2_HUMAN	RCN2	36.85	3.1025
2	2	sp|Q58FF8|H90B2_HUMAN	HSP90AB2F	44.32	3.0727
2	2	sp|O76003|GLRX3_HUMAN	GLRX3	37.41	3.0694
2	2	sp|Q15645|PCH2_HUMAN	TRIP13	48.52	3.0675
2	2	sp|P31948|STIP1_HUMAN	STIP1	62.6	3.0565
2	2	sp|Q9P035|HACD3_HUMAN	HACD3	43.13	3.0318
2	2	sp|P12004|PCNA_HUMAN	PCNA	28.75	3.0295
2	2	sp|P42858|HD_HUMAN	HTT	347.38	3.026
2	2	sp|P43307|SSRA_HUMAN	SSR1	32.22	2.9817
2	2	sp|Q9UBX3|DIC_HUMAN	SLC25A10	31.26	2.9713
2	2	sp|P35268|RL22_HUMAN	RPL22	14.78	2.9678
2	2	sp|P27824|CALX_HUMAN	CANX	67.53	2.9592
2	2	sp|Q96KK5|H2A1H_HUMAN	HIST1H2AH	13.9	2.951
2	2	sp|P26368|U2AF2_HUMAN	U2AF2	53.47	2.9443
2	2	sp|O95747|OXSR1_HUMAN	OXSR1	57.99	2.9186
2	2	sp|O94973|AP2A2_HUMAN	AP2A2	103.89	2.9179
2	2	sp|P52294|IMA5_HUMAN	KPNA1	60.18	2.8856
2	2	sp|P06748|NPM_HUMAN	NPM1	32.55	2.8716
2	2	sp|P62195|PRS8_HUMAN	PSMC5	45.6	2.8487
2	2	sp|Q9BV20|MTNA_HUMAN	MRI1	39.13	2.8278
2	2	sp|P51570|GALK1_HUMAN	GALK1	42.25	2.807
2	2	sp|Q08J23|NSUN2_HUMAN	NSUN2	86.42	2.7839
2	2	sp|P33993|MCM7_HUMAN	MCM7	81.26	2.7835
2	2	sp|O43615|TIM44_HUMAN	TIMM44	51.32	2.742
2	2	sp|Q9NRF8|PYRG2_HUMAN	CTPS2	65.64	2.7258
2	2	sp|Q71RC2|LARP4_HUMAN	LARP4	80.55	2.7217
2	2	sp|Q9BQ52|RNZ2_HUMAN	ELAC2	92.16	2.6995
2	2	sp|Q9H2U1|DHX36_HUMAN	DHX36	114.69	2.6987
2	2	sp|Q10567|AP1B1_HUMAN	AP1B1	104.57	2.6766
2	2	sp|Q9BYN8|RT26_HUMAN	MRPS26	24.2	2.6524
2	2	sp|Q15758|AAAT_HUMAN	SLC1A5	56.56	2.6494
2	2	sp|P46459|NSF_HUMAN	NSF	82.54	2.6408
2	2	sp|O14654|IRS4_HUMAN	IRS4	133.68	2.6286
2	2	sp|Q99733|NP1L4_HUMAN	NAP1L4	42.8	2.6178
2	2	sp|O14773|TPP1_HUMAN	TPP1	61.21	2.5633

2	2	sp|P08865|RSSA_HUMAN	RPSA	32.83	2.5539
2	2	sp|O14744|ANM5_HUMAN	PRMT5	72.64	2.5324
2	2	sp|Q13162|PRDX4_HUMAN	PRDX4	30.52	2.529
2	2	sp|P48047|ATPO_HUMAN	ATP5O	23.26	2.5277
2	2	sp|Q53H12|AGK_HUMAN	AGK	47.11	2.517
2	2	sp|P35579|MYH9_HUMAN	MYH9	226.39	2.5122
2	2	sp|Q9Y676|RT18B_HUMAN	MRPS18B	29.38	2.4752
2	2	sp|Q13642|FHL1_HUMAN	FHL1	36.24	2.4681
2	2	sp|Q92526|TCPW_HUMAN	CCT6B	57.78	2.4558
2	2	sp|P39656|OST48_HUMAN	DDOST	50.77	2.4247
2	2	sp|P46777|RL5_HUMAN	RPL5	34.34	2.4241
2	2	sp|P62280|RS11_HUMAN	RPS11	18.42	2.405
2	2	sp|Q96L21|RL10L_HUMAN	RPL10L	24.5	2.3903
2	2	sp|Q7Z2W4|ZCCHV_HUMAN	ZC3HAV1	101.37	2.3769
2	2	sp|Q99575|POP1_HUMAN	POP1	114.64	2.3748
2	2	sp|Q14152|EIF3A_HUMAN	EIF3A	166.47	2.3385
2	2	sp|Q15738|NSDHL_HUMAN	NSDHL	41.87	2.3118
2	2	sp|P62820|RAB1A_HUMAN	RAB1A	22.66	2.3084
2	2	sp|P51798|CLCN7_HUMAN	CLCN7	88.62	2.3041
2	2	sp|Q9UNM6|PSD13_HUMAN	PSMD13	42.92	2.2961
2	2	sp|P48444|COPD_HUMAN	ARCN1	57.17	2.2921
2	2	sp|O14734|ACOT8_HUMAN	ACOT8	35.89	2.2205
2	2	sp|Q6PKG0|LARP1_HUMAN	LARP1	123.43	2.2145
2	2	sp|P42766|RL35_HUMAN	RPL35	14.54	2.1581
2	2	sp|Q9Y3F4|STRAP_HUMAN	STRAP	38.41	2.1402
2	2	sp|Q9BRL6|SRSF8_HUMAN	SRSF8	32.27	2.1328
2	2	sp|P22090|RS4Y1_HUMAN	RPS4Y1	29.44	2.0945
2	2	sp|P50502|F10A1_HUMAN	ST13	41.31	2.0835
1	16	sp|Q9UHH9|IP6K2_HUMAN	IP6K2	49.15	3.3563
1	8	sp|P0DN76|U2AF5_HUMAN	U2AF1L5	27.85	2.5456
1	4	sp|P55795|HNRH2_HUMAN	HNRNPH2	49.23	3.9709
1	4	sp|P62306|RUXF_HUMAN	SNRPF	9.72	3.5306
1	3	sp|Q14C86|GAPD1_HUMAN	GAPVD1	164.88	2.4309
1	2	sp|P55060|XPO2_HUMAN	CSE1L	110.35	5.2264
1	2	sp|Q02978|M2OM_HUMAN	SLC25A11	34.04	4.5308
1	2	sp|P13929|ENOB_HUMAN	ENO3	46.96	3.8778
1	2	sp|P34896|GLYC_HUMAN	SHMT1	53.05	3.8663
1	2	sp|Q16822|PCKGM_HUMAN	PCK2	70.68	3.7104
1	2	sp|P09874|PARP1_HUMAN	PARP1	113.01	3.6766
1	2	tr|Q53F64|Q53F64_HUMAN		35.97	3.6037
1	2	sp|P62333|PRS10_HUMAN	PSMC6	44.15	3.5782
1	2	sp|O75306|NDUS2_HUMAN	NDUFS2	52.51	3.5305
1	2	sp|P62854|RS26_HUMAN	RPS26	13.01	3.294
1	2	sp|Q9ULX6|AKP8L_HUMAN	AKAP8L	71.6	3.2792
1	2	sp|Q9GZU8|F192A_HUMAN	FAM192A	28.89	3.2659
1	2	sp|Q9P287|BCCIP_HUMAN	BCCIP	35.96	3.1949
1	2	sp|Q15051|IQCB1_HUMAN	IQCB1	68.89	3.1211

1	2	sp|P06744|G6PI_HUMAN	GPI	63.11	3.0884
1	2	sp|P05386|RLA1_HUMAN	RPLP1	11.51	2.978
1	2	sp|Q16629|SRSF7_HUMAN	SRSF7	27.35	2.816
1	2	sp|P49257|LMAN1_HUMAN	LMAN1	57.51	2.8141
1	2	sp|P22392|NDKB_HUMAN	NME2	17.29	2.7792
1	2	sp|P55884|EIF3B_HUMAN	EIF3B	92.42	2.7757
1	2	tr|A0A024RBS1|A0A024RBS1_HUMAN	GCN1L1	266.71	2.7332
1	2	sp|P62857|RS28_HUMAN	RPS28	7.84	2.7269
1	2	sp|Q8IXM3|RM41_HUMAN	MRPL41	15.37	2.7215
1	2	sp|Q13084|RM28_HUMAN	MRPL28	30.14	2.7172
1	2	sp|O95104|SFR15_HUMAN	SCAF4	125.79	2.5496
1	2	sp|P13804|ETFA_HUMAN	ETFA	35.06	2.521
1	2	sp|Q06830|PRDX1_HUMAN	PRDX1	22.1	2.4096
1	2	sp|Q99653|CHP1_HUMAN	CHP1	22.44	2.3638
1	2	sp|Q14498|RBM39_HUMAN	RBM39	59.34	2.266
1	2	sp|Q9Y2R5|RT17_HUMAN	MRPS17	14.49	2.0162
1	1	sp|P0DN79|CBSL_HUMAN	CBSL	60.55	5.309
1	1	sp|Q01130|SRSF2_HUMAN	SRSF2	25.46	5.2847
1	1	sp|Q9BQ67|GRWD1_HUMAN	GRWD1	49.39	4.8773
1	1	sp|P27635|RL10_HUMAN	RPL10	24.59	4.749
1	1	sp|Q9BYD2|RM09_HUMAN	MRPL9	30.22	4.4497
1	1	sp|O43719|HTSF1_HUMAN	HTATSF1	85.8	4.4083
1	1	sp|P27348|1433T_HUMAN	YWHAQ	27.75	4.2987
1	1	sp|P63104|1433Z_HUMAN	YWHAZ	27.73	4.247
1	1	sp|Q96FW1|OTUB1_HUMAN	OTUB1	31.26	4.2466
1	1	sp|P61163|ACTZ_HUMAN	ACTR1A	42.59	4.1981
1	1	sp|P50213|IDH3A_HUMAN	IDH3A	39.57	4.1814
1	1	sp|Q13243|SRSF5_HUMAN	SRSF5	31.25	4.1174
1	1	sp|Q8NEZ5|FBX22_HUMAN	FBXO22	44.48	4.1136
1	1	sp|P57088|TMM33_HUMAN	TMEM33	27.96	4.1027
1	1	tr|Q8IWP6|Q8IWP6_HUMAN		49.72	4.074
1	1	sp|Q13617|CUL2_HUMAN	CUL2	86.93	4.0713
1	1	sp|P55010|IF5_HUMAN	EIF5	49.19	4.0535
1	1	sp|P19525|E2AK2_HUMAN	EIF2AK2	62.06	4.0159
1	1	sp|P07237|PDIA1_HUMAN	P4HB	57.08	3.9787
1	1	tr|Q5CAQ4|Q5CAQ4_HUMAN	TRAP1	57.2	3.9769
1	1	sp|Q15393|SF3B3_HUMAN	SF3B3	135.49	3.968
1	1	sp|Q96JC9|EAF1_HUMAN	EAF1	29.02	3.9498
1	1	sp|O43242|PSMD3_HUMAN	PSMD3	60.94	3.9462
1	1	sp|Q13057|COASY_HUMAN	COASY	62.29	3.9459
1	1	sp|P19367|HXK1_HUMAN	HK1	102.42	3.9456
1	1	sp|O14818|PSA7_HUMAN	PSMA7	27.87	3.9447
1	1	sp|Q9HC07|TM165_HUMAN	TMEM165	34.88	3.9416
1	1	sp|Q12904|AIMP1_HUMAN	AIMP1	34.33	3.9307
1	1	sp|O15131|IMA6_HUMAN	KPNA5	60.31	3.9135
1	1	sp|P62266|RS23_HUMAN	RPS23	15.8	3.8529
1	1	sp|P30837|AL1B1_HUMAN	ALDH1B1	57.17	3.8376

1	1	sp|P62424|RL7A_HUMAN	RPL7A	29.98	3.8257
1	1	sp|P62913|RL11_HUMAN	RPL11	20.24	3.8098
1	1	sp|Q4G0J3|LARP7_HUMAN	LARP7	66.86	3.809
1	1	sp|O60783|RT14_HUMAN	MRPS14	15.13	3.8002
1	1	sp|Q9UI30|TR112_HUMAN	TRMT112	14.19	3.7942
1	1	sp|Q96A33|CCD47_HUMAN	CCDC47	55.84	3.7921
1	1	sp|Q15024|EXOS7_HUMAN	EXOSC7	31.8	3.7902
1	1	sp|P09211|GSTP1_HUMAN	GSTP1	23.34	3.7486
1	1	sp|Q9NWU2|GID8_HUMAN	GID8	26.73	3.7419
1	1	sp|Q00610|CLH1_HUMAN	CLTC	191.49	3.7211
1	1	sp|Q13765|NACA_HUMAN	NACA	23.37	3.7204
1	1	sp|Q9Y262|EIF3L_HUMAN	EIF3L	66.68	3.6666
1	1	sp|P13995|MTDC_HUMAN	MTHFD2	37.87	3.6658
1	1	sp|P63010|AP2B1_HUMAN	AP2B1	104.49	3.6658
1	1	sp|Q1KMD3|HNRL2_HUMAN	HNRNPUL2	85.05	3.6647
1	1	sp|O14983|AT2A1_HUMAN	ATP2A1	110.18	3.6645
1	1	sp|Q9H845|ACAD9_HUMAN	ACAD9	68.72	3.6497
1	1	sp|A1L0T0|ILVBL_HUMAN	ILVBL	67.82	3.6486
1	1	sp|P30154|2AAB_HUMAN	PPP2R1B	66.17	3.6292
1	1	sp|Q93034|CUL5_HUMAN	CUL5	90.9	3.5948
1	1	sp|P12235|ADT1_HUMAN	SLC25A4	33.04	3.5915
1	1	sp|Q9BQA1|MEP50_HUMAN	WDR77	36.7	3.5891
1	1	sp|P42677|RS27_HUMAN	RPS27	9.45	3.5842
1	1	sp|O00178|GTPB1_HUMAN	GTPBP1	72.41	3.5603
1	1	sp|Q96HN2|SAHH3_HUMAN	AHCYL2	66.68	3.5503
1	1	sp|Q9NZB2|F120A_HUMAN	FAM120A	121.81	3.5198
1	1	sp|O43164|PJA2_HUMAN	PJA2	78.17	3.5083
1	1	sp|Q6ZRP7|QSOX2_HUMAN	QSOX2	77.48	3.4607
1	1	sp|O00231|PSD11_HUMAN	PSMD11	47.43	3.4566
1	1	sp|Q15349|KS6A2_HUMAN	RPS6KA2	83.19	3.4491
1	1	sp|Q86Y56|DAAF5_HUMAN	DNAAF5	93.46	3.4156
1	1	sp|Q15293|RCN1_HUMAN	RCN1	38.87	3.4153
1	1	sp|Q8WXX5|DNJC9_HUMAN	DNAJC9	29.89	3.4032
1	1	sp|P67775|PP2AA_HUMAN	PPP2CA	35.57	3.4008
1	1	sp|Q14738|2A5D_HUMAN	PPP2R5D	69.95	3.3955
1	1	sp|Q9UBQ5|EIF3K_HUMAN	EIF3K	25.04	3.393
1	1	sp|Q969V3|NCLN_HUMAN	NCLN	62.93	3.3806
1	1	sp|Q9H2W6|RM46_HUMAN	MRPL46	31.69	3.3802
1	1	sp|P42285|SK2L2_HUMAN	SKIV2L2	117.73	3.3522
1	1	sp|O15371|EIF3D_HUMAN	EIF3D	63.93	3.3518
1	1	sp|O43837|IDH3B_HUMAN	IDH3B	42.16	3.3482
1	1	sp|Q6P158|DHX57_HUMAN	DHX57	155.51	3.3104
1	1	sp|P23284|PPIB_HUMAN	PPIB	23.73	3.3079
1	1	tr|A0A024R2I7|A0A024R2I7_HUMAN	RAD18	56.16	3.3032
1	1	sp|O95071|UBR5_HUMAN	UBR5	309.16	3.3002
1	1	sp|Q96HR8|NAF1_HUMAN	NAF1	53.68	3.2908
1	1	sp|Q9UNF1|MAGD2_HUMAN	MAGED2	64.91	3.2847

1	1	sp|P42167|LAP2B_HUMAN	TMPO	50.64	3.2813
1	1	sp|P05166|PCCB_HUMAN	PCCB	58.18	3.274
1	1	sp|Q9Y2S7|PDIP2_HUMAN	POLDIP2	42.01	3.2729
1	1	sp|P61626|LYSC_HUMAN	LYZ	16.53	3.2725
1	1	sp|Q8IUR7|ARMC8_HUMAN	ARMC8	75.46	3.2716
1	1	sp|P60953|CDC42_HUMAN	CDC42	21.25	3.263
1	1	sp|Q8NC60|NOA1_HUMAN	NOA1	78.41	3.2587
1	1	sp|P51812|KS6A3_HUMAN	RPS6KA3	83.68	3.2242
1	1	sp|Q8NF37|PCAT1_HUMAN	LPCAT1	59.11	3.204
1	1	sp|P82921|RT21_HUMAN	MRPS21	10.73	3.1984
1	1	sp|Q3ZCQ8|TIM50_HUMAN	TIMM50	39.62	3.1955
1	1	sp|P24928|RPB1_HUMAN	POLR2A	217.04	3.1811
1	1	sp|Q9BTE6|AASD1_HUMAN	AARSD1	45.45	3.1767
1	1	sp|Q92522|H1X_HUMAN	H1FX	22.47	3.1755
1	1	sp|Q659C4|LAR1B_HUMAN	LARP1B	105.26	3.1577
1	1	sp|Q9UNL2|SSRG_HUMAN	SSR3	21.07	3.1546
1	1	sp|Q05639|EF1A2_HUMAN	EEF1A2	50.44	3.1414
1	1	sp|Q8TAA3|PSA7L_HUMAN	PSMA8	28.51	3.1382
1	1	sp|P13807|GYS1_HUMAN	GYS1	83.73	3.1335
1	1	sp|P62314|SMD1_HUMAN	SNRPD1	13.27	3.1332
1	1	sp|Q14568|HS902_HUMAN	HSP90AA2I	39.34	3.1281
1	1	sp|O95376|ARI2_HUMAN	ARIH2	57.78	3.1142
1	1	sp|P50454|SERPH_HUMAN	SERPINH1	46.41	3.0933
1	1	sp|Q92769|HDAC2_HUMAN	HDAC2	55.33	3.0895
1	1	sp|Q9NVH0|EXD2_HUMAN	EXD2	70.31	3.0755
1	1	sp|Q96E29|MTEF3_HUMAN	MTERF3	47.94	3.0739
1	1	sp|Q15436|SC23A_HUMAN	SEC23A	86.11	3.0663
1	1	sp|Q9NRX2|RM17_HUMAN	MRPL17	20.04	3.0647
1	1	sp|P56134|ATPK_HUMAN	ATP5J2	10.91	3.0554
1	1	sp|Q16891|MIC60_HUMAN	IMMT	83.63	3.0289
1	1	sp|O75817|POP7_HUMAN	POP7	15.64	3.0264
1	1	sp|Q9BVC6|TM109_HUMAN	TMEM109	26.19	3.0248
1	1	sp|O00487|PSDE_HUMAN	PSMD14	34.55	3.0023
1	1	sp|Q9BQG0|MBB1A_HUMAN	MYBBP1A	148.76	3.0011
1	1	sp|Q9UHY1|NRBP_HUMAN	NRBP1	59.81	2.9999
1	1	sp|Q9ULK4|MED23_HUMAN	MED23	156.37	2.9798
1	1	sp|Q8IVS2|FABD_HUMAN	MCAT	42.93	2.9781
1	1	sp|Q9NTK5|OLA1_HUMAN	OLA1	44.72	2.9751
1	1	sp|Q15139|KPCD1_HUMAN	PRKD1	101.64	2.9709
1	1	sp|O75419|CDC45_HUMAN	CDC45	65.53	2.9694
1	1	sp|A6NHL2|TBAL3_HUMAN	TUBAL3	49.88	2.9683
1	1	sp|P54105|ICLN_HUMAN	CLNS1A	26.2	2.9663
1	1	sp|Q9UJW0|DCTN4_HUMAN	DCTN4	52.3	2.9635
1	1	sp|Q9BYD6|RM01_HUMAN	MRPL1	36.89	2.9622
1	1	sp|Q9UPN9|TRI33_HUMAN	TRIM33	122.46	2.9588
1	1	sp|P41743|KPCI_HUMAN	PRKCI	68.22	2.945
1	1	sp|Q13724|MOGS_HUMAN	MOGS	91.86	2.9375

1	1	sp|P30153|2AAA_HUMAN	PPP2R1A	65.27	2.9345
1	1	sp|Q9BYC9|RM20_HUMAN	MRPL20	17.43	2.9317
1	1	sp|Q16630|CPSF6_HUMAN	CPSF6	59.17	2.9245
1	1	sp|Q13309|SKP2_HUMAN	SKP2	47.73	2.9221
1	1	sp|P53985|MOT1_HUMAN	SLC16A1	53.91	2.9205
1	1	sp|Q14444|CAPR1_HUMAN	CAPRIN1	78.32	2.9187
1	1	sp|Q09161|NCBP1_HUMAN	NCBP1	91.78	2.9027
1	1	sp|Q9NQ50|RM40_HUMAN	MRPL40	24.48	2.885
1	1	sp|Q96RQ3|MCCA_HUMAN	MCCC1	80.42	2.8559
1	1	sp|Q15334|L2GL1_HUMAN	LLGL1	115.35	2.8508
1	1	sp|Q14839|CHD4_HUMAN	CHD4	217.87	2.8484
1	1	sp|Q13405|RM49_HUMAN	MRPL49	19.19	2.834
1	1	sp|P36578|RL4_HUMAN	RPL4	47.67	2.8233
1	1	sp|Q5SSJ5|HP1B3_HUMAN	HP1BP3	61.17	2.8193
1	1	sp|P50402|EMD_HUMAN	EMD	28.98	2.8167
1	1	sp|P43897|EFTS_HUMAN	TSFM	35.37	2.7992
1	1	sp|Q5JNZ5|RS26L_HUMAN	RPS26P11	12.99	2.7815
1	1	sp|P46776|RL27A_HUMAN	RPL27A	16.55	2.7692
1	1	sp|P12268|IMDH2_HUMAN	IMPDH2	55.77	2.7629
1	1	sp|Q8NHH9|ATLA2_HUMAN	ATL2	66.19	2.7628
1	1	sp|Q13618|CUL3_HUMAN	CUL3	88.87	2.7592
1	1	sp|P33991|MCM4_HUMAN	MCM4	96.5	2.754
1	1	sp|Q9NZ01|TECR_HUMAN	TECR	36.01	2.7473
1	1	sp|P31689|DNJA1_HUMAN	DNAJA1	44.84	2.7449
1	1	sp|P53007|TXTP_HUMAN	SLC25A1	33.99	2.7437
1	1	sp|Q15021|CND1_HUMAN	NCAPD2	157.08	2.7283
1	1	sp|Q8N1F7|NUP93_HUMAN	NUP93	93.43	2.7276
1	1	sp|P08579|RU2B_HUMAN	SNRPB2	25.47	2.7271
1	1	sp|O75155|CAND2_HUMAN	CAND2	135.17	2.7247
1	1	sp|Q9NXF7|DCA16_HUMAN	DCAF16	24.18	2.716
1	1	sp|Q14978|NOLC1_HUMAN	NOLC1	73.56	2.7151
1	1	sp|O76021|RL1D1_HUMAN	RSL1D1	54.94	2.7067
1	1	sp|Q92945|FUBP2_HUMAN	KHSRP	73.07	2.704
1	1	sp|P82914|RT15_HUMAN	MRPS15	29.82	2.7035
1	1	sp|Q8WVX9|FACR1_HUMAN	FAR1	59.32	2.6936
1	1	sp|Q9NX58|LYAR_HUMAN	LYAR	43.59	2.6936
1	1	sp|Q66K14|TBC9B_HUMAN	TBC1D9B	140.44	2.688
1	1	sp|P02786|TFR1_HUMAN	TFRC	84.82	2.6815
1	1	sp|Q02539|H11_HUMAN	HIST1H1A	21.83	2.6755
1	1	sp|Q9BZF1|OSBL8_HUMAN	OSBPL8	101.13	2.6565
1	1	sp|Q969Z0|TBRG4_HUMAN	TBRG4	70.69	2.6382
1	1	sp|P22102|PUR2_HUMAN	GART	107.7	2.6341
1	1	sp|Q12874|SF3A3_HUMAN	SF3A3	58.81	2.6242
1	1	sp|P18124|RL7_HUMAN	RPL7	29.21	2.5897
1	1	sp|Q9NYY8|FAKD2_HUMAN	FASTKD2	81.41	2.5859
1	1	sp|P23526|SAHH_HUMAN	AHCY	47.69	2.5856
1	1	sp|Q14197|ICT1_HUMAN	MRPL58	23.62	2.5806

1	1	sp|P10398|ARAF_HUMAN	ARAF	67.54	2.5794
1	1	sp|O95163|ELP1_HUMAN	IKBKAP	150.16	2.5664
1	1	sp|Q5VYS8|TUT7_HUMAN	ZCCHC6	171.12	2.5609
1	1	sp|P68366|TBA4A_HUMAN	TUBA4A	49.89	2.556
1	1	sp|P62304|RUXE_HUMAN	SNRPE	10.8	2.551
1	1	sp|Q6PIW4|FIGL1_HUMAN	FIGNL1	74.03	2.5509
1	1	sp|Q8IXQ4|GPAM1_HUMAN	GPALPP1	38.12	2.5473
1	1	sp|P53779|MK10_HUMAN	MAPK10	52.55	2.535
1	1	sp|Q8TBP6|S2540_HUMAN	SLC25A40	38.1	2.5303
1	1	sp|Q9Y285|SYFA_HUMAN	FARSA	57.53	2.5194
1	1	sp|Q5H9R7|PP6R3_HUMAN	PPP6R3	97.61	2.519
1	1	sp|Q8N5N7|RM50_HUMAN	MRPL50	18.31	2.5149
1	1	sp|P62310|LSM3_HUMAN	LSM3	11.84	2.49
1	1	sp|P43243|MATR3_HUMAN	MATR3	94.56	2.4878
1	1	sp|P62316|SMD2_HUMAN	SNRPD2	13.52	2.4837
1	1	sp|Q9Y5A9|YTHD2_HUMAN	YTHDF2	62.3	2.4777
1	1	sp|P11216|PYGB_HUMAN	PYGB	96.63	2.4617
1	1	sp|P62273|RS29_HUMAN	RPS29	6.67	2.4335
1	1	sp|Q15517|CDSN_HUMAN	CDSN	51.49	2.4303
1	1	sp|Q9Y3D3|RT16_HUMAN	MRPS16	15.34	2.43
1	1	sp|P40937|RFC5_HUMAN	RFC5	38.47	2.419
1	1	sp|Q99640|PMYT1_HUMAN	PKMYT1	54.49	2.4169
1	1	sp|P22234|PUR6_HUMAN	PAICS	47.05	2.4161
1	1	sp|O00442|RTCA_HUMAN	RTCA	39.31	2.405
1	1	sp|P84103|SRSF3_HUMAN	SRSF3	19.32	2.4047
1	1	sp|Q86U44|MTA70_HUMAN	METTL3	64.43	2.404
1	1	tr|A8K7N0|A8K7N0_HUMAN		23.63	2.4023
1	1	sp|Q86U38|NOP9_HUMAN	NOP9	69.39	2.3888
1	1	sp|Q8TEX9|IPO4_HUMAN	IPO4	118.64	2.3811
1	1	sp|P31946|1433B_HUMAN	YWHAB	28.06	2.3778
1	1	sp|Q9BRP8|PYM1_HUMAN	PYM1	22.64	2.3658
1	1	sp|P13797|PLST_HUMAN	PLS3	70.77	2.3582
1	1	sp|Q8N5K1|CISD2_HUMAN	CISD2	15.27	2.3543
1	1	sp|Q9Y5L0|TNPO3_HUMAN	TNPO3	104.14	2.3497
1	1	sp|Q9H1A4|APC1_HUMAN	ANAPC1	216.36	2.3464
1	1	sp|P09001|RM03_HUMAN	MRPL3	38.61	2.3454
1	1	sp|Q86U42|PABP2_HUMAN	PABPN1	32.73	2.3365
1	1	sp|P19387|RPB3_HUMAN	POLR2C	31.42	2.335
1	1	sp|Q5T4S7|UBR4_HUMAN	UBR4	573.48	2.3245
1	1	sp|P61353|RL27_HUMAN	RPL27	15.79	2.3175
1	1	sp|O94952|FBX21_HUMAN	FBXO21	72.22	2.3056
1	1	sp|Q07065|CKAP4_HUMAN	CKAP4	65.98	2.2991
1	1	sp|P53621|COPA_HUMAN	COPA	138.26	2.296
1	1	sp|Q9H0U6|RM18_HUMAN	MRPL18	20.56	2.2921
1	1	tr|B7Z645|B7Z645_HUMAN	SYNCRIP	52.01	2.2895
1	1	sp|P30876|RPB2_HUMAN	POLR2B	133.81	2.2854
1	1	sp|O00505|IMA4_HUMAN	KPNA3	57.77	2.2762

1	1	sp|Q8N163|CCAR2_HUMAN	CCAR2	102.84	2.2757
1	1	sp|P00441|SODC_HUMAN	SOD1	15.93	2.2722
1	1	sp|Q13310|PABP4_HUMAN	PABPC4	70.74	2.2699
1	1	sp|Q96K76|UBP47_HUMAN	USP47	157.21	2.261
1	1	sp|O00483|NDUA4_HUMAN	NDUFA4	9.36	2.2589
1	1	sp|Q15388|TOM20_HUMAN	TOMM20	16.29	2.2527
1	1	sp|Q96B54|ZN428_HUMAN	ZNF428	20.47	2.241
1	1	sp|Q9BSD7|NTPCR_HUMAN	NTPCR	20.7	2.2353
1	1	sp|O95433|AHSA1_HUMAN	AHSA1	38.25	2.2319
1	1	sp|P01876|IGHA1_HUMAN	IGHA1	37.63	2.2241
1	1	sp|P11172|UMPS_HUMAN	UMPS	52.19	2.2133
1	1	sp|P05165|PCCA_HUMAN	PCCA	80.01	2.208
1	1	sp|Q9NWK9|BCD1_HUMAN	ZNHIT6	53.88	2.2038
1	1	sp|Q9BTD8|RBM42_HUMAN	RBM42	50.38	2.1974
1	1	sp|P42224|STAT1_HUMAN	STAT1	87.28	2.1757
1	1	sp|P00558|PGK1_HUMAN	PGK1	44.59	2.1732
1	1	sp|P17980|PRS6A_HUMAN	PSMC3	49.17	2.1677
1	1	sp|P51991|ROA3_HUMAN	HNRNPA3	39.57	2.1584
1	1	sp|Q13247|SRSF6_HUMAN	SRSF6	39.56	2.1381
1	1	sp|Q9NTJ5|SAC1_HUMAN	SACM1L	66.92	2.1306
1	1	sp|Q92621|NU205_HUMAN	NUP205	227.78	2.1303
1	1	sp|Q9BTT6|LRRC1_HUMAN	LRRC1	59.2	2.1252
1	1	sp|Q9Y4X5|ARI1_HUMAN	ARIH1	64.08	2.1228
1	1	sp|O43847|NRDC_HUMAN	NRDC	131.49	2.1222
1	1	sp|Q6P161|RM54_HUMAN	MRPL54	15.81	2.1186
1	1	sp|P11498|PYC_HUMAN	PC	129.55	2.1184
1	1	sp|P10155|RO60_HUMAN	TROVE2	60.63	2.1138
1	1	sp|Q9NSE4|SYIM_HUMAN	IARS2	113.72	2.0964
1	1	sp|Q14739|LBR_HUMAN	LBR	70.66	2.0806
1	1	sp|O60684|IMA7_HUMAN	KPNA6	59.99	2.0792
1	1	sp|Q9UBM7|DHCR7_HUMAN	DHCR7	54.45	2.0713
1	1	sp|Q9UNE7|CHIP_HUMAN	STUB1	34.83	2.0585
1	1	sp|P78344|IF4G2_HUMAN	EIF4G2	102.3	2.0463
1	1	sp|Q8WUK0|PTPM1_HUMAN	PTPMT1	22.83	2.0417
1	1	sp|Q9UBF2|COPG2_HUMAN	COPG2	97.56	2.0399
1	1	sp|O75369|FLNB_HUMAN	FLNB	277.99	2.0373
1	1	sp|Q96AG4|LRC59_HUMAN	LRRC59	34.91	2.0283
